# The timing of the initial collision between the South and North China blocks constraining from the sediments in the eastern Sichuan Basin

**DOI:** 10.1038/s41598-023-49498-z

**Published:** 2023-12-16

**Authors:** Tianjia Liu, Zongquan Hu, Dianwei Zhang, Shuangjian Li, Chuanjie Cheng, Lingfang Zhou, Guanping Wang, Xunlian Wang, Zhentao Wang

**Affiliations:** 1 State Key Laboratory of Shale Oil and Gas Enrichment Mechanisms and Effective Development, Beijing, 100083 China; 2 School of the Earth Sciences and Resources, China University of Geosciences, Beijing, 100083 China; 3 Sinopec Key Laboratory of Shale Oil/Gas Exploration and Production Technology, Beijing, China; 4 Key Laboratory of Metallogeny and Mineral Assessment, Institute of Mineral Resources, Chinese Academy of Geological Sciences, Beijing, 100037 China

**Keywords:** Geochemistry, Geology

## Abstract

In this study, detrital zircon U–Pb geochronology, trace element and Hf isotopic compositional data from the Early-Middle Triassic clastic rocks in the eastern Sichuan Basin were obtained to distinguish the sediment provenance and constrain the timing of the initial collision between the South China and North China blocks. Detrital zircons from the Early Triassic Feixianguan Formation clastic rocks yield one major age peak at 2476 Ma and three minor age peaks at 1886, 802 and 304 Ma. These detrital zircons may be derived from the South China Block. Detrital zircons from the Early Triassic Jialingjiang Formation clastic rocks yield multiple age peaks at 979, 856, 392 and 269 Ma, indicating a mixed sediment provenance from the South China Block and Qinling Orogenic Belt. This is the first appearance of the detritus with the Qinling Orogenic Belt affinity in the eastern Sichuan Basin. Detrital zircons from the Middle Triassic Leikoupo Formation clastic rocks yield two centralized age peaks at 447 and ca. 245 Ma. These zircons may mainly be derived from the Qinling Orogenic Belt. The results indicate an abrupt change in the detrital zircon U–Pb provenance from the South China Block to the Qinling Orogenic Belt during the Early-Middle Triassic. Integrating the provenance change and other geological characteristics, we suggest that the initial collision in the eastern Qinling Orogenic Belt occurred in the Early Triassic.

## Introduction

Continental-continental collision is one of the most essential geologic processes in the Earth's evaluation and has contributed to the formation of many large mountain ranges^1^. The collision between the South China and North China blocks played a significant effect on the amalgamation of eastern Asia and contributed to the formation of the Central Orogenic Belt in China^2,3^. The timing of plate collision between the South China and Nouth China blocks is significant for understanding the structural evolution of the China continent and even the eastern Asia. Numerous studies based on several pieces of evidence, including palaeomagnetism, stratigraphy, detrital zircon geochronology, metamorphic events, and the formation of syn-collisional granitoids, have revealed the initial timing of the continental collision between the two blocks in recent decades. However, the timing remains highly debated and varies from the Late Permian to the Late Triassic^4–13^. This controversy obviously stems from the following aspects. The collision between the two blocks underwent a long-term process from initial collision to comprehensive collision^5,14^, which generated different timing of the collision in different positions. Second, scholars have used different approaches and definitions of the initial collision to constrain the timings and have obtained entirely different collision timings. The initial collision in this study is defined as the disappearance of the oceanic crust between the continents and the two continental crusts went into direct contact^1^.

The continental clastic sedimentary rocks in the basin are mostly related to the exhumation and erosion of adjacent orogens^13^. Despite being a minor component of clastic sediments, detrital zircon is critical in sedimentary provenance analysis due to its physical and chemical resilience^15^. The detrital zircon age population is a key tool to reveal the geological evolution of the source regions and reconstruct palaeotectonic setting^1^. After plate collision, the detritus from the active continental margin or intermediate orogenic belt can be transported to the passive continental margin. Thus, the timing of provenance change in the passive continental margin can constrain the initial timing of collision between blocks^16^. Many provenance analysis studies based on detrital zircon provenance analysis within different regions have been conducted to constrain the timing of the initial collision between blocks^1,12,17,18^.

The Early-Middle Triassic succession in the eastern Sichuan Basin is mostly composed of interbedded marine carbonate and terrigenous clastic rocks. This clastic rock has a unique geodynamic background of continental collision, and offers an excellent window to understand the collision between the South China and North China blocks. We report the U–Pb dating, trace elements and Lu–Hf isotopic compositions from the detrital zircons of the Lower-Middle Triassic clastic rocks in the eastern Sichuan Basin. The results contribute to providing more constraints on the original sources, and better assessing the timing of the initial collision between the South China and North China blocks in the eastern Qinling Orogenic Belt.

### Geological setting and sampling

#### Qinling Orogenic Belt

**Figure 1:**
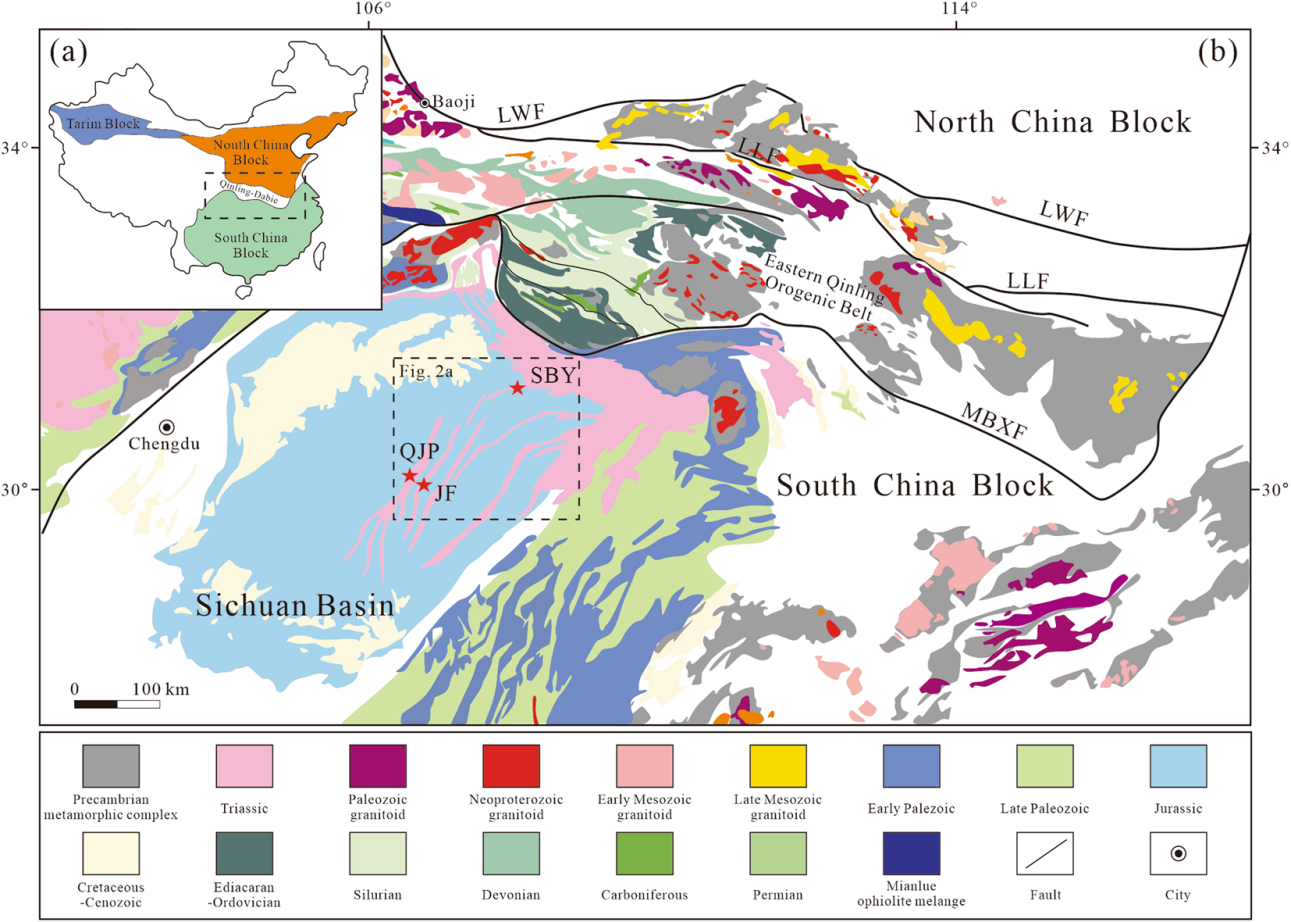
(**a**) Schematic tectonic framework of China showing the location of the Qinling Orogen^19^; (**b**) Geological map of the Sichuan Basin and adjacent orogenic belts^12^. *LWF* Lingbao–Lushan–Wuyang, *LLF* Luonan–Luanchuan fault, *MBXF* Mianxian–Bashan–Xiangfan fault.

The Qinling Orogenic Belt is situated between the South China Block in the south and the Nouth China Block in the north (Fig. 1), and stretches thousands of kilometres. It is a crucial geologic and geographic boundary in the China Continent and even the Eastern Asia Continent^20,21^. The Qinling Orogenic Belt is a large-scale compound orogenic belt that has experienced multistage oceanic subduction, continental collision and intracontinental orogeny^22,23^, and has been considered to have formed by the eventual collision between the South China and North China blocks^9,24^. This belt can be segmented into four tectonic units: the northern margin of the South China Block, South Qinling Belt, North Qinling Belt and the southern margin of the North China Block from south to north^23^. Moreover, the Qinling Orogenic Belt can be divided into the eastern Qinling Belt and western Qinling Belt by the Chengdu-Baoji railway^25^.

The southern margin of the North China Block is located between the Lingbao-Lushan-Wuyang fault and Luonan-Luanchuan fault, and has a structural affinity to the North China Block^26^. The North Qinling Belt is located between the Luonan-Luanchuan-Fangcheng fault and the Shangdan suture zone. It consists of the Precambrian basement, the Neoproterozoic and Early Palaeozoic ophiolites and volcanic-sedimentary sequences, which are unconformably overlain by the Carboniferous-Permian sedimentary successions^24^. The South Qinling Belt is located between the Shangdan suture zone and the Mianlue suture zone, and is characterised by a south-vergent imbricated thrust-fold^23^. It mostly consists of the Neoarchean basement, the Neoproterozoic volcano-sedimentary sequences, and the Ediacaran to Middle Triassic sedimentary successions^9^. It separated from the South China Block under the influence of the Mianlue Ocean during the Devonian to Early Triassic, and bordered the northern margin of the South China Block by continent–continent collision in the early Mesozoic. The northern margin of the South China is separated from the Qinling Orogenic Belt by the Mianlue-Bashan-Xiangguang fault^9^.

#### Study region and sampled horizons

The study sections are situated in the eastern Sichuan Basin and lie very close to the eastern Qinling Orogenic Belt (Fig. 1a). The continuous Triassic stratigraphic succession in the study region is mainly marine carbonate and well exposed, which includes the Early Triassic Feixianguan and Jialingjiang, Middle Triassic Leikoupo Formation, and Late Triassic Xujiahe Formation from oldest to youngest^27^. The Feixianguan Formation is mostly composed of limestone, dolomite, mudstone and siltstone, and is in conformable contact with the overlying Early Triassic Jialingjiang Formation. The Jialingjiang Formation mostly consists of carbonate-evaporite successions dominated by limestone, dolomite, and gyprock. The Leikoupo Formation is the youngest marine carbonate in the basin, which marks the final cessation of marine sedimentation^28^. This formation mainly consists of limestone and dolomite with interbedded evaporite and shale, and is marked by the so-called “mung-bean-rock” at the bottom^29^. The late Middle Indosinian movement generated the uplift of the study region, and the upper part of the Leikoupo Formation underwent denudation in varying degrees^27^. The Leikoupo Formation conformably overlies the underlying Jialingjiang Formation, and an unconformity underlies the overlying Xujiahe Formation.

**Figure 2:**
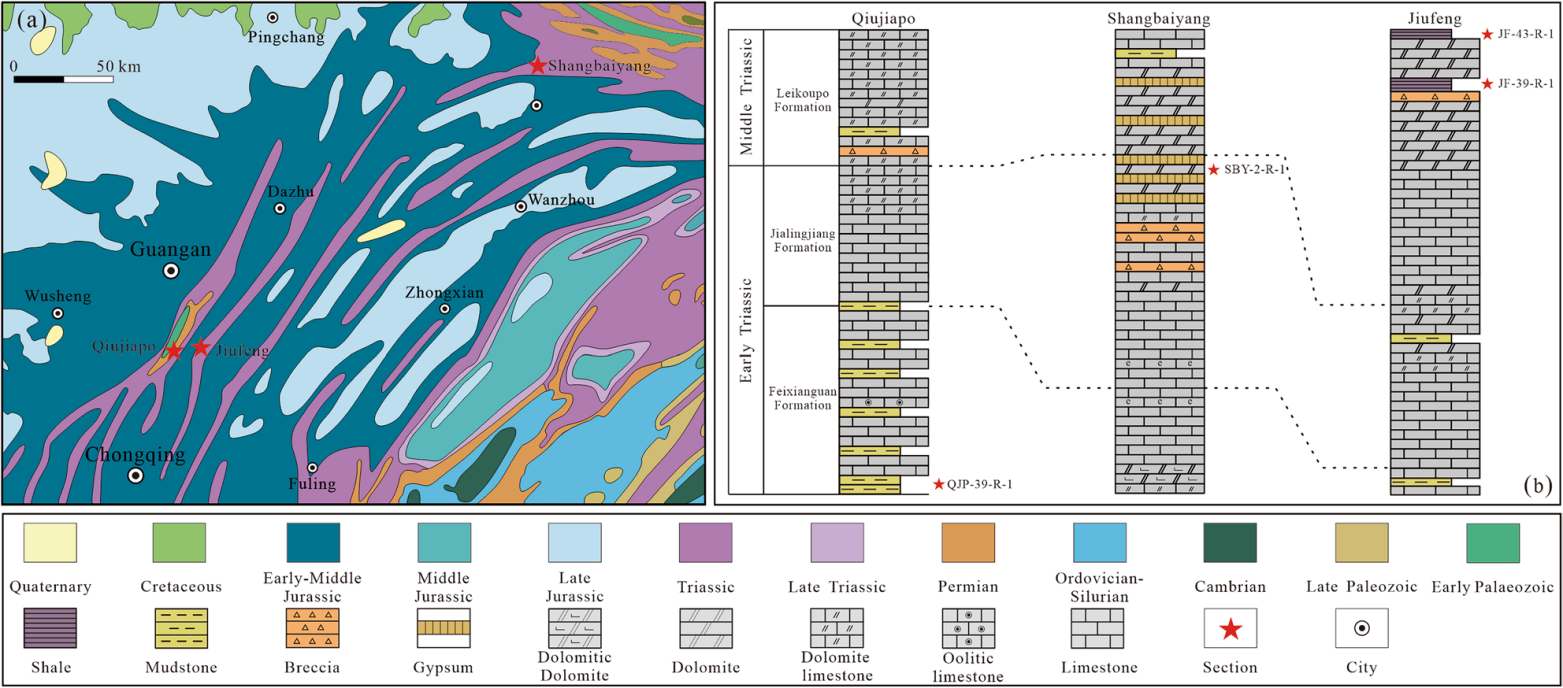
(**a**) Geological sketch map of the study region and sample locations^30^; (**b**) Cross-sections in the study region. The Shangbaiyang section is modified from^31,32^.

**Figure 3:**
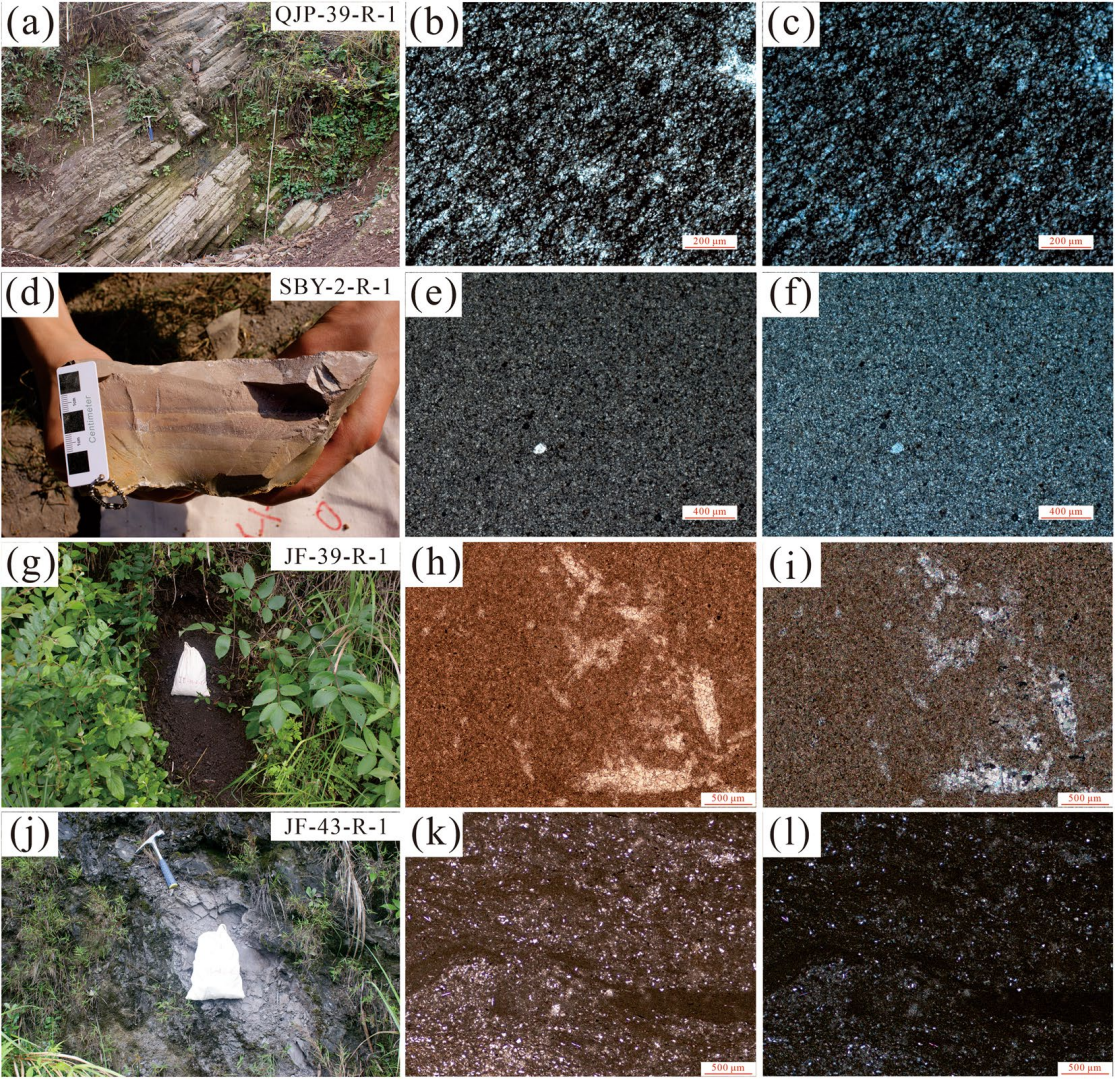
Field photographs showing the sampling locations and the photomicrographs of the samples in the study regions. (**a**–**c**) Argillaceous siltstone at the bottom of the Feixianguan Formation; (**d**–**f**) Argillaceous dolomite at the top of the Jialingjiang Formation; (**g**–**l**) Shale at the Leikoupo Formation.

A total of four representative samples were collected in the study region (Fig. 2). Sample QJP-39-R-1 is argillaceous siltstone (Fig. 3a–c) from the Early Triassic Feixianguan Formation, and was collected from the Qiujiapo section (GPS: 30° 11′ 20.226″ N, 106° 45′ 53.826″ E); sample SBY-2-R-1 is argillaceous dolomite (Fig. 3d–f) from the Early Triassic Jialingjiang Formation, and was collected from the Shangbaiyang section (GPS: 31° 22′ 5.22″ N, 108° 32′ 7.626″ E); samples JF-39-R-1 and JF-43-R-1 are shale (Fig. 3g–l) from the Middle Triassic Leikoupo Formation, and were collected from the Jiufeng section (GPS: 30° 11′ 20.226″ N, 106° 45′ 53.826″ E).

### Analytical methods

All analyses were conducted at the Institute of Mineral Resources, Chinese Academy of Geological Sciences. U–Pb isotope and trace element analysis for were conducted using Finnigan Neptune inductively coupled plasma‒mass spectrometry (ICP-MS). A spot size of 25 μm and a 10-Hz repetition rate at 2.5 J/cm^2^ were used for all analyses. Zircon 91500 was used as an external standard. The analytical procedures were the same as described by previously^33^. Age calculations and concordia diagrams were made using ISOPLOT. Zircon ages older than 1000 Ma were taken as 207Pb/206Pb ages, whereas the ages younger than 1000 Ma were taken as 206Pb/238U ages^34^. Zircon Lu‒Hf isotopic analyses were conducted using a Neptune Plus multiple collector inductively coupled plasma mass spectrometer (MC–ICP‒MS), with two different spot sizes of 32 and 60 μm. The GJ-1 was used as a reference material, and helium was used as the carrier gas. Operating circumstances and procedures were as described by previously^35^.

## Result

### Detrital zircon U–Pb ages

**Figure 4:**
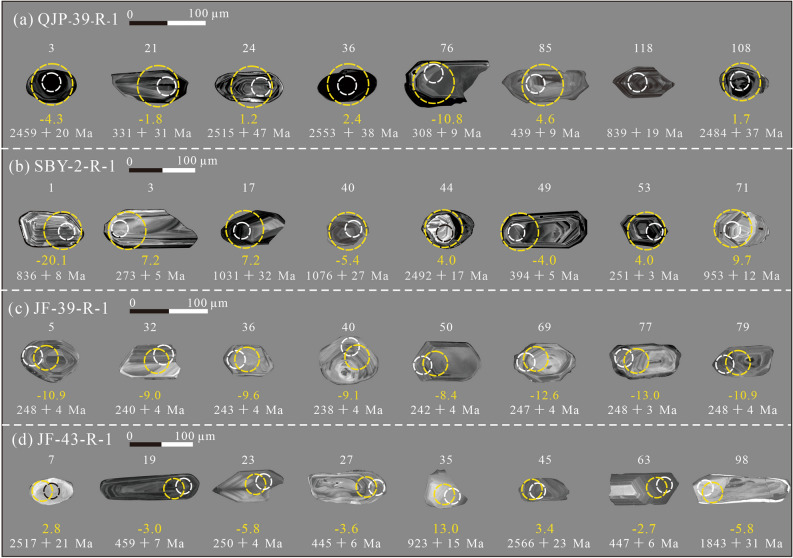
Representative cathodoluminescence (CL) images of representative zircon grains from samples and their U–Pb ages and *ε*_Hf(t)_ values. White and black circles represent the analytical sites of the zircon U–Pb ages and yellow circles represent the analytical sites of the zircon Hf isotopes.

A total of 420 zircon grains were analysed for U–Pb isotopes, 336 zircon grains with concordance exceeding 90% are used in the discussion, and the results are presented in Supplemental Table 1. The zircon grains of the four samples were mostly euhedral-subhedral and prismatic. The length of grains generally varies from 50 to 120 μm, and have aspect ratios of 1:1–4:1. Most zircon grains show magmatic oscillatory zoning indicative of magmatic origin (Fig. 4).

**Figure 5:**
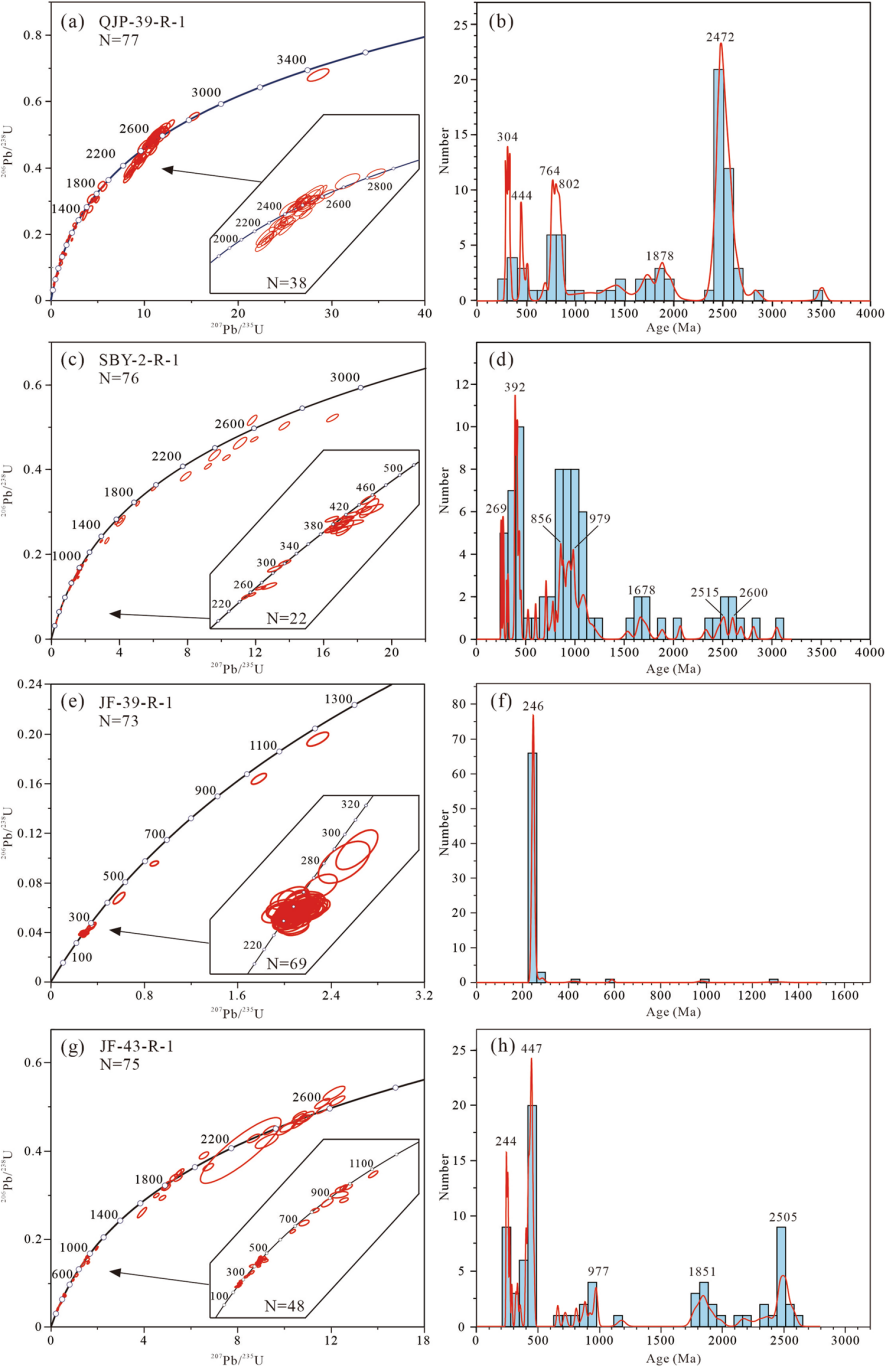
U–Pb concordia diagrams and normalized probability density distribution (red curves) of ages, showing the results of the LA-ICP–MS dating.

A total of 140 detrital zircons from sample QJP-39-R-1 were analysed, with concordant ages have a range of 3492–289 Ma. The sample yielded four groups (Fig. 5a,b): 46% of zircons occurring at 2700–2400 Ma (2472 Ma dominant peak), 15% of zircons occurring at 900–700 Ma (764 and 802 Ma dominant peaks), 11% of zircons occurring at 2000–1600 Ma (1878 Ma dominant peak), and 8% of zircons occurring at 340–280 Ma (304 Ma dominant peak).

A total of 90 detrital zircons from sample SBY-2-R-1 were analysed, and the concordant ages have a range of 3047–255 Ma. The sample yielded five groups (Fig. 5c,d): 38% of zircons occurring at 1100–800 Ma (979 Ma and 856 Ma dominant peaks), 24% of zircons occurring at 450–300 Ma (392 Ma dominant peak), 9% of zircons occurring at 2800–2400 Ma (2600 Ma and 2515 dominant peaks), 5% of zircons occurring at 300–250 Ma with (269 Ma dominant peak), and 5% of zircons occurring at 1800–1600 Ma (1678 Ma dominant peak).

A total of 80 detrital zircons from sample JF-39-R-1 were analysed, and concordant ages have a range of 1302–235 Ma with an age peak of 246 Ma (Fig. 5e,f).

110 detrital zircons from sample JF-43-R-1 were analysed, with the concordant ages have a range of 2590–242 Ma. The sample yielded five groups (Fig. 5g,h): 33% of zircons occurring at 500–400 Ma with (447 Ma dominant peak), 17% of zircons occurring at 2600–2400 Ma (2505 Ma dominant peak), 13% of zircons occurring at 300–242 Ma (244 Ma dominant peak), 12% of zircons occurring at 1940–1700 Ma (1851 Ma dominant peak), and 5% of zircons occurring at 1000–900 Ma (977 Ma dominant peak).

### Detrital zircon trace elements

**Figure 6:**
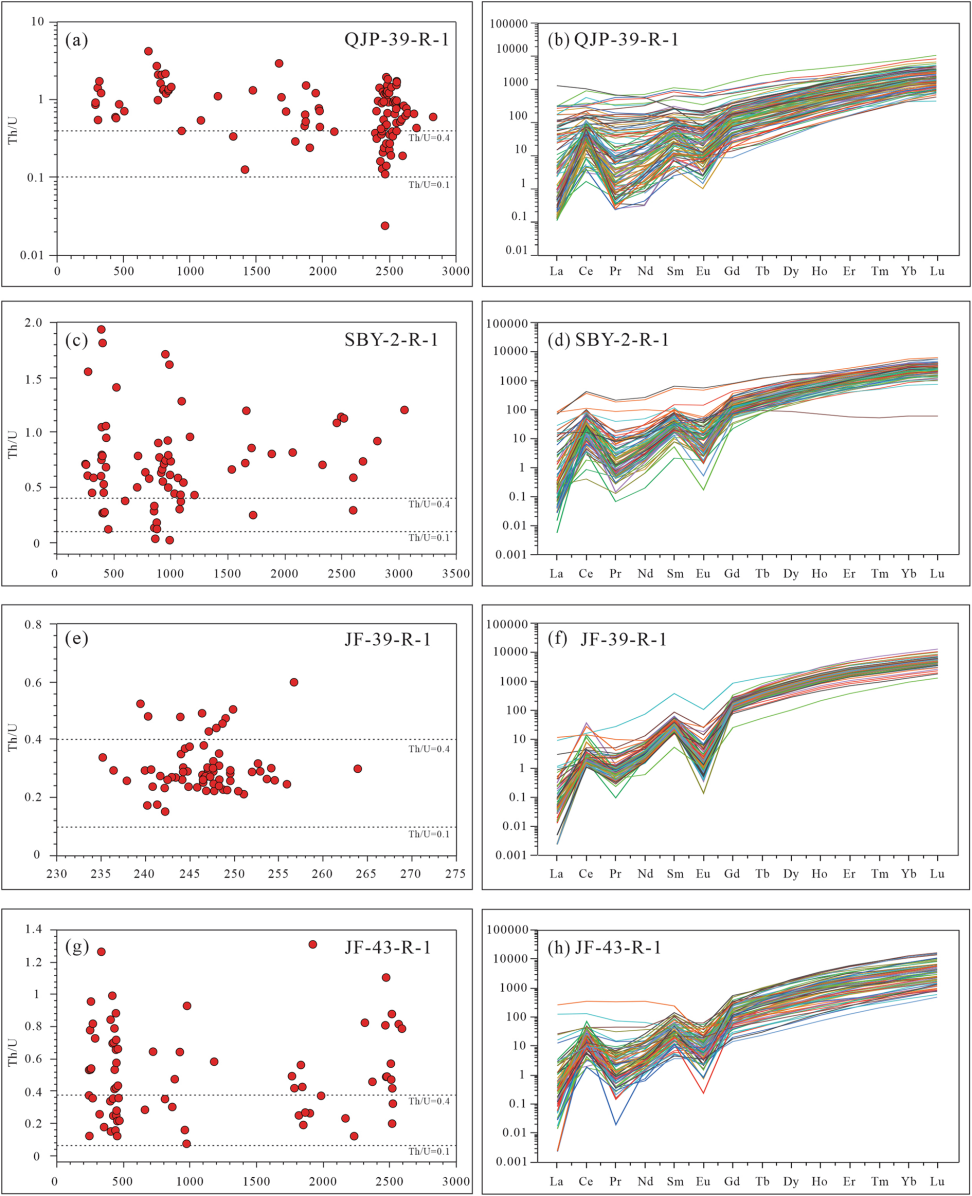
Th/U ratios and chondrite-normalized REE patterns of the detrital zircons. Normalization values of chondrite and primitive mantle are from^36^.

**Figure 7:**
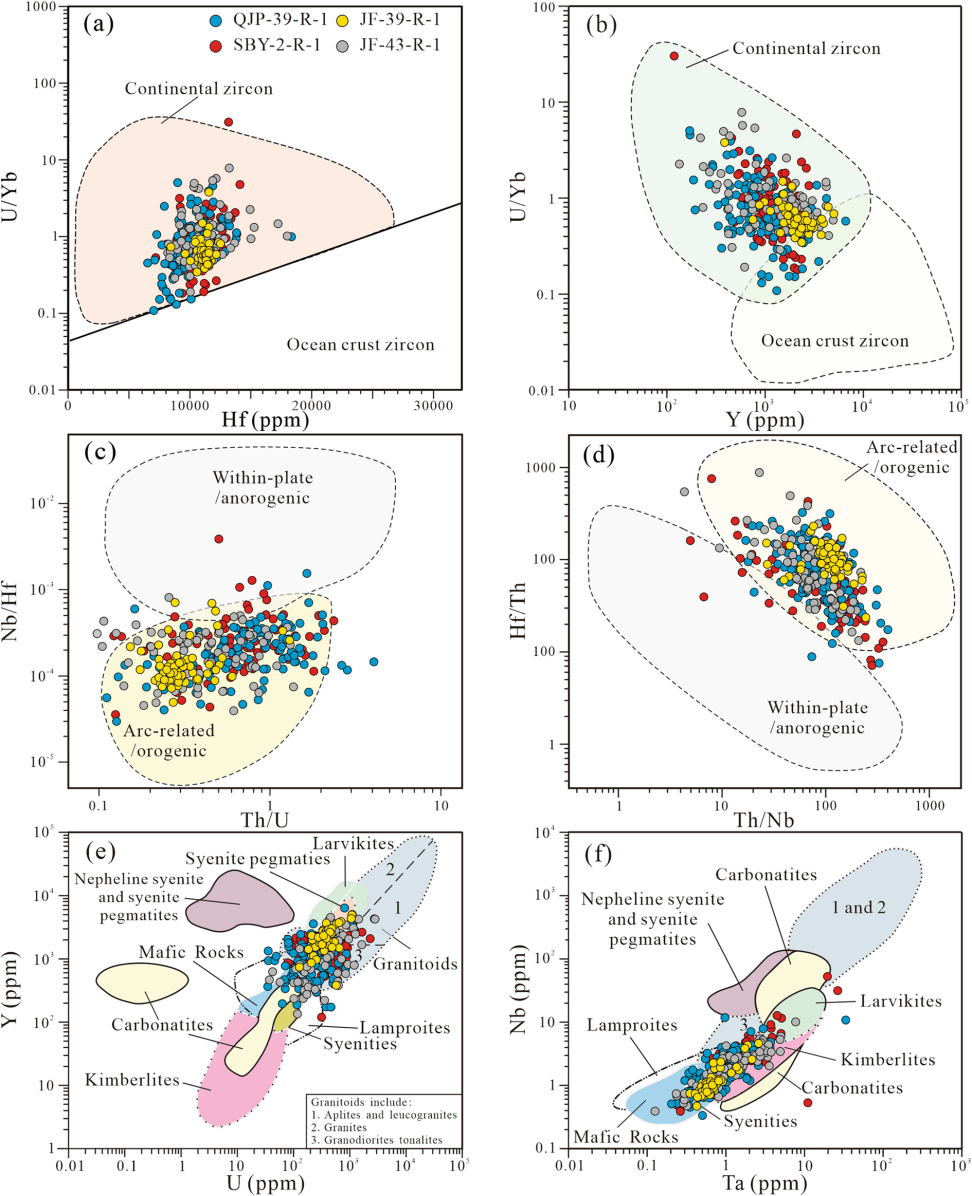
Geochemical discrimination diagrams for zircons. (**a**) Hf versus U/Yb diagram after^38^; (**b**) Y versus U/Yb diagram after^38^; (**c**) Th/U versus Nb/Hf diagram after^39^; (**d**) Th/Nb versus Hf/Th diagram after^39^; (**e**) U versus Y diagram after^40^; (**f**) Ta versus Nb diagram after^40^.

The detrital zircon trace element compositions are presented in Supplement Table 2. The Th/U ratios (moslyt > 0.1) of the detrital zircons from the samples (Fig. 6) reveal a magmatic origin. Most zircon grains show enrichment in LREE and flat HREE pattern with positive Ce anomalies and negative Eu anomalies (Fig. 6), which is consistent with a magmatic origin^37^. Zircons display dominantly continental signatures (Fig. 7a,b), and mainly are arc-related/orogenic origins (Fig. 7c,d). Most grains plot the field of the felsic rock (Fig. 7e,f).

### Detrital zircon Hf isotopes

**Figure 8:**
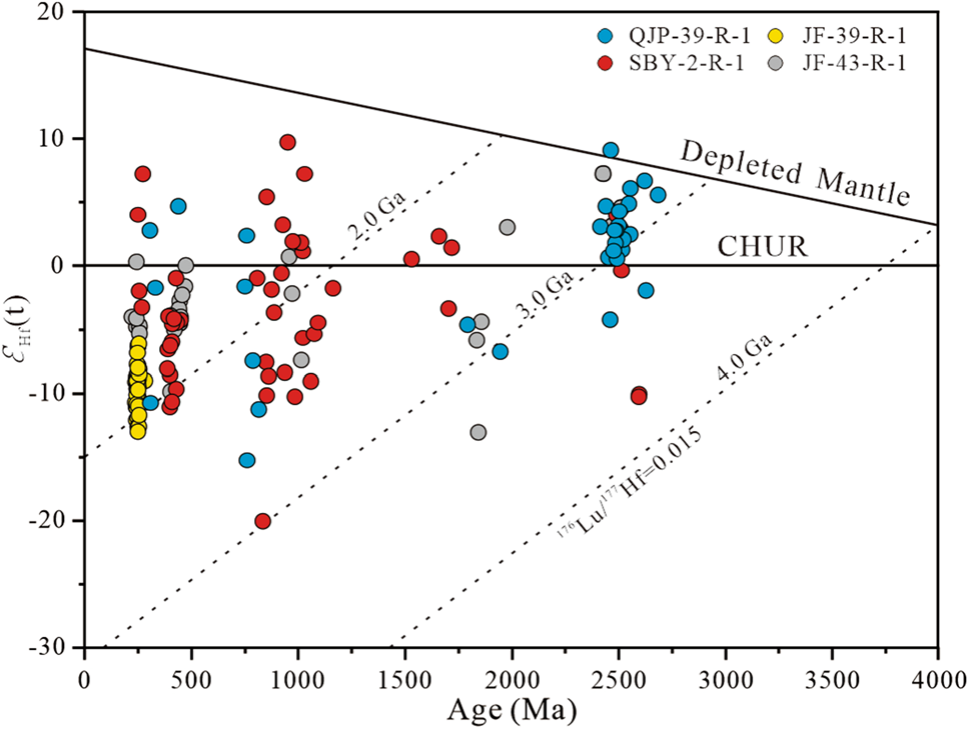
Hf isotopic compositions of the detrital zircons from the samples.

A total of 151 detrital zircons with concordant ages were further analysed for Lu‒Hf isotopes: 31 zircons for sample QJP-39-R-1, 49 zircons for sample SBY-2-R-1, 40 zircons for sample JF-39-R-1, and 31 zircons for sample JF-43-R-1 (Fig. 8). The results are presented in Supplement Table 3.

Detrital zircons with ages of 2700–2400 Ma from sample QJP-39-R-1 have ^176^Hf/^177^Hf ratios ranging from 0.281094 to 0.281490, *ε*_Hf(t)_ values ranging from − 4.3 to 9.0, with most zircons yielding positive εHf(t), and two-stage model (T_DM2_) ages ranging from 3227 to 2416 Ma. Detrital zircons with ages of 340–280, 900–700, and 2000–1600 Ma have ^176^Hf/^177^Hf ratios ranging from 0.281094 to 0.282668, εHf(t) values ranging from − 15.3 to 2.7, and TDM2 ages ranging from 2986 to 1145 Ma.

Detrital zircons with ages of 2800–2400, 1800–1600 and 300–250 Ma from sample SBY-2-R-1 have ^176^Hf/^177^Hf ratios ranging from 0.281699 to 0.282485, *ε*_Hf(t)_ values ranging from − 20.1 to 9.7, with most zircons yielding negative εHf(t), and T_DM2_ ages ranging from 2967 to 1203 Ma. Detrital zircons with ages of 340–280, 900–700, and 2000–1600 Ma have ^176^Hf/^177^Hf ratios ranging from 0.280874 to 0.282811, εHf(t) values ranging from − 10.3 to 7.2, and TDM2 ages ranging from 3695 to 835 Ma.

Detrital zircons with U–Pb ages of 280–235 Ma from sample JF-39-R-1 have ^176^Hf/^177^Hf ratios ranging from 0.282256 to 0.282451, negative εHf(t) values ranging from − 13.0 to − 6.1. The TDM2 ages ranging from 1659 to 2090 Ma.

Detrital zircons with ages from sample JF-43-R-1 have ^176^Hf/^177^Hf ratios ranging from 0.281262 to 0.282635, and εHf(t) values ranging from − 13 to 9.7, with most zircons yielding negative εHf(t), and TDM2 ages ranging from 3292 to 1247 Ma.

## Discussion

### Sedimentary provenance

#### Provenance of the Feixianguan Formation

The detrital zircon age spectrum of the Early Triassic Feixianguan Formation is samilar to those of the cotemporary clastic sediments in the northwestern Sichuan Basin and Zigui Basin^12,41^, and all shows the major peaks at ~ 2500 Ma, ~ 1850 Ma and ~ 850 Ma. This age peaks are consistent with the episodes of magmatic activities in the South China Block^42,43^. The detrital zircons in the range of 2700–2400 Ma constitute a dominant age group, with εHf(t) values of − 4.3 to 9.0. The Neoarchean to early Palaeoproterozoic rocks are sporadically exposed in the Yangtze Block, which is a significant component of the basement of the Yangtze Block^44^, represented by the gneiss with ages of 2.8–2.5 Ga and εHf(t) values of − 9.7 to 5 in the Kongling Complex^45–48^ and granite with ages of 2.8–2.4 Ga and εHf(t) values of − 3.4 to 8.1 in the Yudongzi Complex^49–51^. The late Palaeoproterozoic detrital zircons (2000–1600 Ma) are a major age component with predominantly negative εHf(t) values. These zircons may derive from the South China Block or North China Block^44^. If this sample received materials from the North China Block, these materitals flowed through the Qinling orogenic belt. However, age spectrum does not appear the age peaks with Qinling Orogenic Belt affinity. Thus, the late Palaeoproterozoic detrital zircons can not derived from the North China Block. This age is consistent with the episode of crustal growth of the South China Block^52^. The rocks of this age are distributed in the Kongling Complex along the margins of the Yangtze Block and Wuyishan region in the northeastern Cathaysia Block^53–56^. The Neoproterozoic detrital zircons (900–700 Ma) are a major age component with εHf(t) values of − 15.3 to 2.3. These age matches temporally with the break-up of the Rodinia supercontinent^57^. Neoproterozoic rocks related to the break-up event are well developed in the South China^58^ and mostly occur on the western and southeastern margin of the Yangtze Block^44^. The εHf(t) values of the zircons from the Neoproterozoic magmatic rocks along the margins of the Yangtze Block are similar to those from the detrital zircons with ages of 900–700 Ma^59^. However, the rocks in this age population are rarely exposed in the North China Block^43,60^. Detrital zircons in the range of 340–280 Ma are a minor component, with a major age peak of 306 Ma. The coeval rock units are only distributed in the southeastern South China Block, such as: gneissic granite with a U–Pb age of 313 ± 4 Ma in northeastern Fujian Province^61^. Meanwhile, detrital zircons of this age have been widely reported in the underlying Late Permian clastic sediments in the Fujian Province^62^. Therefore, these zircons from sample QJP-39-R-1 are suggested to be mainly derived from the South China Block.

#### Provenance of the Jialingjiang Formation

The detrital zircons in the range of 1100–800 Ma constitute a dominant age group, with major age peaks at 979 Ma and 856 Ma and mostly negative εHf(t) values of − 20.1 to 9.8. These ages are corresponding to the Neoproterozoic and Grenvillian ages related to the amalgamation and break-up, respectively, of the Rodinia supercontinent^57,63^. Detrital zircons in the range of 1100–800 Ma are euhedral and angular, indicates a short-distance deposition. Thus, these zircons likely derived from the adjacent terranes. The Neoproterozoic magmatic rocks are widely distributed along the margin of the Yangtze Block, North Qinling Belt and South Qinling Belt. Grenville-age magmatic rocks are widely distributed in the North Qinling Belt with ages of 979–911 Ma^9,64–66^. The North Qinling Belt underwent structural uplift and denudation during the early Mesozoic^67^. The detrital zircons in the range of 450–300 Ma are a major age component, with a major age peak at 392 Ma and negative εHf(t) values. These ages are equivalent to the late Early Palaeozoic magmatism in the Qinling Orogenic Belt. Coeval rocks are distributed in the central part of the North Qinling Belt, with ages of 415–400 Ma and mainly negative εHf(t) values^68,69^. The detrital zircons in the range of 2800–2400 Ma account for a small portion of the total zircons, and mainly derived from the Neoarchean to early Palaeoproterozoic crystalline basement of the Yangtze Block. The detrital zircons in the range of 300–250 Ma and 1800–1600 Ma are minor components, and derived from the Qinling Orogenic Belt^70,71^ and the western margin of the Yangtze Block^72^. In addition, the Jialingjiang Formation in the Zigui Basin has a large number of ca. 247 Ma detrital zircons and were deemed to derive from the Qinling Orogenic Belt, and the Precambrian detrital zircons might derive from the South China Block^12^. Hence, the results indicate a mixed sediment source for the Jialingjiang Formation sample that the Qinling Orogenic Belt and South China Block were the major source areas.

#### Provenance of the Leikoupo Formation

The detrital zircon age spectrum of the Leikoupo Formation samples differs from those of the underlying Lower Triassic samples (Fig. 6). Our samples and those of the Badong Formation in the Zigui Basin^12^ exhibit similar features with major populations ranging from 2600–2400 Ma, 1950–1700 Ma, 1000–900 Ma, 500–400 Ma and 300–245 Ma. The detrital zircons in the range of 500–400 Ma constitute a dominant age group of sample JF-43-R-1, with an age peak of 447 Ma and negative εHf(t) values. The early Palaeozoic (450–420 Ma) magmatic activity occurred mainly in the North Qinling Belt, which was correlated with the northward subduction of the Shangdan Ocean^24,67^. Thus, the contemporaneous granitoids were well-preserved in the North Qinling Belt, and have negative εHf(t) values^21,68,73^. The detrital zircon age spectra of the Triassic sedimentary rocks in the basins around the Qinling Orogenic Belt have a prominent characteristic: if the age spectra have an age peak of 500–400 Ma, and they are accompanied by an age peak of 300–200 Ma^12^. The age spectra of sample JF-39-R-1 are characterised by unimodal and narrow with a peak at 246 Ma, and the age spectra of sample JF-43-R-1 yields an age peak of 244 Ma. These zircons from the samples have negative εHf(t) values. The predominant ca. 245 Ma ages corresponded to the subduction of the Mianlue Ocean^9^. The contemporaneous granites are well preserved in the western South Qinling Belt, and the ages range from 245–220 Ma with mainly negative εHf(t) values^17,74^. Hence, the Precambrian detrital zircons are a small composition of the total zircons, and yield three major age peaks at 2505, 1851 and 977 Ma. The Jiangnan old land underwent tectonic uplift in the South China Block during the Middle Triassic, which resisted the transport of clastic materials from the Cathaysia to Yangtze blocks^75^. In addition, the Neoproterozoic magmatic rocks also sporadicly distributed in the Qiangtang Block^76^. The Precambrian detrital zircons may derive from the Qinling Orogenic Belt, Qiangtang Block and Yangtze Block. In conclusion, the main source region of the zircons in the Leikoupo Formation samples might have been the Qinling Orogenic Belt, and the South China Block was a minor source region.

### Timing of the initial collision between the South China and North China blocks

**Figure 9:**
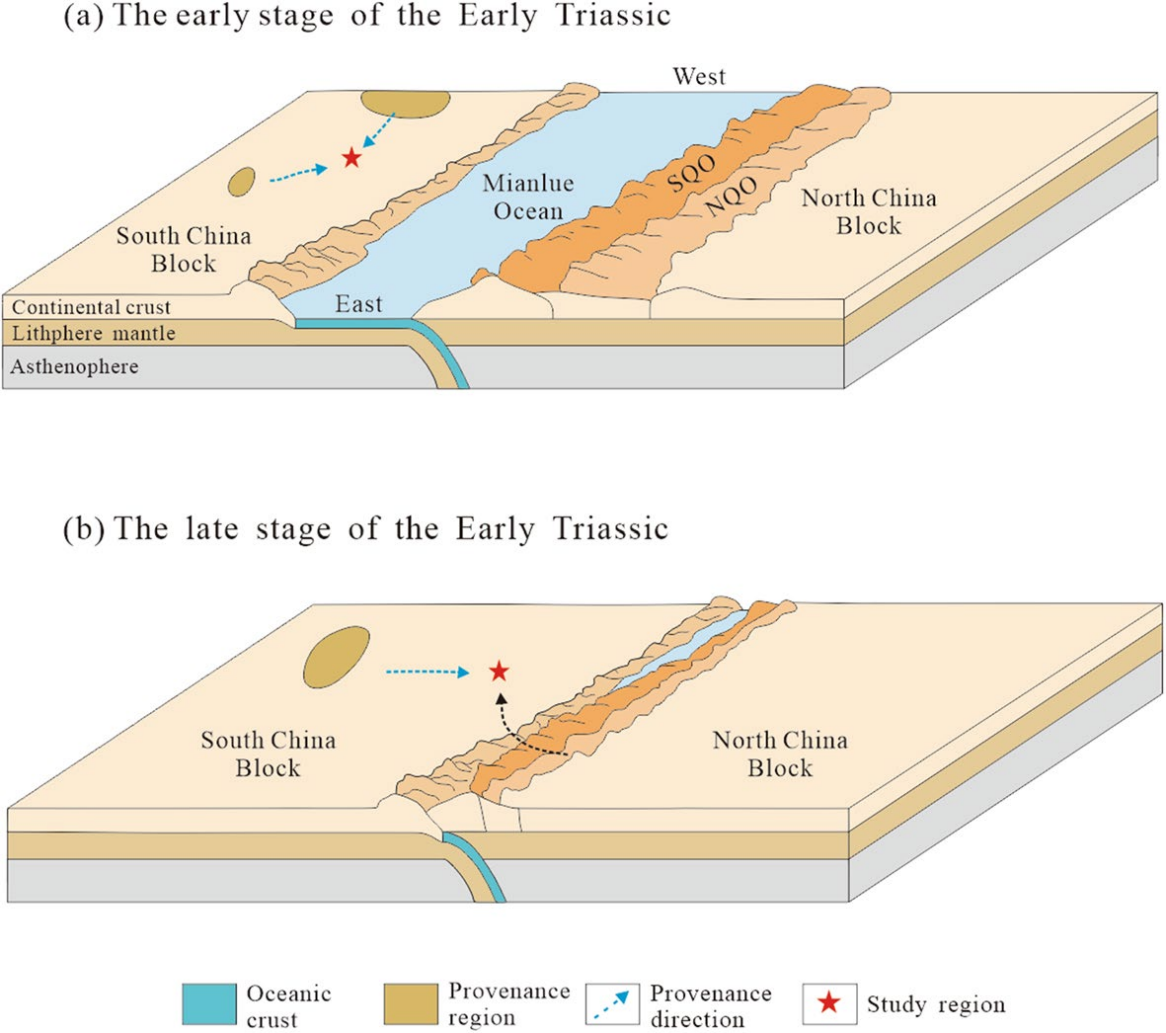
Schematic diagram depicting the initial collision between the South China and North China blocks^91^.

Due to the plate collisions do not generate direct geologic records, it is necessary to constrain the timing of the initial collision using the pre- or postcollision geological events^16,77^. The collision between the South China and North China blocks contributed to the formation of the Dabie-Hong’an-Tongbai-Qinling Orogenic Belt, which stretches more than 2,000 kilometres^78^. The earliest contact of this collision occurred in the Dabie Orogenic Belt in the eastern part of the whole collision belt, and the timing of the collision yield a younging trend from east to west^14,79^. The high-pressure eclogite with an age of 252 Ma in the Dabie Orogenic Belt records the subduction of oceanic crust before continental collision^80^. Palaeontological and palaeogeographical data indicate that the water depth increased from south to north at the northern margin of the Yangtze Block, which faced the Mianlue Ocean at the end of the Permian^81,82^. Palaeomagnetic studies have proposed that the South China and North China blocks collided in the Late Permian-Early Triassic^5,83,84^. Therefore, the initial collision of the two blocks in the Dabie orogenic belt did not occur earlier than the last phase of the Late Permian. Furthermore, a series of geological evidences indicate that the final convergence of the South China and North China blocks in the western Qinling Orogenic Belt occurred posterior to the end of the Late Triassic. The western Qinling Orogenic Belt was in a deep-water basin environment during the Early Triassic to the early Late Triassic, and preserves thick marine sedimentary successions^85,86^. In addition, the Late (228–210 Ma) granites in syn-collision turning to post-collision were widly distributed in the western Qinling Orogenic Belt^24,87,88^. Finally, the Songpan-Ganzi marine residual basin is filled with a considerable thickness of Middle-Late Triassic flysch. These abundant continental sediments have been deemed to derive from the Qinling-Dabie Orogenic Belt^89,90^. Hence, a channel should exist in the interzone between the South China and North China blocks, which can transport much debris from the orogenic belt into the Songpan-Ganzi residual basin. Before the initial collision, the South China and North China blocks were separated by the Mianlue Ocean, and the Sichuan Basin only possibly received the source materials provided by terranes in the South China Block (Fig. 9a). After the collision, the Qinling Orogenic Belt was uplifted and underwent long-term eolation and denudation, and formed massive detritus. The Sichuan Basin, as a sedimentary region, is adjacent to the Qinling Orogenic Belt, and began to receive this detritus. The earliest timing of the detritus reaching the Qinling Orogenic Belt can precisely constrain the upper limit of the timing of the initial collision. The provenance analysis suggests that the continuous deposits of the Feixianguan, Jialingjiang and Leikoupo formations have completely different material sources. The Feixianguan Formation may be derived from the South China Block. The occurrence of ~ 392 Ma detrital zircons with Qinling Orogenic Belt affinity in the Jialingjiang Formation indicates that the detritus from the Qinling Orogenic Belt first appeared in the Triassic successions in the study region. This is in accord with published data from the Zigui and Dangyang basins showing the first appearance of detritus from the Qinling Orogenic Belt happening in the Jialingjiang Formation^12^. The detritus from the Qinling Orogenic Belt in the Middle Triassic Leikoupo Formation is further increased and is the main source. Hence, provenance change suggests that the initial collision between the South China and North China blocks in the eastern Qinling Orogenic Belt occurred in the Early Triassic (Fig. 9b).

The tectonic evolution characteristics and transition from ocean to land in the northern part of the Middle-Upper Yangtze Block further support the Early Triassic initial collision between the South China and North China blocks. The timing of the cessation of marine deposition can be used to constrain the upper limit for the collision timing^77^. The Middle Triassic Leikoupo Formation and contemporaneous strata in the northern part of the Middle-Upper Yangtze Block are a set of marine carbonate rocks that are widely distributed, and the overlying Upper Triassic is a set of typical terrestrial clastic deposits^81^. Therefore, the Middle Triassic Leikoupo Formation and contemporaneous strata can represent the youngest marine strata in the northern part of the Middle-Upper Yangtze Block during the early Mesozoic. However, the northern part of the Middle-Upper Yangtze Block, including the Sichuan Basin, is the shallow water sedimentary region in the passive continental margin. The cessation of marine sedimentation only represents the timing of the seawater retreat from the Middle-Upper Yangtze Block, and is not the timing of the closing of the eastern Qinling Ocean. Hence, the youngest marine strata in the northern part of the Middle-Upper Yangtze Block may be much later than the collision timing^1^. In conclusion, the youngest marine strata suggest that the initial collision between the South China and North China blocks in the eastern Qinling Orogenic Belt may have occurred earlier than the Middle Triassic. The Triassic succession in the Michangshan region in the northern Sichuan Basin developed a regional unconformity in east–west trending, which is parallel to the Mianlue suture zone. This is construed as tectonic records related to the point contact collision between the South China and North China blocks in the eastern Qinling Orogenic Belt^92,93^. The palaeontological characteristics in the South Qinling Belt also support the hypothesis that block collisions occurred in the Early Triassic. The similarity between bivalve species in the Zhen’an region in the South Qinling Belt and the South China Block is as high as 87% during the early Early Triassic. Meanwhile, the similarity is low between the Zhen'an region and the southern margin of the North China Block at the early Early Triassic, and this value significantly increased at the late Early Triassic^94^. The significant increase in similarity at the late Early Triassic may be related to the migration of bivalves from the South Qinling Belt to the southern margin of the North China Block, which indicates that collision between the South China and North China blocks had already occurred at the late Early Triassic. The different geological perspectives suggest that the initial collision between the South China and North China blocks in the eastern Qinling Orogenic Belt occurred in the Early Triassic.

## Conclusions

Detrital zircon U–Pb geochronology, trace element and Hf isotope analyses were performed on the Early-Middle Triassic samples from the eastern Sichuan Basin, northwestern South China. Integrated with the previous research results, the following conclusions in this study could be obtained:

1. The detrital zircons from the Feixianguan Formation may derive from the South China Block. The zircons from the Jialingjiang Formation have a mixed source from the South China Block and Qinling Orogenic Belt. The zircons from the Leikoupo Formation may mainly derive from the Qinling Orogenic Belt.
2. The abrupt provenance change in detrital zircons from the Early-Middle Triassic successions was most likely a result of the collision between the South China and North China blocks. Thus, we suggest that the initial collision between the South China and North China blocks in the eastern Qinling Orogenic Belt occurred in the Early Triassic.

### Supplementary Information

Supplementary Information.

## Data Availability

All data generated or analysed during this study are included in the supplementary information files.
