# Supplementary material for: The timing of the initial collision between the South and North China blocks constraining from the sediments in the eastern Sichuan Basin
Source: Sci Rep. 2023 Dec 16;13:22378. doi: 10.1038/s41598-023-49498-z (PMC10725510; doi:10.1038/s41598-023-49498-z)
Supplement: Supplementary file 1 — Supplementary Information. [file 41598_2023_49498_MOESM1_ESM.docx]

Supplementary Table 1. Detrital zircon U–Pb isotopic compositions and ages of the sedimentary rocks from the Early-Middle Triassic in the study region.

| Suit  number | Content  (×10-6) | | Th/U | Corrected Ratios | | | | | | Corrected Ages (Ma) | | | | | | Conco-  rdance |
| --- | --- | --- | --- | --- | --- | --- | --- | --- | --- | --- | --- | --- | --- | --- | --- | --- |
|  | Th | U |  | 206Pb/238U | | 207Pb/235U | | 207Pb/206Pb | | 206Pb/238U | | 207Pb/235U | | 207Pb/206Pb | |  |
|  |  |  |  | Value | 1σ | Value | 1σ | Value | 1σ | Value | 1σ | Value | 1σ | Value | 1σ |  |
| QJP-39-R-1 |  |  |  |  |  |  |  |  |  |  |  |  |  |  |  |  |
| QJP-39-R-1-01 | 317.3 | 257.7 | 1.231 | 0.4300 | 0.0117 | 9.5379 | 0.2543 | 0.1617 | 0.0025 | 2305.5 | 52.8 | 2391.2 | 24.5 | 2473.8 | 26.2 | 96 |
| QJP-39-R-1-02 | 208.8 | 396.5 | 0.526 | 0.3036 | 0.0041 | 4.7836 | 0.0872 | 0.1143 | 0.0016 | 1709.4 | 20.2 | 1782.0 | 15.3 | 1869.4 | 25.9 | 95 |
| QJP-39-R-1-03 | 270.9 | 1119.6 | 0.242 | 0.3858 | 0.0101 | 8.5488 | 0.2614 | 0.1603 | 0.0020 | 2103.4 | 47.1 | 2291.1 | 27.8 | 2458.9 | 20.5 | 91 |
| QJP-39-R-1-04 | 366.2 | 616.3 | 0.594 | 0.1408 | 0.0025 | 1.9289 | 0.0504 | 0.1005 | 0.0038 | 849.2 | 14.2 | 1091.1 | 17.5 | 1633.0 | 70.2 | 75 |
| QJP-39-R-1-05 | 55.6 | 99.7 | 0.557 | 0.3764 | 0.0141 | 8.3402 | 0.3690 | 0.1599 | 0.0029 | 2059.3 | 66.1 | 2268.7 | 40.1 | 2455.2 | 30.2 | 90 |
| QJP-39-R-1-06 | 113.3 | 159.8 | 0.709 | 0.3041 | 0.0035 | 4.4213 | 0.0943 | 0.1054 | 0.0021 | 1711.7 | 17.2 | 1716.3 | 17.7 | 1721.9 | 36.7 | 99 |
| QJP-39-R-1-07 | 1081.7 | 999.3 | 1.082 | 0.0347 | 0.0017 | 0.3620 | 0.0182 | 0.0771 | 0.0025 | 219.6 | 10.3 | 313.7 | 13.6 | 1124.1 | 64.8 | 64 |
| QJP-39-R-1-08 | 281.0 | 133.1 | 2.111 | 0.1252 | 0.0017 | 1.1002 | 0.0389 | 0.0638 | 0.0022 | 760.3 | 9.8 | 753.5 | 18.8 | 744.5 | 72.2 | 99 |
| QJP-39-R-1-09 | 868.7 | 512.4 | 1.695 | 0.4157 | 0.0124 | 9.7296 | 0.3156 | 0.1696 | 0.0027 | 2240.9 | 56.6 | 2409.5 | 29.9 | 2554.0 | 26.2 | 92 |
| QJP-39-R-1-10 | 622.2 | 897.2 | 0.694 | 0.0411 | 0.0006 | 0.3466 | 0.0110 | 0.0612 | 0.0018 | 259.4 | 4.0 | 302.2 | 8.3 | 655.6 | 63.0 | 84 |
| QJP-39-R-1-11 | 282.3 | 436.7 | 0.646 | 0.3097 | 0.0041 | 4.8770 | 0.1111 | 0.1141 | 0.0022 | 1739.3 | 20.3 | 1798.3 | 19.2 | 1865.1 | 35.2 | 96 |
| QJP-39-R-1-12 | 198.5 | 585.3 | 0.339 | 0.2210 | 0.0032 | 2.6077 | 0.0787 | 0.0854 | 0.0022 | 1287.0 | 16.9 | 1302.8 | 22.2 | 1325.0 | 48.6 | 98 |
| QJP-39-R-1-13 | 821.1 | 472.3 | 1.739 | 0.0505 | 0.0007 | 0.3829 | 0.0178 | 0.0552 | 0.0027 | 317.7 | 4.4 | 329.1 | 13.1 | 420.4 | 109.2 | 96 |
| QJP-39-R-1-14 | 164.3 | 301.2 | 0.545 | 0.1920 | 0.0045 | 1.9944 | 0.0885 | 0.0755 | 0.0028 | 1132.2 | 24.4 | 1113.6 | 30.0 | 1081.2 | 75.6 | 98 |
| QJP-39-R-1-15 | 483.1 | 395.3 | 1.222 | 0.3038 | 0.0061 | 4.9756 | 0.1393 | 0.1186 | 0.0022 | 1710.3 | 30.2 | 1815.2 | 23.7 | 1944.5 | 33.3 | 94 |
| QJP-39-R-1-16 | 248.8 | 346.9 | 0.717 | 0.5179 | 0.0174 | 11.0499 | 0.4310 | 0.1551 | 0.0035 | 2690.3 | 73.7 | 2527.3 | 36.3 | 2402.2 | 37.8 | 93 |
| QJP-39-R-1-17 | 193.3 | 1188.4 | 0.163 | 0.4291 | 0.0090 | 9.3404 | 0.2512 | 0.1578 | 0.0028 | 2301.6 | 40.5 | 2372.0 | 24.7 | 2432.4 | 30.6 | 96 |
| QJP-39-R-1-18 | 110.5 | 71.1 | 1.554 | 0.5113 | 0.0091 | 11.9266 | 0.3695 | 0.1694 | 0.0046 | 2662.2 | 38.9 | 2598.7 | 29.0 | 2551.5 | 46.8 | 97 |
| QJP-39-R-1-19 | 175.3 | 313.6 | 0.559 | 0.0407 | 0.0011 | 0.3634 | 0.0331 | 0.0654 | 0.0059 | 257.2 | 7.0 | 314.7 | 24.6 | 787.0 | 188.9 | 79 |
| QJP-39-R-1-20 | 9.0 | 375.5 | 0.024 | 0.4213 | 0.0053 | 9.3392 | 0.2154 | 0.1610 | 0.0036 | 2266.3 | 24.0 | 2371.9 | 21.2 | 2466.4 | 39.0 | 95 |
| QJP-39-R-1-21 | 332.5 | 271.3 | 1.226 | 0.0527 | 0.0017 | 0.3657 | 0.0395 | 0.0536 | 0.0063 | 331.0 | 10.6 | 316.5 | 29.4 | 353.8 | 273.1 | 95 |
| QJP-39-R-1-22 | 64.4 | 495.7 | 0.130 | 0.5164 | 0.0090 | 11.2734 | 0.2683 | 0.1589 | 0.0035 | 2683.7 | 38.3 | 2546.0 | 22.2 | 2444.1 | 37.3 | 94 |
| QJP-39-R-1-23 | 524.6 | 202.1 | 2.595 | 0.0392 | 0.0016 | 0.3615 | 0.0347 | 0.0713 | 0.0071 | 248.1 | 9.6 | 313.3 | 25.9 | 964.8 | 210.2 | 76 |
| QJP-39-R-1-24 | 480.4 | 329.7 | 1.457 | 0.4942 | 0.0131 | 11.2548 | 0.3855 | 0.1657 | 0.0042 | 2588.9 | 56.6 | 2544.5 | 32.0 | 2514.5 | 47.2 | 98 |
| QJP-39-R-1-25 | 1390.9 | 1353.4 | 1.028 | 0.2767 | 0.0242 | 5.2008 | 0.4590 | 0.1375 | 0.0030 | 1574.5 | 122.2 | 1852.7 | 75.3 | 2196.0 | 38.0 | 83 |
| QJP-39-R-1-26 | 136.6 | 349.2 | 0.391 | 0.4060 | 0.0120 | 7.2035 | 0.2748 | 0.1290 | 0.0036 | 2196.8 | 55.2 | 2136.9 | 34.0 | 2084.9 | 49.1 | 97 |
| QJP-39-R-1-27 | 267.6 | 427.5 | 0.626 | 0.5878 | 0.0145 | 14.2504 | 0.4151 | 0.1764 | 0.0036 | 2980.6 | 59.0 | 2766.5 | 27.6 | 2620.4 | 34.4 | 92 |
| QJP-39-R-1-28 | 75.5 | 111.7 | 0.676 | 0.5914 | 0.0119 | 14.5471 | 0.6492 | 0.1781 | 0.0068 | 2995.0 | 48.4 | 2786.1 | 42.4 | 2634.9 | 64.4 | 92 |
| QJP-39-R-1-29 | 355.0 | 404.3 | 0.878 | 0.0751 | 0.0022 | 0.6191 | 0.0462 | 0.0590 | 0.0042 | 466.9 | 13.2 | 489.3 | 29.0 | 568.6 | 158.3 | 95 |
| QJP-39-R-1-30 | 1237.6 | 801.9 | 1.543 | 0.4678 | 0.0375 | 10.6188 | 0.9395 | 0.1626 | 0.0050 | 2473.9 | 164.6 | 2490.4 | 82.3 | 2483.0 | 51.4 | 99 |
| QJP-39-R-1-31 | 45.3 | 142.4 | 0.318 | 0.4636 | 0.0138 | 9.9842 | 0.4472 | 0.1551 | 0.0043 | 2455.4 | 60.7 | 2433.3 | 41.4 | 2403.4 | 47.2 | 99 |
| QJP-39-R-1-32 | 200.0 | 394.1 | 0.508 | 0.5144 | 0.0086 | 12.0209 | 0.3186 | 0.1694 | 0.0036 | 2675.5 | 36.5 | 2606.0 | 24.9 | 2551.5 | 34.7 | 97 |
| QJP-39-R-1-33 | 96.6 | 215.6 | 0.448 | 0.4313 | 0.0076 | 7.1594 | 0.2154 | 0.1212 | 0.0038 | 2311.5 | 34.3 | 2131.5 | 26.8 | 1975.9 | 56.5 | 91 |
| QJP-39-R-1-34 | 150.0 | 153.4 | 0.978 | 0.5187 | 0.0107 | 12.1057 | 0.4286 | 0.1694 | 0.0052 | 2693.7 | 45.5 | 2612.6 | 33.2 | 2553.7 | 50.2 | 96 |
| QJP-39-R-1-35 | 371.0 | 450.9 | 0.823 | 0.0610 | 0.0015 | 0.5717 | 0.0333 | 0.0686 | 0.0040 | 381.8 | 9.0 | 459.1 | 21.5 | 887.0 | 122.2 | 81 |
| QJP-39-R-1-36 | 196.5 | 494.3 | 0.398 | 0.5042 | 0.0139 | 11.7235 | 0.3514 | 0.1695 | 0.0037 | 2631.9 | 59.5 | 2582.6 | 28.1 | 2552.8 | 37.5 | 98 |
| QJP-39-R-1-37 | 115.7 | 266.6 | 0.434 | 0.0470 | 0.0016 | 0.4234 | 0.0348 | 0.0663 | 0.0047 | 296.2 | 9.8 | 358.4 | 24.8 | 816.7 | 148.9 | 80 |
| QJP-39-R-1-38 | 161.1 | 162.1 | 0.994 | 0.1248 | 0.0031 | 1.1103 | 0.0911 | 0.0649 | 0.0052 | 758.3 | 18.0 | 758.3 | 43.9 | 772.2 | 168.5 | 99 |
| QJP-39-R-1-39 | 61.0 | 294.3 | 0.207 | 0.5921 | 0.0126 | 13.0713 | 0.4500 | 0.1599 | 0.0043 | 2997.8 | 51.2 | 2684.8 | 32.5 | 2454.0 | 44.9 | 88 |
| QJP-39-R-1-40 | 139.9 | 195.5 | 0.716 | 0.0816 | 0.0020 | 0.7267 | 0.0638 | 0.0659 | 0.0059 | 505.9 | 11.9 | 554.6 | 37.6 | 1200.0 | 191.7 | 90 |
| QJP-39-R-1-41 | 104.3 | 35.4 | 2.947 | 0.3066 | 0.0089 | 4.3566 | 0.2922 | 0.1025 | 0.0055 | 1723.8 | 43.8 | 1704.2 | 55.4 | 1669.4 | 98.6 | 98 |
| QJP-39-R-1-42 | 177.1 | 838.7 | 0.211 | 0.4985 | 0.0093 | 10.9679 | 0.2638 | 0.1595 | 0.0029 | 2607.4 | 40.0 | 2520.4 | 22.4 | 2450.3 | 30.4 | 96 |
| QJP-39-R-1-43 | 208.3 | 1002.4 | 0.208 | 0.3309 | 0.0122 | 7.2015 | 0.2744 | 0.1579 | 0.0029 | 1842.9 | 59.3 | 2136.7 | 34.0 | 2433.0 | 31.3 | 85 |
| QJP-39-R-1-44 | 138.4 | 175.5 | 0.788 | 0.0438 | 0.0014 | 0.3843 | 0.0312 | 0.0651 | 0.0052 | 276.6 | 8.9 | 330.2 | 22.9 | 788.9 | 163.9 | 82 |
| QJP-39-R-1-45 | 319.1 | 457.1 | 0.698 | 0.0404 | 0.0006 | 0.3918 | 0.0178 | 0.0699 | 0.0029 | 255.5 | 4.0 | 335.7 | 13.0 | 924.1 | 83.3 | 72 |
| QJP-39-R-1-46 | 67.0 | 70.7 | 0.947 | 0.4685 | 0.0091 | 10.3165 | 0.3370 | 0.1601 | 0.0050 | 2477.2 | 39.8 | 2463.6 | 30.2 | 2456.5 | 52.9 | 99 |
| QJP-39-R-1-47 | 115.4 | 70.6 | 1.634 | 0.1282 | 0.0033 | 1.1067 | 0.0922 | 0.0631 | 0.0050 | 777.6 | 18.7 | 756.6 | 44.5 | 722.2 | 170.3 | 97 |
| QJP-39-R-1-48 | 276.3 | 328.3 | 0.842 | 0.4527 | 0.0089 | 10.9209 | 0.2650 | 0.1751 | 0.0036 | 2407.2 | 39.6 | 2516.4 | 22.6 | 2606.5 | 34.3 | 95 |
| QJP-39-R-1-49 | 138.4 | 148.6 | 0.931 | 0.4813 | 0.0111 | 10.7940 | 0.3055 | 0.1634 | 0.0044 | 2533.1 | 48.4 | 2505.5 | 26.3 | 2491.1 | 40.0 | 98 |
| QJP-39-R-1-50 | 114.2 | 501.9 | 0.228 | 0.5712 | 0.0135 | 12.9627 | 0.4208 | 0.1639 | 0.0037 | 2912.7 | 55.5 | 2676.9 | 30.6 | 2498.2 | 70.8 | 91 |
| QJP-39-R-1-51 | 244.2 | 276.4 | 0.883 | 0.0436 | 0.0009 | 0.4676 | 0.0236 | 0.0804 | 0.0049 | 275.0 | 5.7 | 389.6 | 16.3 | 1207.1 | 120.4 | 65 |
| QJP-39-R-1-52 | 1755.8 | 1782.1 | 0.985 | 0.0228 | 0.0003 | 0.3947 | 0.0103 | 0.1262 | 0.0036 | 145.3 | 2.2 | 337.8 | 7.5 | 2055.6 | 50.6 | 20 |
| QJP-39-R-1-53 | 878.2 | 1039.5 | 0.845 | 0.0389 | 0.0011 | 0.3981 | 0.0174 | 0.0752 | 0.0033 | 246.2 | 6.6 | 340.2 | 12.6 | 1075.9 | 88.9 | 67 |
| QJP-39-R-1-54 | 262.3 | 904.1 | 0.290 | 0.2537 | 0.0041 | 5.1274 | 0.1328 | 0.1463 | 0.0028 | 1457.5 | 20.9 | 1840.7 | 22.0 | 2302.8 | 33.0 | 76 |
| QJP-39-R-1-56 | 229.1 | 205.5 | 1.115 | 0.2284 | 0.0038 | 2.5287 | 0.1056 | 0.0805 | 0.0032 | 1326.2 | 19.7 | 1280.3 | 30.4 | 1209.3 | 77.8 | 96 |
| QJP-39-R-1-57 | 376.7 | 89.2 | 4.225 | 0.1124 | 0.0040 | 1.0644 | 0.1069 | 0.0672 | 0.0056 | 686.7 | 23.2 | 736.0 | 52.6 | 844.1 | 174.1 | 93 |
| QJP-39-R-1-58 | 222.0 | 336.5 | 0.660 | 0.5403 | 0.0094 | 12.5366 | 0.4391 | 0.1681 | 0.0047 | 2784.8 | 39.4 | 2645.5 | 32.9 | 2538.6 | 47.2 | 94 |
| QJP-39-R-1-59 | 224.1 | 515.1 | 0.435 | 0.5541 | 0.0243 | 12.7799 | 0.6894 | 0.1855 | 0.0197 | 2842.1 | 100.8 | 2663.6 | 50.8 | 2702.8 | 175.8 | 93 |
| QJP-39-R-1-60 | 500.8 | 648.5 | 0.772 | 0.0461 | 0.0014 | 0.4148 | 0.0269 | 0.0678 | 0.0047 | 290.8 | 8.6 | 352.3 | 19.3 | 861.1 | 144.1 | 80 |
| QJP-39-R-1-61 | 224.5 | 186.2 | 1.205 | 0.1355 | 0.0027 | 1.2621 | 0.0400 | 0.0677 | 0.0018 | 819.4 | 15.3 | 828.9 | 17.9 | 861.1 | 58.3 | 98 |
| QJP-39-R-1-62 | 166.1 | 859.6 | 0.193 | 0.4793 | 0.0119 | 10.9590 | 0.3879 | 0.1652 | 0.0035 | 2524.4 | 51.9 | 2519.7 | 32.9 | 2510.2 | 36.3 | 99 |
| QJP-39-R-1-63 | 142.0 | 92.6 | 1.534 | 0.3482 | 0.0072 | 5.4240 | 0.2445 | 0.1144 | 0.0058 | 1926.1 | 34.5 | 1888.7 | 38.7 | 1872.2 | 92.4 | 98 |
| QJP-39-R-1-64 | 109.4 | 62.9 | 1.739 | 0.4708 | 0.0084 | 10.9722 | 0.3684 | 0.1695 | 0.0055 | 2486.9 | 37.0 | 2520.8 | 31.3 | 2553.7 | 54.0 | 98 |
| QJP-39-R-1-65 | 129.7 | 345.9 | 0.375 | 0.3943 | 0.0152 | 8.3977 | 0.3759 | 0.1541 | 0.0035 | 2142.5 | 70.3 | 2274.9 | 40.6 | 2392.3 | 38.9 | 94 |
| QJP-39-R-1-66 | 73.5 | 184.1 | 0.399 | 0.1778 | 0.0044 | 1.7075 | 0.0581 | 0.0703 | 0.0025 | 1055.0 | 24.3 | 1011.3 | 21.8 | 936.1 | 74.1 | 95 |
| QJP-39-R-1-67 | 1031.4 | 872.2 | 1.182 | 0.0466 | 0.0015 | 0.4536 | 0.0204 | 0.0738 | 0.0047 | 293.9 | 9.2 | 379.8 | 14.2 | 1035.2 | 129.6 | 74 |
| QJP-39-R-1-68 | 310.4 | 336.7 | 0.922 | 0.0459 | 0.0010 | 0.3419 | 0.0185 | 0.0541 | 0.0028 | 289.2 | 6.0 | 298.6 | 14.0 | 376.0 | 123.1 | 96 |
| QJP-39-R-1-69 | 103.8 | 78.8 | 1.318 | 0.1387 | 0.0035 | 1.3600 | 0.1093 | 0.0725 | 0.0059 | 837.6 | 20.0 | 871.9 | 47.1 | 1011.1 | 165.3 | 95 |
| QJP-39-R-1-70 | 77.3 | 35.6 | 2.171 | 0.1347 | 0.0043 | 1.2368 | 0.1305 | 0.0693 | 0.0084 | 814.7 | 24.7 | 817.4 | 59.3 | 907.1 | 251.8 | 99 |
| QJP-39-R-1-71 | 44.2 | 182.1 | 0.243 | 0.3752 | 0.0065 | 5.9682 | 0.1810 | 0.1156 | 0.0035 | 2053.6 | 30.6 | 1971.2 | 26.4 | 1900.0 | 54.9 | 95 |
| QJP-39-R-1-72 | 111.4 | 284.2 | 0.392 | 0.5780 | 0.0097 | 13.2768 | 0.3424 | 0.1661 | 0.0035 | 2940.7 | 39.5 | 2699.5 | 24.4 | 2520.4 | 35.2 | 91 |
| QJP-39-R-1-73 | 295.9 | 223.2 | 1.326 | 0.2618 | 0.0045 | 3.3429 | 0.1279 | 0.0922 | 0.0030 | 1499.0 | 23.1 | 1491.1 | 29.9 | 1472.2 | 61.1 | 99 |
| QJP-39-R-1-74 | 36.7 | 289.1 | 0.127 | 0.1838 | 0.0027 | 2.2591 | 0.0496 | 0.0894 | 0.0021 | 1087.5 | 14.5 | 1199.6 | 15.5 | 1413.0 | 44.4 | 90 |
| QJP-39-R-1-75 | 106.3 | 114.7 | 0.926 | 0.4855 | 0.0090 | 11.1851 | 0.3509 | 0.1669 | 0.0042 | 2551.1 | 39.2 | 2538.7 | 29.3 | 2527.8 | 42.3 | 99 |
| QJP-39-R-1-76 | 74.9 | 135.9 | 0.551 | 0.0489 | 0.0015 | 0.3389 | 0.0361 | 0.0509 | 0.0051 | 307.8 | 8.9 | 296.4 | 27.4 | 235.3 | 218.5 | 96 |
| QJP-39-R-1-77 | 193.6 | 140.9 | 1.374 | 0.1326 | 0.0034 | 1.2605 | 0.0648 | 0.0693 | 0.0034 | 802.7 | 19.1 | 828.1 | 29.1 | 905.6 | 100.0 | 96 |
| QJP-39-R-1-78 | 285.5 | 681.0 | 0.419 | 0.3679 | 0.0133 | 8.3321 | 0.3282 | 0.1644 | 0.0026 | 2019.5 | 62.9 | 2267.8 | 35.7 | 2501.5 | 27.2 | 88 |
| QJP-39-R-1-79 | 36.0 | 46.1 | 0.780 | 0.4070 | 0.0085 | 6.7560 | 0.2535 | 0.1208 | 0.0040 | 2201.0 | 39.1 | 2080.0 | 33.2 | 1968.5 | 60.3 | 94 |
| QJP-39-R-1-80 | 115.0 | 118.7 | 0.969 | 0.4870 | 0.0124 | 10.3836 | 0.3673 | 0.1561 | 0.0049 | 2557.9 | 53.9 | 2469.6 | 32.8 | 2413.3 | 53.4 | 96 |
| QJP-39-R-1-81 | 548.6 | 1004.6 | 0.546 | 0.2416 | 0.0124 | 4.3516 | 0.2260 | 0.1315 | 0.0024 | 1395.2 | 64.2 | 1703.2 | 42.9 | 2118.2 | 31.5 | 80 |
| QJP-39-R-1-82 | 481.0 | 265.9 | 1.809 | 0.0377 | 0.0015 | 0.4053 | 0.0507 | 0.0734 | 0.0061 | 238.4 | 9.5 | 345.5 | 36.7 | 1025.6 | 165.7 | 63 |
| QJP-39-R-1-83 | 97.2 | 82.4 | 1.180 | 0.4884 | 0.0080 | 10.7619 | 0.3018 | 0.1606 | 0.0044 | 2563.8 | 34.6 | 2502.8 | 26.1 | 2462.0 | 46.3 | 97 |
| QJP-39-R-1-84 | 53.9 | 91.2 | 0.591 | 0.0395 | 0.0010 | 0.3554 | 0.0273 | 0.0669 | 0.0054 | 249.5 | 6.1 | 308.8 | 20.4 | 835.2 | 167.4 | 78 |
| QJP-39-R-1-85 | 106.9 | 177.6 | 0.602 | 0.0704 | 0.0015 | 0.5181 | 0.0384 | 0.0541 | 0.0041 | 438.8 | 9.2 | 423.9 | 25.7 | 376.0 | 202.8 | 96 |
| QJP-39-R-1-86 | 49.6 | 145.3 | 0.341 | 0.5112 | 0.0136 | 11.7436 | 0.4040 | 0.1665 | 0.0032 | 2661.7 | 58.0 | 2584.2 | 32.2 | 2524.1 | 32.1 | 97 |
| QJP-39-R-1-87 | 58.9 | 104.7 | 0.562 | 0.4881 | 0.0079 | 11.6275 | 0.3181 | 0.1732 | 0.0046 | 2562.6 | 34.3 | 2574.9 | 25.6 | 2590.7 | 43.1 | 99 |
| QJP-39-R-1-88 | 350.1 | 189.9 | 1.844 | 0.4326 | 0.0082 | 9.7374 | 0.2756 | 0.1629 | 0.0032 | 2317.5 | 36.9 | 2410.2 | 26.1 | 2487.0 | 31.9 | 96 |
| QJP-39-R-1-89 | 247.4 | 623.0 | 0.397 | 0.4558 | 0.0163 | 10.0149 | 0.4119 | 0.1589 | 0.0028 | 2420.9 | 72.2 | 2436.2 | 38.0 | 2443.5 | 29.6 | 99 |
| QJP-39-R-1-90 | 52.6 | 369.8 | 0.142 | 0.3924 | 0.0088 | 8.7616 | 0.2401 | 0.1618 | 0.0028 | 2133.8 | 41.0 | 2313.5 | 25.0 | 2475.9 | 29.6 | 91 |
| QJP-39-R-1-91 | 160.0 | 376.5 | 0.425 | 0.3863 | 0.0140 | 8.4282 | 0.3454 | 0.1579 | 0.0029 | 2105.5 | 65.3 | 2278.2 | 37.2 | 2435.2 | 31.2 | 92 |
| QJP-39-R-1-92 | 133.0 | 288.2 | 0.461 | 0.3674 | 0.0068 | 5.7315 | 0.1317 | 0.1137 | 0.0027 | 2017.0 | 32.3 | 1936.1 | 19.9 | 1861.1 | 42.3 | 95 |
| QJP-39-R-1-93 | 0.0 | 0.0 | 0.000 | 3.0730 | 0.9486 | 260.1792 | 182.1604 | 3.1253 | 1.2427 | 9053.3 | 1529.5 | 5650.8 | 875.6 | — | — | 53 |
| QJP-39-R-1-94 | 109.3 | 142.0 | 0.769 | 0.4896 | 0.0073 | 11.4470 | 0.2595 | 0.1691 | 0.0028 | 2569.0 | 31.7 | 2560.3 | 21.2 | 2550.0 | 28.1 | 99 |
| QJP-39-R-1-95 | 51.9 | 88.1 | 0.589 | 0.0726 | 0.0016 | 0.6726 | 0.0503 | 0.0682 | 0.0054 | 451.9 | 9.7 | 522.3 | 30.6 | 875.9 | 164.8 | 85 |
| QJP-39-R-1-96 | 129.0 | 99.8 | 1.293 | 0.4711 | 0.0076 | 10.5697 | 0.2777 | 0.1627 | 0.0039 | 2488.4 | 33.3 | 2486.1 | 24.4 | 2484.3 | 39.8 | 99 |
| QJP-39-R-1-97 | 497.7 | 237.1 | 2.100 | 0.1298 | 0.0023 | 1.1460 | 0.0478 | 0.0640 | 0.0025 | 786.8 | 13.3 | 775.4 | 22.6 | 742.6 | 86.1 | 98 |
| QJP-39-R-1-98 | 242.0 | 358.1 | 0.676 | 0.4108 | 0.0156 | 9.2287 | 0.3810 | 0.1628 | 0.0030 | 2218.4 | 71.4 | 2361.0 | 37.8 | 2484.9 | 31.5 | 93 |
| QJP-39-R-1-99 | 91.5 | 254.0 | 0.360 | 0.4849 | 0.0115 | 10.5531 | 0.2788 | 0.1583 | 0.0033 | 2548.7 | 49.9 | 2484.6 | 24.5 | 2438.9 | 34.9 | 97 |
| QJP-39-R-1-100 | 471.9 | 741.7 | 0.636 | 0.1714 | 0.0076 | 3.9568 | 0.2123 | 0.1667 | 0.0037 | 1020.0 | 41.7 | 1625.4 | 43.5 | 2524.4 | 37.3 | 54 |
| QJP-39-R-1-101 | 48.2 | 252.4 | 0.191 | 0.5314 | 0.0088 | 12.7595 | 0.2962 | 0.1740 | 0.0032 | 2747.2 | 37.2 | 2662.1 | 21.9 | 2598.2 | 31.0 | 96 |
| QJP-39-R-1-102 | 273.8 | 858.1 | 0.319 | 0.2137 | 0.0279 | 11.6034 | 3.0685 | 0.2634 | 0.0295 | 1248.6 | 148.0 | 2573.0 | 252.3 | 3277.8 | 177.2 | 30 |
| QJP-39-R-1-103 | 52.0 | 98.8 | 0.527 | 0.5173 | 0.0087 | 12.2516 | 0.2922 | 0.1724 | 0.0042 | 2687.5 | 37.1 | 2623.9 | 22.4 | 2581.2 | 40.4 | 97 |
| QJP-39-R-1-104 | 355.1 | 280.3 | 1.267 | 0.5256 | 0.0085 | 11.5571 | 0.2618 | 0.1593 | 0.0029 | 2722.8 | 36.0 | 2569.2 | 21.2 | 2450.0 | 31.0 | 94 |
| QJP-39-R-1-105 | 287.8 | 196.4 | 1.466 | 0.1425 | 0.0034 | 1.2375 | 0.0606 | 0.0633 | 0.0030 | 858.6 | 19.3 | 817.8 | 27.5 | 720.4 | 100.0 | 95 |
| QJP-39-R-1-106 | 1539.6 | 810.9 | 1.899 | 0.0392 | 0.0011 | 0.4647 | 0.0160 | 0.0878 | 0.0036 | 247.7 | 6.9 | 387.5 | 11.1 | 1377.5 | 77.8 | 55 |
| QJP-39-R-1-107 | 32.5 | 49.0 | 0.663 | 0.5352 | 0.0130 | 13.5313 | 0.5054 | 0.1832 | 0.0054 | 2763.1 | 54.7 | 2717.5 | 35.3 | 2683.3 | 49.2 | 98 |
| QJP-39-R-1-108 | 311.5 | 318.4 | 0.978 | 0.5006 | 0.0091 | 11.2390 | 0.3019 | 0.1626 | 0.0035 | 2616.5 | 39.3 | 2543.2 | 25.0 | 2483.6 | 36.9 | 97 |
| QJP-39-R-1-109 | 259.1 | 316.9 | 0.818 | 0.6800 | 0.0123 | 28.5983 | 0.7619 | 0.3043 | 0.0061 | 3344.5 | 47.4 | 3439.8 | 26.1 | 3492.3 | 31.0 | 97 |
| QJP-39-R-1-110 | 162.3 | 155.0 | 1.047 | 0.0615 | 0.0027 | 0.6451 | 0.1312 | 0.0770 | 0.0158 | 384.9 | 16.3 | 505.4 | 81.2 | 1120.4 | 420.8 | 72 |
| QJP-39-R-1-111 | 119.6 | 99.3 | 1.204 | 0.5053 | 0.0091 | 11.3205 | 0.3026 | 0.1624 | 0.0036 | 2636.6 | 39.0 | 2549.9 | 24.9 | 2481.2 | 37.7 | 96 |
| QJP-39-R-1-112 | 178.8 | 197.0 | 0.908 | 0.5332 | 0.0092 | 11.6889 | 0.2994 | 0.1587 | 0.0032 | 2754.8 | 38.9 | 2579.8 | 24.0 | 2442.3 | 34.6 | 93 |
| QJP-39-R-1-113 | 66.0 | 226.0 | 0.292 | 0.2752 | 0.0072 | 4.1711 | 0.2088 | 0.1095 | 0.0043 | 1567.3 | 36.4 | 1668.4 | 41.0 | 1791.7 | 70.4 | 93 |
| QJP-39-R-1-114 | 159.2 | 204.5 | 0.778 | 0.5020 | 0.0076 | 12.2766 | 0.2866 | 0.1772 | 0.0036 | 2622.5 | 32.4 | 2625.8 | 21.9 | 2626.9 | 32.9 | 99 |
| QJP-39-R-1-115 | 111.9 | 155.5 | 0.720 | 0.3408 | 0.0074 | 5.6947 | 0.2008 | 0.1212 | 0.0038 | 1890.4 | 35.4 | 1930.6 | 30.5 | 1973.8 | 56.0 | 97 |
| QJP-39-R-1-116 | 64.4 | 68.5 | 0.940 | 0.5057 | 0.0089 | 11.7658 | 0.3249 | 0.1687 | 0.0041 | 2638.2 | 38.1 | 2585.9 | 25.8 | 2546.3 | 40.7 | 98 |
| QJP-39-R-1-117 | 198.7 | 149.2 | 1.332 | 0.1318 | 0.0031 | 1.2357 | 0.0511 | 0.0688 | 0.0031 | 798.0 | 17.4 | 817.0 | 23.2 | 892.3 | 91.5 | 97 |
| QJP-39-R-1-118 | 287.4 | 209.1 | 1.374 | 0.1390 | 0.0025 | 1.4888 | 0.1283 | 0.0764 | 0.0058 | 839.0 | 14.4 | 925.8 | 52.4 | 1105.6 | 151.9 | 90 |
| QJP-39-R-1-119 | 59.7 | 98.9 | 0.604 | 0.5545 | 0.0085 | 15.3115 | 0.3768 | 0.2002 | 0.0049 | 2843.9 | 35.1 | 2834.8 | 23.5 | 2827.5 | 39.5 | 99 |
| QJP-39-R-1-120 | 574.8 | 307.3 | 1.871 | 0.2605 | 0.0052 | 4.1856 | 0.1188 | 0.1164 | 0.0027 | 1492.4 | 26.8 | 1671.2 | 23.3 | 1902.2 | 41.2 | 88 |
| QJP-39-R-1-121 | 84.6 | 145.3 | 0.583 | 0.0712 | 0.0015 | 0.4958 | 0.0387 | 0.0511 | 0.0041 | 443.3 | 9.1 | 408.9 | 26.2 | 255.6 | 182.4 | 91 |
| QJP-39-R-1-122 | 65.9 | 28.7 | 2.300 | 0.1169 | 0.0038 | 1.4084 | 0.1113 | 0.0927 | 0.0085 | 712.7 | 22.2 | 892.5 | 47.0 | 1483.3 | 178.7 | 77 |
| QJP-39-R-1-123 | 172.8 | 140.7 | 1.228 | 0.5001 | 0.0069 | 11.2828 | 0.2674 | 0.1639 | 0.0037 | 2614.4 | 29.7 | 2546.8 | 22.1 | 2496.0 | 71.5 | 97 |
| QJP-39-R-1-124 | 30.4 | 34.8 | 0.875 | 0.5165 | 0.0099 | 11.9389 | 0.4552 | 0.1688 | 0.0066 | 2684.2 | 42.0 | 2599.6 | 35.7 | 2546.0 | 65.4 | 96 |
| QJP-39-R-1-125 | 83.9 | 235.0 | 0.357 | 0.4316 | 0.0064 | 9.8028 | 0.3133 | 0.1644 | 0.0043 | 2313.0 | 28.8 | 2416.4 | 29.5 | 2501.5 | 44.4 | 95 |
| QJP-39-R-1-126 | 51.3 | 461.4 | 0.111 | 0.5285 | 0.0092 | 11.6861 | 0.2747 | 0.1607 | 0.0031 | 2735.0 | 38.8 | 2579.6 | 22.0 | 2462.7 | 31.6 | 94 |
| QJP-39-R-1-127 | 216.4 | 200.2 | 1.081 | 0.2600 | 0.0121 | 3.6907 | 0.1955 | 0.1034 | 0.0036 | 1490.1 | 61.7 | 1569.4 | 42.3 | 1687.0 | 64.8 | 94 |
| QJP-39-R-1-128 | 1125.4 | 686.9 | 1.638 | 0.0432 | 0.0011 | 0.4406 | 0.0332 | 0.0734 | 0.0044 | 272.4 | 7.0 | 370.7 | 23.4 | 1033.3 | 121.1 | 69 |
| QJP-39-R-1-129 | 279.0 | 195.1 | 1.430 | 0.4237 | 0.0123 | 9.1847 | 0.3571 | 0.1571 | 0.0035 | 2277.0 | 55.7 | 2356.6 | 35.6 | 2424.4 | 37.5 | 96 |
| QJP-39-R-1-130 | 59.2 | 123.4 | 0.480 | 0.4718 | 0.0093 | 10.4373 | 0.3282 | 0.1611 | 0.0044 | 2491.4 | 40.6 | 2474.4 | 29.1 | 2477.8 | 50.8 | 99 |
| QJP-39-R-1-131 | 159.8 | 81.5 | 1.962 | 0.5239 | 0.0088 | 11.6977 | 0.3547 | 0.1619 | 0.0039 | 2715.8 | 37.3 | 2580.5 | 28.4 | 2475.6 | 40.7 | 94 |
| QJP-39-R-1-132 | 71.2 | 259.0 | 0.275 | 0.5211 | 0.0115 | 11.8052 | 0.3651 | 0.1643 | 0.0037 | 2704.0 | 48.8 | 2589.1 | 29.0 | 2501.9 | 38.1 | 95 |
| QJP-39-R-1-133 | 394.8 | 277.0 | 1.425 | 0.0485 | 0.0007 | 0.3300 | 0.0231 | 0.0497 | 0.0035 | 305.3 | 4.6 | 289.6 | 17.6 | 189.0 | 167.6 | 94 |
| QJP-39-R-1-134 | 109.8 | 241.6 | 0.454 | 0.4816 | 0.0076 | 10.6648 | 0.2599 | 0.1608 | 0.0036 | 2534.4 | 33.2 | 2494.4 | 22.6 | 2464.5 | 38.0 | 98 |
| QJP-39-R-1-135 | 395.7 | 311.4 | 1.271 | 0.4841 | 0.0074 | 10.8035 | 0.2166 | 0.1621 | 0.0030 | 2545.3 | 32.3 | 2506.4 | 18.6 | 2479.6 | 31.5 | 98 |
| QJP-39-R-1-136 | 149.1 | 150.8 | 0.988 | 0.4615 | 0.0103 | 10.7165 | 0.3286 | 0.1680 | 0.0033 | 2446.3 | 45.3 | 2498.9 | 28.5 | 2538.6 | 33.3 | 97 |
| QJP-39-R-1-137 | 129.1 | 470.0 | 0.275 | 0.4666 | 0.0069 | 10.4226 | 0.2281 | 0.1618 | 0.0029 | 2468.8 | 30.2 | 2473.1 | 20.3 | 2475.9 | 30.6 | 99 |
| QJP-39-R-1-138 | 431.2 | 497.3 | 0.867 | 0.0458 | 0.0012 | 0.3647 | 0.0183 | 0.0579 | 0.0026 | 288.7 | 7.1 | 315.7 | 13.6 | 524.1 | 106.5 | 91 |
| QJP-39-R-1-139 | 221.0 | 81.1 | 2.725 | 0.1234 | 0.0023 | 1.0408 | 0.0650 | 0.0623 | 0.0043 | 750.2 | 13.0 | 724.3 | 32.4 | 684.9 | 150.9 | 96 |
| QJP-39-R-1-140 | 584.3 | 806.5 | 0.725 | 0.0363 | 0.0010 | 0.3028 | 0.0132 | 0.0615 | 0.0027 | 229.7 | 6.2 | 268.6 | 10.3 | 653.7 | 89.8 | 84 |
| SBY-2-R-1 |  |  |  |  |  |  |  |  |  |  |  |  |  |  |  |  |
| SBY-2-R-1-01 | 464.9 | 222.1 | 2.094 | 0.1385 | 0.0014 | 1.1954 | 0.0233 | 0.0626 | 0.0011 | 836.1 | 7.8 | 798.5 | 10.8 | 698.2 | 43.5 | 95 |
| SBY-2-R-1-02 | 904.2 | 840.4 | 1.076 | 0.0373 | 0.0007 | 0.2864 | 0.0068 | 0.0558 | 0.0010 | 235.9 | 4.0 | 255.7 | 5.4 | 442.6 | 8.3 | 91 |
| SBY-2-R-1-03 | 333.7 | 214.8 | 1.554 | 0.0433 | 0.0007 | 0.3342 | 0.0115 | 0.0562 | 0.0019 | 273.3 | 4.5 | 292.8 | 8.7 | 457.5 | 74.1 | 93 |
| SBY-2-R-1-04 | 97.6 | 149.1 | 0.654 | 0.1468 | 0.0028 | 1.7513 | 0.0819 | 0.0854 | 0.0029 | 882.9 | 15.7 | 1027.7 | 30.2 | 1324.1 | 64.8 | 84 |
| SBY-2-R-1-05 | 446.7 | 544.9 | 0.820 | 0.3554 | 0.0037 | 6.0345 | 0.0939 | 0.1230 | 0.0012 | 1960.4 | 17.8 | 1980.8 | 13.6 | 2066.7 | 17.1 | 98 |
| SBY-2-R-1-06 | 297.7 | 320.6 | 0.928 | 0.1705 | 0.0022 | 1.6830 | 0.0312 | 0.0715 | 0.0010 | 1015.1 | 12.0 | 1002.1 | 11.8 | 972.2 | 33.5 | 98 |
| SBY-2-R-1-07 | 249.9 | 314.2 | 0.795 | 0.1721 | 0.0018 | 1.7002 | 0.0268 | 0.0716 | 0.0009 | 1023.9 | 9.8 | 1008.6 | 10.1 | 975.9 | 24.5 | 98 |
| SBY-2-R-1-08 | 46.8 | 383.1 | 0.122 | 0.0723 | 0.0010 | 0.5632 | 0.0110 | 0.0565 | 0.0010 | 450.3 | 6.0 | 453.6 | 7.2 | 472.3 | 43.5 | 99 |
| SBY-2-R-1-09 | 155.5 | 180.6 | 0.861 | 0.2799 | 0.0041 | 4.0480 | 0.1039 | 0.1045 | 0.0020 | 1591.0 | 20.5 | 1643.9 | 20.9 | 1706.5 | 36.3 | 96 |
| SBY-2-R-1-10 | 89.3 | 145.0 | 0.616 | 0.1719 | 0.0020 | 1.7115 | 0.0378 | 0.0722 | 0.0014 | 1022.6 | 10.9 | 1012.8 | 14.2 | 992.3 | 40.7 | 99 |
| SBY-2-R-1-11 | 140.8 | 310.7 | 0.453 | 0.0491 | 0.0007 | 0.3469 | 0.0105 | 0.0513 | 0.0015 | 308.9 | 4.0 | 302.4 | 7.9 | 253.8 | 66.7 | 97 |
| SBY-2-R-1-12 | 1314.7 | 1252.9 | 1.049 | 0.0629 | 0.0017 | 0.5092 | 0.0131 | 0.0590 | 0.0009 | 393.1 | 10.1 | 417.9 | 8.8 | 568.6 | 39.8 | 93 |
| SBY-2-R-1-13 | 125.8 | 155.8 | 0.807 | 0.3200 | 0.0040 | 5.0767 | 0.0754 | 0.1152 | 0.0015 | 1789.8 | 19.4 | 1832.2 | 12.6 | 1883.3 | 24.4 | 97 |
| SBY-2-R-1-14 | 455.9 | 403.7 | 1.129 | 0.5164 | 0.0082 | 11.8071 | 0.1950 | 0.1658 | 0.0015 | 2683.8 | 34.6 | 2589.2 | 15.5 | 2516.4 | 15.0 | 96 |
| SBY-2-R-1-15 | 1600.8 | 1680.1 | 0.953 | 0.0695 | 0.0021 | 0.5655 | 0.0144 | 0.0596 | 0.0011 | 433.2 | 12.5 | 455.1 | 9.3 | 590.8 | 40.7 | 95 |
| SBY-2-R-1-16 | 182.1 | 246.1 | 0.740 | 0.1680 | 0.0015 | 1.6766 | 0.0249 | 0.0724 | 0.0010 | 1001.3 | 8.3 | 999.7 | 9.5 | 998.2 | 28.5 | 99 |
| SBY-2-R-1-17 | 49.1 | 110.0 | 0.446 | 0.1686 | 0.0016 | 1.7115 | 0.0326 | 0.0737 | 0.0014 | 1004.7 | 9.0 | 1012.8 | 12.2 | 1031.5 | 37.8 | 99 |
| SBY-2-R-1-18 | 18.6 | 780.9 | 0.024 | 0.1734 | 0.0019 | 1.7126 | 0.0242 | 0.0717 | 0.0009 | 1030.6 | 10.5 | 1013.3 | 9.1 | 988.9 | 25.9 | 98 |
| SBY-2-R-1-19 | 141.6 | 87.5 | 1.618 | 0.1653 | 0.0015 | 1.5696 | 0.0387 | 0.0689 | 0.0017 | 986.3 | 8.2 | 958.2 | 15.3 | 894.4 | 51.9 | 97 |
| SBY-2-R-1-20 | 191.3 | 264.3 | 0.724 | 0.2766 | 0.0046 | 3.8749 | 0.0826 | 0.1015 | 0.0014 | 1574.2 | 23.3 | 1608.5 | 17.2 | 1651.5 | 25.9 | 97 |
| SBY-2-R-1-21 | 328.9 | 493.1 | 0.667 | 0.1540 | 0.0017 | 1.4719 | 0.0239 | 0.0693 | 0.0009 | 923.6 | 9.5 | 918.9 | 9.8 | 907.1 | 60.2 | 99 |
| SBY-2-R-1-22 | 194.5 | 178.7 | 1.088 | 0.4342 | 0.0087 | 9.5902 | 0.2297 | 0.1599 | 0.0019 | 2324.5 | 39.0 | 2396.2 | 22.0 | 2454.0 | 20.4 | 96 |
| SBY-2-R-1-23 | 58.2 | 96.2 | 0.605 | 0.2099 | 0.0037 | 3.6066 | 0.1321 | 0.1237 | 0.0032 | 1228.4 | 19.5 | 1551.0 | 29.1 | 2009.6 | 44.9 | 76 |
| SBY-2-R-1-24 | 71.6 | 111.9 | 0.640 | 0.1279 | 0.0013 | 1.1834 | 0.0329 | 0.0672 | 0.0019 | 775.8 | 7.5 | 792.9 | 15.3 | 855.6 | 60.0 | 97 |
| SBY-2-R-1-25 | 244.5 | 538.2 | 0.454 | 0.0658 | 0.0006 | 0.4793 | 0.0099 | 0.0528 | 0.0010 | 410.9 | 3.8 | 397.6 | 6.8 | 320.4 | 42.6 | 96 |
| SBY-2-R-1-26 | 91.2 | 123.5 | 0.739 | 0.4718 | 0.0040 | 11.9260 | 0.1464 | 0.1833 | 0.0021 | 2491.4 | 17.5 | 2598.6 | 11.5 | 2683.0 | 18.5 | 95 |
| SBY-2-R-1-27 | 220.6 | 321.8 | 0.686 | 0.2016 | 0.0025 | 2.9231 | 0.0550 | 0.1050 | 0.0014 | 1183.9 | 13.6 | 1387.9 | 14.2 | 1714.5 | 19.6 | 84 |
| SBY-2-R-1-28 | 385.5 | 400.7 | 0.962 | 0.1862 | 0.0018 | 2.0136 | 0.0303 | 0.0784 | 0.0010 | 1100.6 | 10.1 | 1120.1 | 10.2 | 1166.7 | 24.5 | 98 |
| SBY-2-R-1-29 | 1012.1 | 591.3 | 1.712 | 0.1590 | 0.0027 | 1.5562 | 0.0312 | 0.0709 | 0.0009 | 951.4 | 14.9 | 952.9 | 12.4 | 966.7 | 27.8 | 99 |
| SBY-2-R-1-30 | 167.5 | 624.8 | 0.268 | 0.0657 | 0.0007 | 0.5146 | 0.0102 | 0.0568 | 0.0011 | 410.4 | 4.3 | 421.5 | 6.8 | 483.4 | 42.6 | 97 |
| SBY-2-R-1-31 | 107.4 | 161.3 | 0.666 | 0.2317 | 0.0023 | 2.9449 | 0.0560 | 0.0920 | 0.0014 | 1343.4 | 11.9 | 1393.5 | 14.4 | 1533.3 | 29.6 | 96 |
| SBY-2-R-1-32 | 26.0 | 208.2 | 0.125 | 0.1457 | 0.0014 | 1.4199 | 0.0254 | 0.0706 | 0.0012 | 877.0 | 7.7 | 897.3 | 10.7 | 946.3 | 33.3 | 97 |
| SBY-2-R-1-33 | 45.9 | 122.4 | 0.375 | 0.1682 | 0.0019 | 1.7572 | 0.0377 | 0.0757 | 0.0015 | 1002.4 | 10.6 | 1029.8 | 13.9 | 1087.0 | 36.1 | 97 |
| SBY-2-R-1-34 | 439.8 | 567.2 | 0.775 | 0.1496 | 0.0014 | 1.4899 | 0.0239 | 0.0721 | 0.0009 | 898.8 | 7.8 | 926.3 | 9.8 | 987.0 | 25.9 | 96 |
| SBY-2-R-1-35 | 160.4 | 367.8 | 0.436 | 0.1705 | 0.0024 | 1.7826 | 0.0389 | 0.0756 | 0.0012 | 1014.9 | 12.9 | 1039.1 | 14.2 | 1084.9 | 31.5 | 97 |
| SBY-2-R-1-36 | 91.9 | 320.7 | 0.287 | 0.1412 | 0.0015 | 1.3372 | 0.0231 | 0.0687 | 0.0012 | 851.4 | 8.4 | 862.0 | 10.1 | 900.0 | 37.0 | 98 |
| SBY-2-R-1-37 | 600.5 | 797.5 | 0.753 | 0.0625 | 0.0008 | 0.4758 | 0.0090 | 0.0551 | 0.0009 | 391.0 | 4.6 | 395.2 | 6.2 | 416.7 | 32.4 | 98 |
| SBY-2-R-1-38 | 135.1 | 112.0 | 1.206 | 0.5221 | 0.0059 | 16.5306 | 0.2365 | 0.2293 | 0.0027 | 2708.2 | 25.1 | 2908.0 | 13.7 | 3047.2 | 19.4 | 92 |
| SBY-2-R-1-39 | 351.8 | 597.7 | 0.589 | 0.1692 | 0.0021 | 1.7485 | 0.0310 | 0.0747 | 0.0010 | 1007.9 | 11.4 | 1026.6 | 11.4 | 1061.1 | 26.7 | 98 |
| SBY-2-R-1-40 | 91.8 | 301.1 | 0.305 | 0.1684 | 0.0018 | 1.7511 | 0.0291 | 0.0753 | 0.0012 | 1003.3 | 9.9 | 1027.6 | 10.7 | 1075.9 | 26.9 | 97 |
| SBY-2-R-1-41 | 172.5 | 95.1 | 1.814 | 0.0642 | 0.0009 | 0.5130 | 0.0259 | 0.0580 | 0.0028 | 400.9 | 5.5 | 420.5 | 17.4 | 531.5 | 99.1 | 95 |
| SBY-2-R-1-42 | 154.5 | 170.4 | 0.906 | 0.1478 | 0.0015 | 1.5079 | 0.0315 | 0.0738 | 0.0014 | 888.9 | 8.6 | 933.6 | 12.8 | 1038.9 | 38.4 | 95 |
| SBY-2-R-1-43 | 102.9 | 150.0 | 0.686 | 0.0690 | 0.0012 | 0.5714 | 0.0174 | 0.0598 | 0.0014 | 430.3 | 7.0 | 458.9 | 11.2 | 594.5 | 56.5 | 93 |
| SBY-2-R-1-44 | 286.5 | 250.6 | 1.143 | 0.4081 | 0.0045 | 9.2135 | 0.1322 | 0.1635 | 0.0017 | 2206.0 | 20.4 | 2359.5 | 13.1 | 2492.3 | 17.1 | 93 |
| SBY-2-R-1-45 | 219.6 | 183.2 | 1.199 | 0.2327 | 0.0050 | 3.2719 | 0.0783 | 0.1019 | 0.0014 | 1348.7 | 25.9 | 1474.4 | 18.6 | 1661.1 | 24.5 | 91 |
| SBY-2-R-1-46 | 250.3 | 927.8 | 0.270 | 0.0643 | 0.0009 | 0.5046 | 0.0096 | 0.0569 | 0.0008 | 401.5 | 5.6 | 414.8 | 6.5 | 487.1 | 29.6 | 96 |
| SBY-2-R-1-47 | 405.0 | 476.4 | 0.850 | 0.0248 | 0.0003 | 0.1726 | 0.0065 | 0.0504 | 0.0017 | 157.6 | 2.2 | 161.7 | 5.6 | 213.0 | 50.0 | 97 |
| SBY-2-R-1-48 | 263.0 | 135.8 | 1.937 | 0.0623 | 0.0006 | 0.4833 | 0.0134 | 0.0564 | 0.0016 | 389.5 | 3.9 | 400.3 | 9.2 | 477.8 | 30.6 | 97 |
| SBY-2-R-1-49 | 364.2 | 456.2 | 0.798 | 0.0630 | 0.0008 | 0.4916 | 0.0102 | 0.0566 | 0.0010 | 393.9 | 4.7 | 406.0 | 6.9 | 476.0 | 43.5 | 96 |
| SBY-2-R-1-50 | 18.4 | 507.3 | 0.036 | 0.1432 | 0.0024 | 1.4194 | 0.0339 | 0.0717 | 0.0011 | 862.9 | 13.3 | 897.1 | 14.2 | 977.5 | 31.5 | 96 |
| SBY-2-R-1-51 | 310.3 | 241.5 | 1.285 | 0.1708 | 0.0017 | 1.7876 | 0.0313 | 0.0760 | 0.0013 | 1016.5 | 9.6 | 1041.0 | 11.4 | 1094.4 | 33.3 | 97 |
| SBY-2-R-1-52 | 137.0 | 273.3 | 0.501 | 0.1634 | 0.0016 | 1.7263 | 0.0333 | 0.0766 | 0.0013 | 975.9 | 8.9 | 1018.4 | 12.4 | 1109.3 | 34.1 | 95 |
| SBY-2-R-1-53 | 643.8 | 899.7 | 0.716 | 0.0397 | 0.0005 | 0.2797 | 0.0053 | 0.0512 | 0.0009 | 250.7 | 3.0 | 250.5 | 4.2 | 250.1 | 45.4 | 99 |
| SBY-2-R-1-54 | 571.5 | 981.4 | 0.582 | 0.1338 | 0.0015 | 1.2788 | 0.0193 | 0.0693 | 0.0008 | 809.3 | 8.6 | 836.3 | 8.6 | 909.3 | 24.1 | 96 |
| SBY-2-R-1-55 | 480.1 | 524.9 | 0.915 | 0.3674 | 0.0067 | 8.1868 | 0.2087 | 0.1608 | 0.0020 | 2016.9 | 31.8 | 2251.9 | 23.1 | 2463.9 | 21.0 | 88 |
| SBY-2-R-1-56 | 318.7 | 2352.1 | 0.135 | 0.1417 | 0.0022 | 1.3867 | 0.0244 | 0.0709 | 0.0007 | 854.3 | 12.5 | 883.3 | 10.4 | 966.7 | -12.0 | 96 |
| SBY-2-R-1-57 | 88.7 | 204.6 | 0.433 | 0.1853 | 0.0020 | 2.0566 | 0.0407 | 0.0804 | 0.0013 | 1095.7 | 10.9 | 1134.5 | 13.5 | 1206.5 | 31.5 | 96 |
| SBY-2-R-1-58 | 325.2 | 412.5 | 0.788 | 0.0640 | 0.0009 | 0.5008 | 0.0118 | 0.0567 | 0.0011 | 399.7 | 5.3 | 412.3 | 8.0 | 483.4 | 44.4 | 96 |
| SBY-2-R-1-59 | 122.4 | 440.4 | 0.278 | 0.0671 | 0.0008 | 0.5219 | 0.0131 | 0.0563 | 0.0013 | 418.8 | 4.8 | 426.4 | 8.7 | 464.9 | 51.8 | 98 |
| SBY-2-R-1-60 | 92.6 | 166.5 | 0.556 | 0.1551 | 0.0024 | 1.5852 | 0.0366 | 0.0740 | 0.0012 | 929.3 | 13.4 | 964.4 | 14.4 | 1042.6 | 34.4 | 96 |
| SBY-2-R-1-61 | 417.8 | 175.7 | 2.378 | 0.1242 | 0.0027 | 1.1331 | 0.0395 | 0.0657 | 0.0015 | 754.8 | 15.3 | 769.2 | 18.8 | 798.2 | 54.6 | 98 |
| SBY-2-R-1-62 | 881.2 | 524.0 | 1.682 | 0.0844 | 0.0023 | 1.0398 | 0.0266 | 0.0900 | 0.0016 | 522.1 | 13.4 | 723.8 | 13.3 | 1427.8 | 35.2 | 67 |
| SBY-2-R-1-63 | 178.9 | 302.4 | 0.592 | 0.4623 | 0.0093 | 11.0946 | 0.2666 | 0.1743 | 0.0031 | 2449.6 | 40.8 | 2531.1 | 22.4 | 2599.1 | 29.9 | 96 |
| SBY-2-R-1-64 | 433.9 | 793.3 | 0.547 | 0.1735 | 0.0024 | 1.8308 | 0.0281 | 0.0765 | 0.0008 | 1031.3 | 13.3 | 1056.6 | 10.1 | 1109.3 | 22.2 | 97 |
| SBY-2-R-1-65 | 69.4 | 207.6 | 0.334 | 0.1417 | 0.0014 | 1.3761 | 0.0306 | 0.0704 | 0.0014 | 854.2 | 8.1 | 878.8 | 13.1 | 938.9 | 41.5 | 97 |
| SBY-2-R-1-66 | 127.0 | 239.1 | 0.531 | 0.0657 | 0.0008 | 0.5057 | 0.0151 | 0.0559 | 0.0017 | 410.4 | 4.7 | 415.5 | 10.2 | 455.6 | 66.7 | 98 |
| SBY-2-R-1-67 | 393.1 | 644.5 | 0.610 | 0.0426 | 0.0004 | 0.3149 | 0.0080 | 0.0535 | 0.0013 | 269.1 | 2.7 | 278.0 | 6.2 | 350.1 | 84.3 | 96 |
| SBY-2-R-1-68 | 502.8 | 709.4 | 0.709 | 0.0405 | 0.0004 | 0.2954 | 0.0054 | 0.0530 | 0.0009 | 255.7 | 2.6 | 262.8 | 4.3 | 327.8 | 40.7 | 97 |
| SBY-2-R-1-69 | 78.8 | 313.8 | 0.251 | 0.2789 | 0.0082 | 4.0955 | 0.1628 | 0.1053 | 0.0016 | 1585.6 | 41.5 | 1653.4 | 32.4 | 1720.4 | 28.4 | 95 |
| SBY-2-R-1-70 | 267.8 | 453.7 | 0.590 | 0.0511 | 0.0006 | 0.3707 | 0.0089 | 0.0526 | 0.0012 | 321.6 | 3.7 | 320.2 | 6.6 | 322.3 | 53.7 | 99 |
| SBY-2-R-1-71 | 148.3 | 198.2 | 0.748 | 0.1593 | 0.0021 | 1.5507 | 0.0336 | 0.0707 | 0.0013 | 952.8 | 11.9 | 950.8 | 13.4 | 947.2 | 37.8 | 99 |
| SBY-2-R-1-72 | 354.8 | 704.6 | 0.504 | 0.1154 | 0.0010 | 1.0303 | 0.0144 | 0.0648 | 0.0009 | 703.9 | 5.9 | 719.1 | 7.2 | 768.5 | 28.5 | 97 |
| SBY-2-R-1-73 | 92.5 | 141.9 | 0.652 | 0.0314 | 0.0004 | 0.2941 | 0.0168 | 0.0683 | 0.0039 | 199.1 | 2.5 | 261.8 | 13.1 | 876.9 | 118.5 | 72 |
| SBY-2-R-1-74 | 438.5 | 613.5 | 0.715 | 0.1567 | 0.0019 | 1.6141 | 0.0325 | 0.0746 | 0.0011 | 938.4 | 10.6 | 975.7 | 12.6 | 1057.4 | 30.4 | 96 |
| SBY-2-R-1-75 | 231.4 | 382.2 | 0.605 | 0.0620 | 0.0007 | 0.4969 | 0.0129 | 0.0580 | 0.0012 | 387.9 | 4.5 | 409.6 | 8.8 | 527.8 | 46.3 | 94 |
| SBY-2-R-1-76 | 143.0 | 154.9 | 0.924 | 0.5028 | 0.0065 | 13.7034 | 0.2075 | 0.1978 | 0.0022 | 2625.6 | 27.9 | 2729.4 | 14.3 | 2809.3 | 17.9 | 96 |
| SBY-2-R-1-77 | 123.4 | 2049.0 | 0.060 | 0.3636 | 0.0053 | 9.3151 | 0.1488 | 0.1860 | 0.0020 | 1999.1 | 25.3 | 2369.5 | 14.6 | 2707.1 | 12.2 | 83 |
| SBY-2-R-1-78 | 198.0 | 521.0 | 0.380 | 0.0974 | 0.0011 | 0.8246 | 0.0187 | 0.0614 | 0.0012 | 599.3 | 6.7 | 610.6 | 10.4 | 650.0 | 42.6 | 98 |
| SBY-2-R-1-79 | 6.9 | 1.4 | 4.908 | 0.9407 | 0.4669 | 180.3778 | 54.9833 | 0.0000 | 0.0000 | 4274.4 | 1581.7 | 5280.6 | 317.8 | — | — | 78 |
| SBY-2-R-1-80 | 918.8 | 866.6 | 1.060 | 0.0689 | 0.0008 | 0.5531 | 0.0119 | 0.0581 | 0.0010 | 429.6 | 4.5 | 447.0 | 7.8 | 600.0 | 38.9 | 96 |
| SBY-2-R-1-81 | 650.2 | 485.0 | 1.341 | 0.0325 | 0.0004 | 0.2743 | 0.0112 | 0.0610 | 0.0022 | 206.0 | 2.6 | 246.1 | 8.9 | 638.9 | 77.8 | 82 |
| SBY-2-R-1-82 | 202.0 | 317.6 | 0.636 | 0.1524 | 0.0014 | 1.4827 | 0.0248 | 0.0705 | 0.0010 | 914.6 | 7.8 | 923.3 | 10.1 | 942.6 | 25.0 | 99 |
| SBY-2-R-1-83 | 83.8 | 190.4 | 0.440 | 0.2882 | 0.0279 | 6.5741 | 1.7797 | 0.1223 | 0.0119 | 1632.4 | 139.7 | 2055.9 | 243.1 | 1990.7 | 173.5 | 77 |
| SBY-2-R-1-84 | 106.0 | 359.3 | 0.295 | 0.4288 | 0.0045 | 10.3018 | 0.1410 | 0.1741 | 0.0018 | 2300.4 | 20.4 | 2462.3 | 12.7 | 2597.2 | 17.0 | 93 |
| SBY-2-R-1-85 | 331.1 | 206.1 | 1.607 | 0.0340 | 0.0014 | 1.1523 | 0.1158 | 0.2375 | 0.0132 | 215.3 | 8.8 | 778.3 | 54.7 | 3103.4 | 89.4 | -14 |
| SBY-2-R-1-86 | 101.9 | 557.2 | 0.183 | 0.1455 | 0.0020 | 1.5803 | 0.0301 | 0.0786 | 0.0010 | 875.7 | 11.1 | 962.5 | 11.8 | 1164.8 | 27.0 | 90 |
| SBY-2-R-1-87 | 262.4 | 346.5 | 0.757 | 0.0358 | 0.0004 | 0.2634 | 0.0084 | 0.0533 | 0.0017 | 227.0 | 2.3 | 237.4 | 6.7 | 342.7 | 39.8 | 95 |
| SBY-2-R-1-88 | 295.3 | 209.3 | 1.411 | 0.0844 | 0.0013 | 0.6758 | 0.0174 | 0.0580 | 0.0013 | 522.4 | 7.9 | 524.2 | 10.6 | 531.5 | 54.6 | 99 |
| SBY-2-R-1-89 | 143.0 | 202.2 | 0.708 | 0.3840 | 0.0071 | 7.9048 | 0.2024 | 0.1485 | 0.0021 | 2095.1 | 33.1 | 2220.2 | 23.1 | 2328.7 | 23.9 | 94 |
| SBY-2-R-1-90 | 63.2 | 80.1 | 0.789 | 0.1166 | 0.0018 | 1.0627 | 0.0320 | 0.0661 | 0.0018 | 711.0 | 10.4 | 735.1 | 15.8 | 809.3 | 63.9 | 96 |
| JF-39-R-1 |  |  |  |  |  |  |  |  |  |  |  |  |  |  |  |  |
| JF-39-R-1-01 | 75.4 | 416.6 | 0.181 | 0.0585 | 0.0008 | 0.5229 | 0.0160 | 0.0646 | 0.0018 | 366.6 | 4.8 | 427.1 | 10.7 | 761.1 | 59.3 | 84 |
| JF-39-R-1-02 | 266.5 | 438.4 | 0.608 | 0.0354 | 0.0009 | 0.2497 | 0.0101 | 0.0515 | 0.0017 | 224.5 | 5.8 | 226.3 | 8.2 | 264.9 | 78.7 | 99 |
| JF-39-R-1-03 | 81.5 | 344.9 | 0.236 | 0.1681 | 0.0020 | 2.4114 | 0.0478 | 0.1040 | 0.0018 | 1001.5 | 11.2 | 1246.0 | 14.2 | 1698.2 | 31.5 | 78 |
| JF-39-R-1-04 | 162.4 | 341.7 | 0.475 | 0.0394 | 0.0007 | 0.2915 | 0.0110 | 0.0538 | 0.0019 | 249.0 | 4.2 | 259.7 | 8.7 | 364.9 | 79.6 | 95 |
| JF-39-R-1-05 | 171.7 | 589.6 | 0.291 | 0.0392 | 0.0006 | 0.2778 | 0.0076 | 0.0516 | 0.0013 | 247.6 | 3.8 | 248.9 | 6.1 | 264.9 | 57.4 | 99 |
| JF-39-R-1-06 | 107.3 | 408.2 | 0.263 | 0.0386 | 0.0006 | 0.2703 | 0.0090 | 0.0511 | 0.0017 | 244.1 | 3.6 | 242.9 | 7.2 | 242.7 | 75.9 | 99 |
| JF-39-R-1-07 | 56.7 | 266.3 | 0.213 | 0.0397 | 0.0006 | 0.2894 | 0.0123 | 0.0533 | 0.0023 | 251.1 | 4.0 | 258.1 | 9.7 | 342.7 | 96.3 | 97 |
| JF-39-R-1-08 | 270.9 | 228.3 | 1.186 | 0.1960 | 0.0037 | 2.2838 | 0.0616 | 0.0844 | 0.0016 | 1153.6 | 19.9 | 1207.3 | 19.0 | 1301.9 | 38.1 | 95 |
| JF-39-R-1-09 | 95.8 | 322.3 | 0.297 | 0.0391 | 0.0006 | 0.2952 | 0.0140 | 0.0552 | 0.0026 | 247.0 | 4.0 | 262.6 | 11.0 | 420.4 | 100.9 | 93 |
| JF-39-R-1-10 | 149.0 | 421.6 | 0.353 | 0.0393 | 0.0005 | 0.2862 | 0.0099 | 0.0531 | 0.0018 | 248.3 | 3.3 | 255.6 | 7.8 | 331.5 | 75.9 | 97 |
| JF-39-R-1-11 | 114.1 | 334.4 | 0.341 | 0.0445 | 0.0015 | 0.3429 | 0.0214 | 0.0544 | 0.0022 | 280.8 | 9.6 | 299.4 | 16.2 | 387.1 | 90.7 | 93 |
| JF-39-R-1-12 | 128.8 | 394.1 | 0.327 | 0.0392 | 0.0006 | 0.2874 | 0.0097 | 0.0538 | 0.0019 | 247.6 | 4.0 | 256.5 | 7.6 | 364.9 | 77.8 | 96 |
| JF-39-R-1-13 | 106.7 | 387.1 | 0.276 | 0.0382 | 0.0006 | 0.2748 | 0.0109 | 0.0521 | 0.0019 | 241.7 | 3.9 | 246.6 | 8.7 | 287.1 | 87.0 | 97 |
| JF-39-R-1-14 | 93.5 | 537.2 | 0.174 | 0.0380 | 0.0005 | 0.2878 | 0.0093 | 0.0549 | 0.0017 | 240.2 | 3.2 | 256.8 | 7.3 | 405.6 | 66.7 | 93 |
| JF-39-R-1-15 | 185.9 | 706.6 | 0.263 | 0.0393 | 0.0005 | 0.2854 | 0.0077 | 0.0530 | 0.0015 | 248.3 | 3.3 | 254.9 | 6.1 | 331.5 | 63.0 | 97 |
| JF-39-R-1-16 | 130.6 | 427.7 | 0.305 | 0.0386 | 0.0006 | 0.2928 | 0.0108 | 0.0552 | 0.0019 | 244.2 | 3.7 | 260.8 | 8.5 | 420.4 | 77.8 | 93 |
| JF-39-R-1-17 | 117.2 | 256.6 | 0.457 | 0.0393 | 0.0007 | 0.2649 | 0.0134 | 0.0489 | 0.0023 | 248.7 | 4.1 | 238.6 | 10.7 | 142.7 | 111.1 | 95 |
| JF-39-R-1-18 | 85.7 | 295.7 | 0.290 | 0.0399 | 0.0007 | 0.2958 | 0.0118 | 0.0538 | 0.0020 | 252.0 | 4.2 | 263.1 | 9.3 | 361.2 | 83.3 | 95 |
| JF-39-R-1-19 | 102.7 | 322.1 | 0.319 | 0.0400 | 0.0006 | 0.2916 | 0.0121 | 0.0526 | 0.0020 | 252.7 | 4.0 | 259.8 | 9.5 | 322.3 | 82.4 | 97 |
| JF-39-R-1-20 | 56.7 | 278.7 | 0.204 | 0.0358 | 0.0011 | 0.2829 | 0.0170 | 0.0584 | 0.0037 | 226.7 | 6.7 | 253.0 | 13.5 | 542.6 | 138.9 | 89 |
| JF-39-R-1-21 | 168.6 | 1104.7 | 0.153 | 0.0383 | 0.0006 | 0.2712 | 0.0075 | 0.0514 | 0.0013 | 242.2 | 3.8 | 243.7 | 6.0 | 261.2 | 59.2 | 99 |
| JF-39-R-1-22 | 92.5 | 391.4 | 0.236 | 0.0389 | 0.0006 | 0.2855 | 0.0102 | 0.0537 | 0.0019 | 245.8 | 3.7 | 255.0 | 8.1 | 366.7 | 79.6 | 96 |
| JF-39-R-1-23 | 131.1 | 385.6 | 0.340 | 0.0372 | 0.0006 | 0.2819 | 0.0108 | 0.0555 | 0.0022 | 235.2 | 3.5 | 252.2 | 8.5 | 431.5 | 87.0 | 93 |
| JF-39-R-1-24 | 117.4 | 265.8 | 0.442 | 0.0392 | 0.0006 | 0.2788 | 0.0111 | 0.0520 | 0.0021 | 248.0 | 4.0 | 249.7 | 8.8 | 287.1 | 92.6 | 99 |
| JF-39-R-1-25 | 134.2 | 381.6 | 0.352 | 0.0386 | 0.0005 | 0.2713 | 0.0103 | 0.0512 | 0.0019 | 244.0 | 3.3 | 243.7 | 8.3 | 250.1 | 89.8 | 99 |
| JF-39-R-1-26 | 92.9 | 308.9 | 0.301 | 0.0418 | 0.0009 | 0.3130 | 0.0139 | 0.0543 | 0.0021 | 263.9 | 5.6 | 276.5 | 10.7 | 383.4 | 88.9 | 95 |
| JF-39-R-1-27 | 91.8 | 401.9 | 0.228 | 0.0393 | 0.0006 | 0.2918 | 0.0095 | 0.0537 | 0.0015 | 248.8 | 3.9 | 260.0 | 7.4 | 366.7 | 60.2 | 95 |
| JF-39-R-1-28 | 203.0 | 401.0 | 0.506 | 0.0395 | 0.0006 | 0.2908 | 0.0102 | 0.0532 | 0.0017 | 249.9 | 3.7 | 259.2 | 8.0 | 338.9 | 72.2 | 96 |
| JF-39-R-1-29 | 97.4 | 321.5 | 0.303 | 0.0402 | 0.0007 | 0.2828 | 0.0105 | 0.0511 | 0.0017 | 254.2 | 4.1 | 252.9 | 8.3 | 242.7 | 77.8 | 99 |
| JF-39-R-1-30 | 94.4 | 381.7 | 0.247 | 0.0405 | 0.0006 | 0.2847 | 0.0095 | 0.0509 | 0.0016 | 255.9 | 3.7 | 254.3 | 7.5 | 235.3 | 70.4 | 99 |
| JF-39-R-1-31 | 140.6 | 267.5 | 0.525 | 0.0378 | 0.0006 | 0.2717 | 0.0114 | 0.0527 | 0.0023 | 239.4 | 3.5 | 244.1 | 9.1 | 322.3 | 98.1 | 98 |
| JF-39-R-1-32 | 131.4 | 272.6 | 0.482 | 0.0380 | 0.0006 | 0.2816 | 0.0129 | 0.0538 | 0.0023 | 240.3 | 3.7 | 251.9 | 10.2 | 364.9 | 98.1 | 95 |
| JF-39-R-1-33 | 126.5 | 452.1 | 0.280 | 0.0390 | 0.0006 | 0.2752 | 0.0089 | 0.0514 | 0.0017 | 246.4 | 3.5 | 246.8 | 7.1 | 261.2 | 80.5 | 99 |
| JF-39-R-1-34 | 169.7 | 353.6 | 0.480 | 0.0386 | 0.0006 | 0.2755 | 0.0103 | 0.0521 | 0.0019 | 243.9 | 3.9 | 247.0 | 8.2 | 300.1 | 83.3 | 98 |
| JF-39-R-1-35 | 115.9 | 412.4 | 0.281 | 0.0459 | 0.0015 | 0.3590 | 0.0174 | 0.0563 | 0.0019 | 289.4 | 9.0 | 311.5 | 13.0 | 464.9 | 75.9 | 92 |
| JF-39-R-1-36 | 98.1 | 362.8 | 0.270 | 0.0385 | 0.0006 | 0.2709 | 0.0111 | 0.0511 | 0.0020 | 243.4 | 3.8 | 243.4 | 8.9 | 255.6 | 97.2 | 99 |
| JF-39-R-1-37 | 88.7 | 371.3 | 0.239 | 0.0381 | 0.0006 | 0.2800 | 0.0097 | 0.0536 | 0.0019 | 240.8 | 3.6 | 250.7 | 7.7 | 353.8 | 79.6 | 95 |
| JF-39-R-1-38 | 460.6 | 354.2 | 1.300 | 0.0957 | 0.0013 | 0.8845 | 0.0231 | 0.0672 | 0.0017 | 589.1 | 7.7 | 643.4 | 12.5 | 842.6 | 51.9 | 91 |
| JF-39-R-1-39 | 98.9 | 335.2 | 0.295 | 0.0373 | 0.0006 | 0.2786 | 0.0130 | 0.0541 | 0.0024 | 236.4 | 3.5 | 249.6 | 10.3 | 376.0 | 100.0 | 94 |
| JF-39-R-1-40 | 78.0 | 300.7 | 0.259 | 0.0376 | 0.0006 | 0.2758 | 0.0110 | 0.0532 | 0.0021 | 237.9 | 3.7 | 247.3 | 8.8 | 338.9 | 88.9 | 96 |
| JF-39-R-1-41 | 111.9 | 379.5 | 0.295 | 0.0379 | 0.0006 | 0.2680 | 0.0100 | 0.0515 | 0.0019 | 239.9 | 4.0 | 241.1 | 8.0 | 264.9 | 87.0 | 99 |
| JF-39-R-1-42 | 216.9 | 516.8 | 0.420 | 0.0304 | 0.0004 | 0.2305 | 0.0082 | 0.0554 | 0.0022 | 193.1 | 2.5 | 210.6 | 6.8 | 427.8 | 91.7 | 91 |
| JF-39-R-1-43 | 189.4 | 1072.8 | 0.177 | 0.0381 | 0.0004 | 0.2647 | 0.0077 | 0.0504 | 0.0015 | 241.3 | 2.7 | 238.4 | 6.2 | 213.0 | 70.4 | 98 |
| JF-39-R-1-44 | 67.1 | 246.8 | 0.272 | 0.0384 | 0.0006 | 0.2690 | 0.0117 | 0.0508 | 0.0021 | 242.9 | 3.9 | 241.9 | 9.4 | 231.6 | 96.3 | 99 |
| JF-39-R-1-45 | 182.2 | 654.8 | 0.278 | 0.0677 | 0.0031 | 0.5836 | 0.0331 | 0.0605 | 0.0015 | 422.5 | 18.5 | 466.8 | 21.2 | 633.4 | 51.8 | 90 |
| JF-39-R-1-46 | 107.4 | 289.7 | 0.371 | 0.0386 | 0.0006 | 0.2886 | 0.0124 | 0.0543 | 0.0023 | 244.4 | 3.8 | 257.5 | 9.8 | 383.4 | 94.4 | 94 |
| JF-39-R-1-47 | 125.1 | 419.6 | 0.298 | 0.0380 | 0.0006 | 0.2727 | 0.0098 | 0.0521 | 0.0018 | 240.6 | 3.4 | 244.9 | 7.8 | 300.1 | 79.6 | 98 |
| JF-39-R-1-48 | 83.0 | 347.6 | 0.239 | 0.0387 | 0.0005 | 0.2852 | 0.0105 | 0.0538 | 0.0020 | 244.8 | 3.4 | 254.8 | 8.3 | 361.2 | 83.3 | 96 |
| JF-39-R-1-49 | 121.4 | 318.3 | 0.382 | 0.0390 | 0.0006 | 0.2851 | 0.0119 | 0.0535 | 0.0023 | 246.6 | 3.6 | 254.7 | 9.4 | 350.1 | 93.5 | 96 |
| JF-39-R-1-50 | 146.2 | 651.3 | 0.224 | 0.0390 | 0.0006 | 0.2724 | 0.0088 | 0.0507 | 0.0015 | 246.8 | 3.7 | 244.6 | 7.1 | 233.4 | 73.1 | 99 |
| JF-39-R-1-51 | 114.0 | 434.1 | 0.263 | 0.0383 | 0.0007 | 0.2748 | 0.0094 | 0.0526 | 0.0018 | 242.5 | 4.2 | 246.5 | 7.5 | 309.3 | 75.9 | 98 |
| JF-39-R-1-52 | 253.4 | 658.0 | 0.385 | 0.1563 | 0.0027 | 2.2547 | 0.0471 | 0.1048 | 0.0017 | 936.0 | 15.0 | 1198.3 | 14.7 | 1710.8 | 31.3 | 75 |
| JF-39-R-1-53 | 97.5 | 334.3 | 0.292 | 0.0387 | 0.0006 | 0.2563 | 0.0113 | 0.0483 | 0.0021 | 244.7 | 4.0 | 231.7 | 9.1 | 122.3 | 103.7 | 94 |
| JF-39-R-1-54 | 101.6 | 379.3 | 0.268 | 0.0390 | 0.0007 | 0.2694 | 0.0093 | 0.0506 | 0.0017 | 246.6 | 4.1 | 242.2 | 7.4 | 220.4 | 77.8 | 98 |
| JF-39-R-1-55 | 75.0 | 334.7 | 0.224 | 0.0392 | 0.0008 | 0.2918 | 0.0142 | 0.0542 | 0.0024 | 247.7 | 4.7 | 260.0 | 11.1 | 388.9 | 100.0 | 95 |
| JF-39-R-1-56 | 82.3 | 218.1 | 0.377 | 0.0387 | 0.0007 | 0.2894 | 0.0140 | 0.0550 | 0.0028 | 245.0 | 4.1 | 258.1 | 11.0 | 413.0 | 112.0 | 94 |
| JF-39-R-1-57 | 96.1 | 379.4 | 0.253 | 0.0390 | 0.0006 | 0.2715 | 0.0097 | 0.0506 | 0.0018 | 246.5 | 3.9 | 243.9 | 7.8 | 233.4 | 79.6 | 98 |
| JF-39-R-1-58 | 118.7 | 389.1 | 0.305 | 0.0391 | 0.0006 | 0.2765 | 0.0116 | 0.0517 | 0.0022 | 247.0 | 3.5 | 247.9 | 9.2 | 272.3 | 96.3 | 99 |
| JF-39-R-1-59 | 127.3 | 212.1 | 0.600 | 0.0406 | 0.0007 | 0.2790 | 0.0128 | 0.0505 | 0.0024 | 256.7 | 4.6 | 249.9 | 10.2 | 216.7 | 109.2 | 97 |
| JF-39-R-1-60 | 122.6 | 393.8 | 0.311 | 0.0393 | 0.0006 | 0.2886 | 0.0106 | 0.0535 | 0.0019 | 248.3 | 3.7 | 257.4 | 8.3 | 346.4 | 81.5 | 96 |
| JF-39-R-1-61 | 120.7 | 424.0 | 0.285 | 0.0395 | 0.0007 | 0.3034 | 0.0123 | 0.0555 | 0.0020 | 249.5 | 4.1 | 269.0 | 9.6 | 431.5 | 86.1 | 92 |
| JF-39-R-1-62 | 124.8 | 481.8 | 0.259 | 0.0395 | 0.0006 | 0.2979 | 0.0101 | 0.0548 | 0.0018 | 249.6 | 3.7 | 264.8 | 7.9 | 466.7 | 72.2 | 94 |
| JF-39-R-1-63 | 80.7 | 344.5 | 0.234 | 0.0383 | 0.0009 | 0.2752 | 0.0128 | 0.0523 | 0.0024 | 242.1 | 5.6 | 246.8 | 10.2 | 301.9 | 137.9 | 98 |
| JF-39-R-1-64 | 87.4 | 391.7 | 0.223 | 0.0396 | 0.0007 | 0.2988 | 0.0113 | 0.0549 | 0.0020 | 250.4 | 4.3 | 265.4 | 8.8 | 405.6 | 83.3 | 94 |
| JF-39-R-1-65 | 107.9 | 392.3 | 0.275 | 0.0390 | 0.0005 | 0.2604 | 0.0087 | 0.0484 | 0.0016 | 246.8 | 3.3 | 235.0 | 7.0 | 116.8 | 75.9 | 95 |
| JF-39-R-1-66 | 147.0 | 578.0 | 0.254 | 0.1640 | 0.0029 | 1.7795 | 0.0429 | 0.0784 | 0.0013 | 979.0 | 15.9 | 1038.0 | 15.7 | 1166.7 | 34.4 | 94 |
| JF-39-R-1-67 | 364.9 | 606.7 | 0.601 | 0.1222 | 0.0020 | 1.3020 | 0.0307 | 0.0770 | 0.0013 | 743.0 | 11.3 | 846.6 | 13.6 | 1120.4 | 33.3 | 86 |
| JF-39-R-1-68 | 63.2 | 128.2 | 0.493 | 0.0390 | 0.0008 | 0.2875 | 0.0205 | 0.0538 | 0.0037 | 246.4 | 5.0 | 256.6 | 16.1 | 364.9 | 162.0 | 95 |
| JF-39-R-1-69 | 89.8 | 208.8 | 0.430 | 0.0391 | 0.0007 | 0.2708 | 0.0169 | 0.0506 | 0.0032 | 247.1 | 4.3 | 243.4 | 13.5 | 220.4 | 141.6 | 98 |
| JF-39-R-1-70 | 143.5 | 474.1 | 0.303 | 0.0392 | 0.0006 | 0.2785 | 0.0088 | 0.0519 | 0.0017 | 247.6 | 3.4 | 249.4 | 7.0 | 279.7 | 71.3 | 99 |
| JF-39-R-1-71 | 86.8 | 297.3 | 0.292 | 0.0400 | 0.0006 | 0.2968 | 0.0148 | 0.0543 | 0.0028 | 252.9 | 3.8 | 263.9 | 11.6 | 383.4 | 114.8 | 95 |
| JF-39-R-1-72 | 97.0 | 334.8 | 0.290 | 0.0386 | 0.0005 | 0.2733 | 0.0111 | 0.0511 | 0.0020 | 244.2 | 3.3 | 245.4 | 8.9 | 242.7 | 88.9 | 99 |
| JF-39-R-1-73 | 110.6 | 404.9 | 0.273 | 0.0391 | 0.0005 | 0.2973 | 0.0092 | 0.0552 | 0.0017 | 247.3 | 3.2 | 264.3 | 7.2 | 420.4 | 66.7 | 93 |
| JF-39-R-1-74 | 72.6 | 278.3 | 0.261 | 0.0403 | 0.0007 | 0.2661 | 0.0106 | 0.0486 | 0.0021 | 254.6 | 4.3 | 239.6 | 8.5 | 127.9 | 100.0 | 93 |
| JF-39-R-1-75 | 101.8 | 448.6 | 0.227 | 0.0394 | 0.0007 | 0.3060 | 0.0127 | 0.0561 | 0.0021 | 249.2 | 4.2 | 271.1 | 9.9 | 457.5 | 85.2 | 91 |
| JF-39-R-1-76 | 85.3 | 324.5 | 0.263 | 0.0390 | 0.0007 | 0.2747 | 0.0121 | 0.0510 | 0.0021 | 246.5 | 4.6 | 246.4 | 9.6 | 239.0 | 94.4 | 99 |
| JF-39-R-1-77 | 96.5 | 387.8 | 0.249 | 0.0392 | 0.0005 | 0.3072 | 0.0093 | 0.0571 | 0.0018 | 247.7 | 3.4 | 272.0 | 7.2 | 494.5 | 63.9 | 90 |
| JF-39-R-1-78 | 83.1 | 344.3 | 0.241 | 0.0393 | 0.0006 | 0.2732 | 0.0097 | 0.0508 | 0.0019 | 248.3 | 3.6 | 245.3 | 7.7 | 231.6 | 88.0 | 98 |
| JF-39-R-1-79 | 144.4 | 488.9 | 0.295 | 0.0395 | 0.0006 | 0.2956 | 0.0118 | 0.0542 | 0.0020 | 249.6 | 3.9 | 263.0 | 9.2 | 388.9 | 81.5 | 94 |
| JF-39-R-1-80 | 49.3 | 186.0 | 0.265 | 0.0401 | 0.0007 | 0.2924 | 0.0167 | 0.0539 | 0.0034 | 253.7 | 4.4 | 260.4 | 13.1 | 368.6 | 142.6 | 97 |
| JF-43-R-1 |  |  |  |  |  |  |  |  |  |  |  |  |  |  |  |  |
| JF-43-R-1-01 | 160.3 | 321.2 | 0.499 | 0.0401 | 0.0006 | 0.2988 | 0.0120 | 0.0539 | 0.0021 | 253.2 | 3.6 | 265.5 | 9.4 | 368.6 | 87.0 | 95 |
| JF-43-R-1-02 | 83.9 | 156.1 | 0.537 | 0.4789 | 0.0073 | 10.9424 | 0.2188 | 0.1650 | 0.0026 | 2522.6 | 31.7 | 2518.2 | 18.6 | 2507.1 | 60.0 | 99 |
| JF-43-R-1-03 | 100.1 | 360.0 | 0.278 | 0.4702 | 0.0062 | 10.7604 | 0.1740 | 0.1654 | 0.0023 | 2484.5 | 27.0 | 2502.7 | 15.0 | 2522.2 | 23.1 | 99 |
| JF-43-R-1-04 | 114.3 | 302.3 | 0.378 | 0.2996 | 0.0033 | 4.5212 | 0.0770 | 0.1089 | 0.0016 | 1689.4 | 16.3 | 1734.9 | 14.2 | 1783.3 | 27.6 | 97 |
| JF-43-R-1-05 | 298.9 | 3730.2 | 0.080 | 0.0000 | 0.0000 | 0.0000 | 0.0000 | 0.0664 | 0.0012 | 0.0 | 0.0 | 0.0 | 0.0 | 816.7 | 33.2 | — |
| JF-43-R-1-06 | 15.2 | 853.2 | 0.018 | 0.1631 | 0.0022 | 1.5979 | 0.0294 | 0.0708 | 0.0011 | 974.2 | 11.9 | 969.4 | 11.5 | 950.0 | 30.4 | 99 |
| JF-43-R-1-07 | 92.2 | 621.4 | 0.148 | 0.4629 | 0.0073 | 10.6043 | 0.1935 | 0.1657 | 0.0021 | 2452.4 | 32.4 | 2489.1 | 16.9 | 2516.7 | 21.1 | 98 |
| JF-43-R-1-08 | 186.8 | 142.6 | 1.311 | 0.2940 | 0.0036 | 4.7813 | 0.0926 | 0.1177 | 0.0021 | 1661.7 | 18.1 | 1781.6 | 16.3 | 1921.3 | 26.9 | 93 |
| JF-43-R-1-09 | 1460.7 | 950.0 | 1.538 | 0.1805 | 0.0029 | 2.6049 | 0.0533 | 0.1043 | 0.0015 | 1069.5 | 15.9 | 1302.0 | 15.0 | 1702.2 | 26.1 | 80 |
| JF-43-R-1-10 | 622.6 | 493.1 | 1.263 | 0.0532 | 0.0008 | 0.4062 | 0.0110 | 0.0555 | 0.0015 | 334.3 | 4.8 | 346.1 | 8.0 | 431.5 | 63.9 | 96 |
| JF-43-R-1-11 | 224.9 | 260.3 | 0.864 | 0.0712 | 0.0013 | 0.6152 | 0.0257 | 0.0622 | 0.0022 | 443.3 | 7.8 | 486.8 | 16.2 | 679.6 | 71.3 | 90 |
| JF-43-R-1-12 | 353.2 | 329.3 | 1.073 | 0.0453 | 0.0007 | 0.4028 | 0.0204 | 0.0640 | 0.0030 | 285.3 | 4.5 | 343.7 | 14.8 | 742.6 | 100.0 | 81 |
| JF-43-R-1-13 | 168.9 | 212.1 | 0.796 | 0.0428 | 0.0006 | 0.2909 | 0.0126 | 0.0496 | 0.0022 | 270.4 | 3.7 | 259.3 | 9.9 | 176.0 | 100.9 | 95 |
| JF-43-R-1-14 | 90.0 | 80.8 | 1.113 | 0.0423 | 0.0008 | 0.3578 | 0.0236 | 0.0621 | 0.0043 | 267.2 | 4.7 | 310.6 | 17.6 | 677.5 | 150.0 | 84 |
| JF-43-R-1-15 | 217.0 | 219.5 | 0.989 | 0.0640 | 0.0009 | 0.6199 | 0.0353 | 0.0693 | 0.0031 | 399.7 | 5.7 | 489.8 | 22.2 | 907.1 | 92.6 | 79 |
| JF-43-R-1-16 | 383.8 | 501.1 | 0.766 | 0.0695 | 0.0011 | 0.5393 | 0.0175 | 0.0563 | 0.0016 | 433.0 | 6.5 | 438.0 | 11.5 | 464.9 | 30.6 | 98 |
| JF-43-R-1-17 | 160.0 | 509.3 | 0.314 | 0.0747 | 0.0012 | 0.5924 | 0.0182 | 0.0573 | 0.0014 | 464.4 | 7.2 | 472.4 | 11.6 | 505.6 | 58.3 | 98 |
| JF-43-R-1-18 | 376.8 | 563.2 | 0.669 | 0.0672 | 0.0009 | 0.5314 | 0.0134 | 0.0576 | 0.0014 | 419.1 | 5.7 | 432.8 | 8.9 | 522.3 | 53.7 | 96 |
| JF-43-R-1-19 | 228.5 | 579.2 | 0.394 | 0.0737 | 0.0012 | 0.5679 | 0.0160 | 0.0560 | 0.0014 | 458.7 | 7.4 | 456.7 | 10.3 | 450.0 | 55.6 | 99 |
| JF-43-R-1-20 | 345.1 | 692.5 | 0.498 | 0.0698 | 0.0009 | 0.5411 | 0.0126 | 0.0563 | 0.0012 | 435.0 | 5.5 | 439.2 | 8.3 | 464.9 | 46.3 | 99 |
| JF-43-R-1-21 | 212.0 | 722.2 | 0.294 | 0.0643 | 0.0008 | 0.5373 | 0.0144 | 0.0607 | 0.0015 | 401.9 | 5.1 | 436.6 | 9.5 | 631.5 | 53.7 | 91 |
| JF-43-R-1-22 | 449.4 | 1226.0 | 0.367 | 0.0513 | 0.0008 | 0.6060 | 0.0136 | 0.0870 | 0.0024 | 322.3 | 4.8 | 481.1 | 8.6 | 1361.1 | 53.2 | 60 |
| JF-43-R-1-23 | 282.6 | 374.7 | 0.754 | 0.0395 | 0.0006 | 0.2751 | 0.0106 | 0.0510 | 0.0020 | 249.9 | 3.5 | 246.8 | 8.5 | 242.7 | 90.7 | 98 |
| JF-43-R-1-24 | 97.6 | 121.7 | 0.802 | 0.0281 | 0.0006 | 0.3036 | 0.0235 | 0.0777 | 0.0056 | 178.4 | 3.7 | 269.2 | 18.3 | 1140.4 | 144.4 | 59 |
| JF-43-R-1-25 | 370.5 | 1028.9 | 0.360 | 0.2199 | 0.0058 | 3.9289 | 0.1025 | 0.1301 | 0.0017 | 1281.6 | 30.5 | 1619.7 | 21.1 | 2099.7 | 23.9 | 76 |
| JF-43-R-1-26 | 123.3 | 119.7 | 1.030 | 0.0430 | 0.0010 | 0.3562 | 0.0172 | 0.0613 | 0.0030 | 271.3 | 5.9 | 309.4 | 12.9 | 647.9 | 105.5 | 86 |
| JF-43-R-1-27 | 234.9 | 614.9 | 0.382 | 0.0715 | 0.0010 | 0.5424 | 0.0133 | 0.0552 | 0.0012 | 445.0 | 6.3 | 440.0 | 8.7 | 420.4 | 54.6 | 98 |
| JF-43-R-1-28 | 22.6 | 60.2 | 0.375 | 0.0667 | 0.0017 | 1.2279 | 0.0771 | 0.1334 | 0.0080 | 416.5 | 10.4 | 813.4 | 35.2 | 2142.9 | 104.2 | 35 |
| JF-43-R-1-29 | 383.0 | 567.8 | 0.675 | 0.0685 | 0.0009 | 0.5165 | 0.0146 | 0.0547 | 0.0014 | 427.2 | 5.4 | 422.8 | 9.8 | 466.7 | 57.4 | 98 |
| JF-43-R-1-30 | 94.4 | 473.8 | 0.199 | 0.0679 | 0.0010 | 0.5399 | 0.0152 | 0.0576 | 0.0015 | 423.7 | 6.0 | 438.4 | 10.0 | 516.7 | 57.4 | 96 |
| JF-43-R-1-31 | 371.8 | 684.8 | 0.543 | 0.0720 | 0.0013 | 0.5698 | 0.0174 | 0.0574 | 0.0015 | 448.0 | 7.7 | 457.9 | 11.3 | 509.3 | 57.4 | 97 |
| JF-43-R-1-32 | 211.5 | 301.6 | 0.701 | 0.0456 | 0.0007 | 0.3136 | 0.0139 | 0.0496 | 0.0019 | 287.7 | 4.5 | 276.9 | 10.7 | 176.0 | 87.0 | 96 |
| JF-43-R-1-33 | 170.6 | 544.9 | 0.313 | 0.0427 | 0.0006 | 0.3154 | 0.0111 | 0.0534 | 0.0016 | 269.5 | 3.9 | 278.4 | 8.6 | 346.4 | 68.5 | 96 |
| JF-43-R-1-34 | 268.7 | 410.5 | 0.655 | 0.0672 | 0.0008 | 0.6051 | 0.0163 | 0.0657 | 0.0019 | 419.1 | 5.1 | 480.4 | 10.3 | 798.2 | 54.6 | 86 |
| JF-43-R-1-35 | 26.2 | 42.7 | 0.613 | 0.1540 | 0.0026 | 1.5422 | 0.0734 | 0.0736 | 0.0035 | 923.4 | 14.8 | 947.4 | 29.3 | 1031.5 | 96.3 | 97 |
| JF-43-R-1-36 | 513.1 | 545.6 | 0.940 | 0.0406 | 0.0006 | 0.2829 | 0.0095 | 0.0508 | 0.0017 | 256.6 | 3.5 | 252.9 | 7.5 | 231.6 | 77.8 | 98 |
| JF-43-R-1-37 | 272.6 | 308.5 | 0.884 | 0.0403 | 0.0010 | 0.6217 | 0.0671 | 0.1011 | 0.0093 | 254.4 | 6.1 | 490.9 | 42.0 | 1643.5 | 171.4 | 36 |
| JF-43-R-1-38 | 208.6 | 1657.4 | 0.126 | 0.0566 | 0.0015 | 0.4535 | 0.0168 | 0.0573 | 0.0012 | 355.0 | 8.9 | 379.7 | 11.7 | 501.9 | 44.4 | 93 |
| JF-43-R-1-39 | 143.9 | 138.1 | 1.042 | 0.0336 | 0.0012 | 2.2155 | 0.1038 | 0.4759 | 0.0137 | 212.8 | 7.5 | 1186.0 | 32.8 | 4168.4 | 42.6 | -40 |
| JF-43-R-1-40 | 200.5 | 537.8 | 0.373 | 0.0696 | 0.0009 | 0.5262 | 0.0144 | 0.0550 | 0.0014 | 433.7 | 5.6 | 429.3 | 9.6 | 409.3 | 57.4 | 98 |
| JF-43-R-1-41 | 97.8 | 467.9 | 0.209 | 0.0511 | 0.0009 | 0.3767 | 0.0141 | 0.0539 | 0.0020 | 321.0 | 5.7 | 324.6 | 10.4 | 364.9 | 88.0 | 98 |
| JF-43-R-1-42 | 84.3 | 159.5 | 0.529 | 0.3406 | 0.0049 | 5.2660 | 0.1109 | 0.1120 | 0.0018 | 1889.7 | 23.5 | 1863.4 | 18.0 | 1831.8 | 29.0 | 98 |
| JF-43-R-1-43 | 188.6 | 287.6 | 0.656 | 0.0367 | 0.0010 | 1.0144 | 0.0698 | 0.1874 | 0.0109 | 232.1 | 6.3 | 711.1 | 35.2 | 2719.5 | 96.3 | -2 |
| JF-43-R-1-44 | 492.8 | 956.9 | 0.515 | 0.3717 | 0.0039 | 8.3358 | 0.1184 | 0.1626 | 0.0019 | 2037.2 | 18.1 | 2268.2 | 12.9 | 2482.4 | 18.4 | 89 |
| JF-43-R-1-45 | 126.3 | 159.2 | 0.793 | 0.4969 | 0.0077 | 11.6901 | 0.2076 | 0.1708 | 0.0023 | 2600.6 | 33.0 | 2579.9 | 16.6 | 2565.7 | 22.5 | 99 |
| JF-43-R-1-46 | 84.8 | 111.0 | 0.764 | 0.5144 | 0.0072 | 12.2712 | 0.2148 | 0.1733 | 0.0025 | 2675.4 | 30.5 | 2625.4 | 16.4 | 2590.7 | 24.4 | 98 |
| JF-43-R-1-47 | 456.6 | 2775.4 | 0.165 | 0.0731 | 0.0009 | 0.6210 | 0.0115 | 0.0620 | 0.0012 | 454.7 | 5.7 | 490.5 | 7.2 | 672.2 | 42.6 | 92 |
| JF-43-R-1-48 | 54.5 | 129.8 | 0.420 | 0.4474 | 0.0065 | 9.3687 | 0.1798 | 0.1520 | 0.0024 | 2383.6 | 28.9 | 2374.8 | 17.6 | 2368.8 | 26.4 | 99 |
| JF-43-R-1-49 | 123.1 | 156.7 | 0.785 | 0.0436 | 0.0008 | 0.3527 | 0.0203 | 0.0590 | 0.0035 | 275.1 | 4.8 | 306.7 | 15.2 | 568.6 | 132.4 | 89 |
| JF-43-R-1-50 | 499.7 | 2581.5 | 0.194 | 0.0607 | 0.0007 | 0.5539 | 0.0133 | 0.0663 | 0.0017 | 380.1 | 4.1 | 447.5 | 8.7 | 816.7 | 57.6 | 83 |
| JF-43-R-1-51 | 81.8 | 101.9 | 0.802 | 0.4290 | 0.0064 | 8.6861 | 0.1762 | 0.1471 | 0.0027 | 2301.0 | 28.7 | 2305.6 | 18.5 | 2312.7 | 30.1 | 99 |
| JF-43-R-1-52 | 21.6 | 118.4 | 0.182 | 0.3636 | 0.0055 | 6.7447 | 0.1485 | 0.1344 | 0.0023 | 1999.3 | 26.0 | 2078.5 | 19.5 | 2166.7 | 31.0 | 96 |
| JF-43-R-1-53 | 44.6 | 207.3 | 0.215 | 0.3392 | 0.0049 | 5.4502 | 0.1155 | 0.1163 | 0.0019 | 1882.9 | 23.8 | 1892.8 | 18.2 | 1899.7 | 29.6 | 99 |
| JF-43-R-1-54 | 122.3 | 1173.1 | 0.104 | 0.0707 | 0.0013 | 0.5422 | 0.0135 | 0.0558 | 0.0011 | 440.2 | 8.1 | 439.8 | 8.9 | 442.6 | 44.4 | 99 |
| JF-43-R-1-55 | 22.3 | 209.2 | 0.107 | 0.1607 | 0.0025 | 1.5616 | 0.0440 | 0.0702 | 0.0016 | 960.9 | 14.2 | 955.1 | 17.4 | 1000.0 | 47.1 | 99 |
| JF-43-R-1-56 | 366.3 | 285.0 | 1.285 | 0.0279 | 0.0004 | 0.1828 | 0.0109 | 0.0474 | 0.0026 | 177.5 | 2.7 | 170.4 | 9.4 | 77.9 | 116.7 | 95 |
| JF-43-R-1-57 | 100.4 | 230.3 | 0.436 | 0.0605 | 0.0009 | 0.6577 | 0.0289 | 0.0784 | 0.0032 | 378.5 | 5.3 | 513.2 | 17.7 | 1166.7 | 80.1 | 69 |
| JF-43-R-1-58 | 85.7 | 613.9 | 0.140 | 0.3491 | 0.0049 | 5.4499 | 0.0937 | 0.1131 | 0.0016 | 1930.5 | 23.5 | 1892.7 | 14.7 | 1850.3 | 25.5 | 98 |
| JF-43-R-1-59 | 371.8 | 537.9 | 0.691 | 0.0716 | 0.0011 | 0.5590 | 0.0150 | 0.0566 | 0.0014 | 445.7 | 6.4 | 450.9 | 9.8 | 476.0 | 55.6 | 98 |
| JF-43-R-1-60 | 187.9 | 371.5 | 0.506 | 0.0408 | 0.0006 | 0.2990 | 0.0102 | 0.0530 | 0.0017 | 258.0 | 3.5 | 265.6 | 8.0 | 327.8 | 72.2 | 97 |
| JF-43-R-1-61 | 105.3 | 241.2 | 0.437 | 0.1472 | 0.0019 | 1.6028 | 0.0353 | 0.0789 | 0.0017 | 885.2 | 10.7 | 971.3 | 13.8 | 1172.2 | 42.6 | 90 |
| JF-43-R-1-62 | 128.6 | 149.7 | 0.859 | 0.4724 | 0.0065 | 10.8100 | 0.2083 | 0.1657 | 0.0027 | 2494.1 | 28.3 | 2506.9 | 17.9 | 2514.5 | 27.6 | 99 |
| JF-43-R-1-63 | 294.2 | 1456.3 | 0.202 | 0.0717 | 0.0009 | 0.5354 | 0.0107 | 0.0542 | 0.0010 | 446.5 | 5.6 | 435.4 | 7.1 | 388.9 | 38.0 | 97 |
| JF-43-R-1-64 | 239.2 | 769.4 | 0.311 | 0.0678 | 0.0008 | 0.5492 | 0.0134 | 0.0586 | 0.0013 | 422.8 | 4.7 | 444.4 | 8.8 | 553.7 | 54.6 | 94 |
| JF-43-R-1-65 | 55.3 | 127.4 | 0.434 | 0.5313 | 0.0105 | 12.1298 | 0.3078 | 0.1652 | 0.0027 | 2746.7 | 44.3 | 2614.5 | 23.8 | 2510.2 | 27.8 | 95 |
| JF-43-R-1-66 | 112.7 | 179.6 | 0.628 | 0.0713 | 0.0011 | 0.5497 | 0.0188 | 0.0560 | 0.0019 | 444.2 | 6.6 | 444.8 | 12.3 | 453.8 | 75.9 | 99 |
| JF-43-R-1-67 | 140.8 | 251.3 | 0.560 | 0.0693 | 0.0009 | 0.6281 | 0.0189 | 0.0657 | 0.0019 | 431.8 | 5.5 | 494.9 | 11.8 | 796.0 | 54.6 | 86 |
| JF-43-R-1-68 | 69.6 | 316.6 | 0.220 | 0.3526 | 0.0049 | 5.5419 | 0.1033 | 0.1140 | 0.0017 | 1946.9 | 23.1 | 1907.1 | 16.0 | 1864.8 | 27.5 | 97 |
| JF-43-R-1-69 | 140.1 | 295.5 | 0.474 | 0.3856 | 0.0075 | 8.9994 | 0.2187 | 0.1689 | 0.0025 | 2102.4 | 34.7 | 2337.9 | 22.2 | 2547.2 | 25.3 | 89 |
| JF-43-R-1-70 | 98.5 | 445.5 | 0.221 | 0.1691 | 0.0037 | 2.6642 | 0.0762 | 0.1137 | 0.0020 | 1007.3 | 20.1 | 1318.6 | 21.1 | 1861.1 | 31.9 | 73 |
| JF-43-R-1-71 | 410.4 | 449.9 | 0.912 | 0.1638 | 0.0024 | 1.6498 | 0.0383 | 0.0731 | 0.0015 | 977.8 | 13.3 | 989.5 | 14.7 | 1016.7 | 40.7 | 98 |
| JF-43-R-1-72 | 132.2 | 218.5 | 0.605 | 0.0354 | 0.0007 | 0.2288 | 0.0121 | 0.0475 | 0.0024 | 224.2 | 4.3 | 209.2 | 10.0 | 76.0 | 118.5 | 93 |
| JF-43-R-1-73 | 144.4 | 183.8 | 0.786 | 0.4806 | 0.0065 | 10.6621 | 0.1906 | 0.1610 | 0.0023 | 2529.7 | 28.4 | 2494.1 | 16.6 | 2466.4 | 24.2 | 98 |
| JF-43-R-1-74 | 64.0 | 936.9 | 0.068 | 0.0387 | 0.0006 | 0.2855 | 0.0088 | 0.0534 | 0.0013 | 244.7 | 3.8 | 255.0 | 6.9 | 346.4 | 89.8 | 95 |
| JF-43-R-1-75 | 326.0 | 515.2 | 0.633 | 0.0737 | 0.0012 | 0.5478 | 0.0142 | 0.0542 | 0.0013 | 458.4 | 7.5 | 443.6 | 9.3 | 388.9 | 55.6 | 96 |
| JF-43-R-1-76 | 170.1 | 342.8 | 0.496 | 0.0384 | 0.0005 | 0.2534 | 0.0096 | 0.0479 | 0.0017 | 242.9 | 3.4 | 229.4 | 7.7 | 100.1 | 85.2 | 94 |
| JF-43-R-1-77 | 46.8 | 82.4 | 0.569 | 0.1934 | 0.0028 | 2.8692 | 0.4810 | 0.1063 | 0.0166 | 1139.9 | 15.2 | 1373.9 | 126.9 | 1736.7 | 289.0 | 81 |
| JF-43-R-1-78 | 122.4 | 181.3 | 0.675 | 0.1402 | 0.0021 | 1.5143 | 0.0476 | 0.0789 | 0.0026 | 845.8 | 11.6 | 936.2 | 19.2 | 1169.4 | 63.4 | 89 |
| JF-43-R-1-79 | 99.7 | 418.8 | 0.238 | 0.1081 | 0.0016 | 0.9616 | 0.0222 | 0.0646 | 0.0013 | 661.7 | 9.2 | 684.1 | 11.5 | 761.1 | 43.7 | 96 |
| JF-43-R-1-80 | 110.5 | 244.5 | 0.452 | 0.4551 | 0.0076 | 10.1566 | 0.2268 | 0.1615 | 0.0025 | 2417.9 | 33.8 | 2449.1 | 20.6 | 2472.2 | 21.3 | 98 |
| JF-43-R-1-81 | 277.2 | 638.2 | 0.434 | 0.0701 | 0.0010 | 0.6230 | 0.0188 | 0.0643 | 0.0018 | 436.6 | 5.8 | 491.7 | 11.8 | 753.7 | 57.4 | 88 |
| JF-43-R-1-82 | 99.7 | 324.1 | 0.308 | 0.1343 | 0.0019 | 1.2417 | 0.0338 | 0.0670 | 0.0017 | 812.1 | 10.6 | 819.7 | 15.3 | 838.9 | 51.8 | 99 |
| JF-43-R-1-83 | 448.5 | 458.5 | 0.978 | 0.0668 | 0.0011 | 0.5335 | 0.0172 | 0.0583 | 0.0018 | 416.7 | 6.7 | 434.2 | 11.4 | 538.9 | 63.9 | 95 |
| JF-43-R-1-84 | 448.3 | 569.9 | 0.787 | 0.0174 | 0.0003 | 0.1769 | 0.0092 | 0.0747 | 0.0039 | 110.9 | 1.9 | 165.3 | 7.9 | 1061.1 | 107.4 | 60 |
| JF-43-R-1-85 | 123.7 | 984.8 | 0.126 | 0.2897 | 0.0110 | 5.4415 | 0.1567 | 0.1427 | 0.0034 | 1640.2 | 55.0 | 1891.4 | 24.7 | 2261.1 | 41.8 | 85 |
| JF-43-R-1-86 | 51.4 | 37.5 | 1.371 | 0.4225 | 0.0106 | 9.2427 | 0.3217 | 0.1581 | 0.0037 | 2271.9 | 48.0 | 2362.4 | 31.9 | 2435.5 | 40.1 | 96 |
| JF-43-R-1-87 | 353.6 | 642.9 | 0.550 | 0.1797 | 0.0028 | 1.9685 | 0.0387 | 0.0794 | 0.0013 | 1065.6 | 15.5 | 1104.8 | 13.2 | 1181.2 | 31.5 | 96 |
| JF-43-R-1-88 | 364.1 | 744.0 | 0.489 | 0.0784 | 0.0015 | 0.7093 | 0.0236 | 0.0649 | 0.0015 | 486.3 | 8.7 | 544.3 | 14.0 | 772.2 | 49.2 | 88 |
| JF-43-R-1-89 | 79.7 | 129.6 | 0.615 | 0.1186 | 0.0024 | 1.1040 | 0.0409 | 0.0676 | 0.0022 | 722.5 | 13.7 | 755.3 | 19.7 | 857.4 | 67.4 | 95 |
| JF-43-R-1-90 | 260.8 | 1119.9 | 0.233 | 0.0721 | 0.0010 | 0.5639 | 0.0141 | 0.0567 | 0.0013 | 449.0 | 6.2 | 454.1 | 9.1 | 479.7 | 50.0 | 98 |
| JF-43-R-1-91 | 94.3 | 368.0 | 0.256 | 0.1439 | 0.0034 | 1.3942 | 0.0479 | 0.0695 | 0.0014 | 866.9 | 19.4 | 886.5 | 20.3 | 922.2 | 48.3 | 97 |
| JF-43-R-1-92 | 980.1 | 1362.8 | 0.719 | 0.0433 | 0.0006 | 0.3695 | 0.0122 | 0.0617 | 0.0019 | 273.2 | 3.9 | 319.3 | 9.0 | 664.8 | 64.8 | 84 |
| JF-43-R-1-93 | 182.2 | 262.4 | 0.695 | 0.0434 | 0.0007 | 0.4630 | 0.0206 | 0.0770 | 0.0032 | 273.7 | 4.1 | 386.3 | 14.3 | 1121.3 | 83.8 | 65 |
| JF-43-R-1-94 | 98.6 | 119.8 | 0.823 | 0.0646 | 0.0011 | 0.4596 | 0.0254 | 0.0513 | 0.0027 | 403.8 | 6.6 | 383.9 | 17.7 | 253.8 | 120.4 | 94 |
| JF-43-R-1-95 | 288.0 | 634.5 | 0.454 | 0.4749 | 0.0069 | 10.5758 | 0.1761 | 0.1611 | 0.0019 | 2504.9 | 30.0 | 2486.6 | 15.4 | 2477.8 | 19.8 | 99 |
| JF-43-R-1-96 | 63.8 | 58.2 | 1.096 | 0.4501 | 0.0088 | 10.0427 | 0.2501 | 0.1615 | 0.0029 | 2395.9 | 39.2 | 2438.7 | 23.0 | 2471.9 | 30.9 | 98 |
| JF-43-R-1-97 | 181.2 | 397.2 | 0.456 | 0.2608 | 0.0077 | 3.9025 | 0.1299 | 0.1079 | 0.0017 | 1493.7 | 39.3 | 1614.2 | 26.9 | 1764.8 | 29.5 | 92 |
| JF-43-R-1-98 | 78.6 | 203.4 | 0.386 | 0.3180 | 0.0047 | 4.9558 | 0.1060 | 0.1125 | 0.0019 | 1779.8 | 22.9 | 1811.8 | 18.1 | 1842.6 | 31.0 | 98 |
| JF-43-R-1-99 | 35.1 | 174.1 | 0.202 | 0.3220 | 0.0068 | 4.9354 | 0.1244 | 0.1110 | 0.0020 | 1799.5 | 32.9 | 1808.3 | 21.3 | 1816.7 | 33.3 | 99 |
| JF-43-R-1-100 | 81.3 | 1215.8 | 0.067 | 0.4016 | 0.0492 | 8.1334 | 1.1393 | 0.1403 | 0.0034 | 2176.5 | 226.4 | 2246.0 | 127.3 | 2231.5 | 42.3 | 96 |
| JF-43-R-1-101 | 1664.2 | 1953.7 | 0.852 | 0.1064 | 0.0017 | 1.1090 | 0.0201 | 0.0756 | 0.0012 | 651.6 | 9.7 | 757.7 | 9.7 | 1084.3 | 32.9 | 84 |
| JF-43-R-1-102 | 320.7 | 967.3 | 0.332 | 0.0383 | 0.0005 | 0.2746 | 0.0074 | 0.0518 | 0.0013 | 242.3 | 3.0 | 246.4 | 5.9 | 279.7 | 62.0 | 98 |
| JF-43-R-1-103 | 276.8 | 2844.3 | 0.097 | 0.0650 | 0.0010 | 0.4909 | 0.0101 | 0.0546 | 0.0008 | 405.9 | 6.2 | 405.6 | 6.9 | 398.2 | 35.2 | 99 |
| JF-43-R-1-104 | 144.2 | 860.6 | 0.168 | 0.0757 | 0.0011 | 0.5526 | 0.0120 | 0.0529 | 0.0010 | 470.2 | 6.3 | 446.7 | 7.9 | 324.1 | 44.4 | 94 |
| JF-43-R-1-105 | 677.4 | 2149.3 | 0.315 | 0.1615 | 0.0019 | 3.3311 | 0.0625 | 0.1491 | 0.0022 | 965.0 | 10.5 | 1488.4 | 14.7 | 2334.9 | 25.8 | 57 |
| JF-43-R-1-106 | 38.8 | 102.7 | 0.377 | 0.5082 | 0.0076 | 11.6258 | 0.2167 | 0.1660 | 0.0026 | 2648.8 | 32.6 | 2574.8 | 17.4 | 2518.2 | 25.6 | 97 |
| JF-43-R-1-107 | 57.0 | 173.2 | 0.329 | 0.3903 | 0.0053 | 6.5585 | 0.1332 | 0.1217 | 0.0020 | 2124.4 | 24.4 | 2053.8 | 17.9 | 1983.3 | 30.7 | 96 |
| JF-43-R-1-108 | 86.1 | 1273.3 | 0.068 | 0.0729 | 0.0009 | 0.5551 | 0.0117 | 0.0552 | 0.0010 | 453.5 | 5.6 | 448.3 | 7.7 | 420.4 | 36.1 | 98 |
| JF-43-R-1-109 | 165.0 | 1005.8 | 0.164 | 0.1744 | 0.0031 | 2.1034 | 0.0516 | 0.0871 | 0.0013 | 1036.2 | 17.2 | 1149.9 | 16.9 | 1362.7 | -4.6 | 89 |
| JF-43-R-1-110 | 87.2 | 83.4 | 1.046 | 0.1503 | 0.0042 | 4.3787 | 0.4281 | 0.1916 | 0.0136 | 902.5 | 23.7 | 1708.3 | 81.0 | 2766.7 | 116.2 | 38 |

Supplementary Table 2. Detrital zircon trace element compositions of the sedimentary rocks from the Early-Middle Triassic in the study region.

| Site number | | La | | Ce | | Pr | | Nd | | | Sm | | | Eu | | | Gd | | | Tb | | | Dy | | Ho | | Er | | Tm | | Yb | | Lu | | Y | | Nb | | Hf | | Ta | |
| --- | --- | --- | --- | --- | --- | --- | --- | --- | --- | --- | --- | --- | --- | --- | --- | --- | --- | --- | --- | --- | --- | --- | --- | --- | --- | --- | --- | --- | --- | --- | --- | --- | --- | --- | --- | --- | --- | --- | --- | --- | --- | --- |
| QJP-39-R-1 | |  | |  | |  | |  | | |  | | |  | | |  | | |  | | |  | |  | |  | |  | |  | |  | |  | |  | |  | |  | |
| QJP-39-R-1-01 | 18.70 | | 89.66 | | 10.92 | | 63.96 | | 39.57 | | | 13.38 | | | 83.68 | | | 22.95 | | | 225.22 | | | 67.49 | | 267.89 | | 57.67 | | 554.26 | | 95.41 | | 1976.69 | | 1.68 | | 10664.67 | | 0.71 | |  |
| QJP-39-R-1-02 | 0.96 | | 18.14 | | 1.35 | | 9.64 | | 10.33 | | | 3.97 | | | 28.48 | | | 9.70 | | | 103.75 | | | 31.69 | | 127.06 | | 26.67 | | 230.76 | | 38.56 | | 892.32 | | 2.47 | | 12694.54 | | 1.25 | |  |
| QJP-39-R-1-03 | 78.79 | | 214.59 | | 54.81 | | 284.21 | | 147.07 | | | 47.03 | | | 220.62 | | | 58.29 | | | 504.97 | | | 122.25 | | 437.23 | | 91.79 | | 822.88 | | 130.00 | | 3371.13 | | 0.86 | | 12726.77 | | 1.01 | |  |
| QJP-39-R-1-04 | 10.47 | | 126.05 | | 20.79 | | 135.21 | | 76.69 | | | 19.15 | | | 140.34 | | | 38.37 | | | 358.40 | | | 102.99 | | 405.59 | | 81.37 | | 727.65 | | 117.48 | | 2959.98 | | 1.92 | | 14835.84 | | 1.60 | |  |
| QJP-39-R-1-05 | 0.04 | | 15.01 | | 0.04 | | 0.40 | | 1.11 | | | 0.14 | | | 7.36 | | | 2.79 | | | 33.96 | | | 13.61 | | 66.36 | | 15.75 | | 157.99 | | 31.47 | | 437.78 | | 2.43 | | 12880.98 | | 1.81 | |  |
| QJP-39-R-1-06 | 0.00 | | 1.98 | | 0.23 | | 3.79 | | 8.15 | | | 0.15 | | | 29.57 | | | 8.08 | | | 76.39 | | | 22.41 | | 80.69 | | 15.16 | | 116.35 | | 18.84 | | 645.01 | | 0.61 | | 13262.07 | | 0.28 | |  |
| QJP-39-R-1-07 | 74.42 | | 249.19 | | 52.96 | | 291.04 | | 164.14 | | | 51.55 | | | 294.98 | | | 85.14 | | | 804.94 | | | 220.04 | | 851.93 | | 176.45 | | 1623.33 | | 260.75 | | 6283.27 | | 22.85 | | 11836.87 | | 8.94 | |  |
| QJP-39-R-1-08 | 54.95 | | 176.09 | | 14.93 | | 74.35 | | 22.49 | | | 1.26 | | | 65.35 | | | 18.40 | | | 214.63 | | | 81.21 | | 362.18 | | 78.28 | | 726.71 | | 130.00 | | 2407.87 | | 3.63 | | 9139.25 | | 1.17 | |  |
| QJP-39-R-1-09 | 43.20 | | 360.03 | | 31.30 | | 167.24 | | 98.84 | | | 32.91 | | | 170.90 | | | 44.90 | | | 391.02 | | | 98.49 | | 354.92 | | 76.29 | | 658.77 | | 104.04 | | 2978.18 | | 11.86 | | 7711.58 | | 0.97 | |  |
| QJP-39-R-1-10 | 20.18 | | 139.69 | | 12.95 | | 70.01 | | 36.59 | | | 9.79 | | | 63.28 | | | 18.34 | | | 184.29 | | | 61.92 | | 276.94 | | 64.90 | | 647.17 | | 114.50 | | 1925.31 | | 6.04 | | 11842.11 | | 3.25 | |  |
| QJP-39-R-1-11 | 10.67 | | 48.39 | | 5.74 | | 32.44 | | 17.35 | | | 4.16 | | | 40.31 | | | 10.92 | | | 114.72 | | | 39.17 | | 165.03 | | 34.95 | | 328.18 | | 56.97 | | 1153.91 | | 4.40 | | 12299.08 | | 2.10 | |  |
| QJP-39-R-1-12 | 0.84 | | 5.63 | | 0.56 | | 3.60 | | 4.32 | | | 0.51 | | | 28.63 | | | 10.39 | | | 151.74 | | | 59.81 | | 291.60 | | 66.23 | | 629.26 | | 114.22 | | 1823.45 | | 4.02 | | 13792.69 | | 3.64 | |  |
| QJP-39-R-1-13 | 20.46 | | 136.15 | | 5.13 | | 22.21 | | 10.48 | | | 2.38 | | | 41.17 | | | 13.50 | | | 163.14 | | | 61.75 | | 290.92 | | 64.81 | | 624.57 | | 116.05 | | 1927.44 | | 7.12 | | 10143.97 | | 2.09 | |  |
| QJP-39-R-1-14 | 0.06 | | 13.37 | | 0.09 | | 1.10 | | 2.45 | | | 0.21 | | | 14.59 | | | 5.33 | | | 63.20 | | | 23.70 | | 117.08 | | 27.36 | | 269.28 | | 48.63 | | 771.03 | | 3.23 | | 13829.80 | | 2.02 | |  |
| QJP-39-R-1-15 | 72.08 | | 242.83 | | 33.58 | | 185.90 | | 78.69 | | | 20.45 | | | 133.17 | | | 35.79 | | | 343.99 | | | 102.78 | | 423.01 | | 82.97 | | 754.18 | | 132.51 | | 2977.22 | | 2.84 | | 10281.04 | | 1.31 | |  |
| QJP-39-R-1-16 | 13.54 | | 55.39 | | 9.51 | | 52.12 | | 30.52 | | | 9.65 | | | 56.10 | | | 15.24 | | | 156.86 | | | 48.08 | | 194.52 | | 44.56 | | 433.49 | | 81.92 | | 1549.44 | | 2.65 | | 11164.31 | | 0.55 | |  |
| QJP-39-R-1-17 | 65.25 | | 205.38 | | 47.48 | | 253.82 | | 130.37 | | | 37.09 | | | 212.36 | | | 55.65 | | | 572.26 | | | 148.40 | | 579.07 | | 128.13 | | 1198.38 | | 218.72 | | 4237.20 | | 10.85 | | 18339.85 | | 33.47 | |  |
| QJP-39-R-1-18 | 0.13 | | 28.15 | | 0.37 | | 3.81 | | 7.12 | | | 1.35 | | | 26.10 | | | 6.95 | | | 76.75 | | | 26.65 | | 111.06 | | 22.86 | | 225.88 | | 42.77 | | 767.20 | | 1.61 | | 8740.26 | | 0.39 | |  |
| QJP-39-R-1-19 | 2.82 | | 13.72 | | 2.41 | | 12.44 | | 5.91 | | | 1.82 | | | 19.07 | | | 5.45 | | | 55.83 | | | 21.00 | | 91.37 | | 22.76 | | 237.49 | | 46.02 | | 590.62 | | 1.66 | | 12958.11 | | 0.79 | |  |
| QJP-39-R-1-20 | 0.30 | | 2.34 | | 0.00 | | 0.58 | | 0.34 | | | 0.00 | | | 3.38 | | | 0.85 | | | 11.93 | | | 5.08 | | 26.89 | | 6.93 | | 82.38 | | 21.21 | | 172.28 | | 0.64 | | 11886.03 | | 0.45 | |  |
| QJP-39-R-1-21 | 0.00 | | 22.24 | | 0.22 | | 3.38 | | 7.23 | | | 1.50 | | | 37.22 | | | 10.82 | | | 135.84 | | | 53.83 | | 275.87 | | 63.55 | | 618.52 | | 129.50 | | 1834.13 | | 1.57 | | 10153.31 | | 0.47 | |  |
| QJP-39-R-1-22 | 0.04 | | 7.59 | | 0.00 | | 0.66 | | 0.66 | | | 0.06 | | | 4.97 | | | 1.58 | | | 28.66 | | | 11.63 | | 64.71 | | 16.53 | | 176.06 | | 38.12 | | 409.77 | | 1.15 | | 11927.10 | | 1.03 | |  |
| QJP-39-R-1-23 | 0.57 | | 10.98 | | 2.09 | | 26.84 | | 33.82 | | | 2.30 | | | 165.27 | | | 45.83 | | | 483.13 | | | 174.61 | | 772.16 | | 160.86 | | 1344.46 | | 237.53 | | 5087.91 | | 2.05 | | 9481.40 | | 0.75 | |  |
| QJP-39-R-1-24 | 20.27 | | 92.43 | | 14.36 | | 79.93 | | 39.52 | | | 11.37 | | | 100.45 | | | 27.28 | | | 273.81 | | | 76.41 | | 352.52 | | 66.43 | | 574.43 | | 106.44 | | 2318.70 | | 1.21 | | 8377.12 | | 0.34 | |  |
| QJP-39-R-1-25 | 162.33 | | 840.40 | | 128.53 | | 727.20 | | 394.03 | | | 128.16 | | | 713.69 | | | 202.96 | | | 1950.51 | | | 506.54 | | 1992.93 | | 392.40 | | 3544.23 | | 508.81 | | 13386.04 | | 4.62 | | 12101.58 | | 2.05 | |  |
| QJP-39-R-1-26 | 14.06 | | 54.77 | | 9.20 | | 61.04 | | 30.96 | | | 10.41 | | | 79.01 | | | 21.56 | | | 219.66 | | | 80.67 | | 354.62 | | 69.88 | | 653.99 | | 132.05 | | 2377.67 | | 3.96 | | 12216.30 | | 1.65 | |  |
| QJP-39-R-1-27 | 0.19 | | 16.82 | | 0.15 | | 1.60 | | 3.48 | | | 0.00 | | | 23.52 | | | 8.54 | | | 111.12 | | | 43.20 | | 210.95 | | 45.21 | | 442.26 | | 83.92 | | 1337.64 | | 3.30 | | 9028.21 | | 1.88 | |  |
| QJP-39-R-1-28 | 0.60 | | 28.10 | | 0.13 | | 2.15 | | 2.28 | | | 1.49 | | | 19.04 | | | 5.64 | | | 58.90 | | | 21.36 | | 107.62 | | 23.05 | | 236.90 | | 49.36 | | 743.71 | | 1.58 | | 8887.23 | | 0.34 | |  |
| QJP-39-R-1-29 | 6.42 | | 45.09 | | 5.34 | | 47.73 | | 16.86 | | | 3.49 | | | 41.71 | | | 11.54 | | | 126.77 | | | 44.41 | | 204.08 | | 39.28 | | 397.43 | | 77.50 | | 1312.00 | | 2.82 | | 9025.74 | | 1.64 | |  |
| QJP-39-R-1-30 | 74.93 | | 563.09 | | 63.78 | | 333.97 | | 179.38 | | | 56.70 | | | 344.64 | | | 102.65 | | | 905.77 | | | 245.70 | | 888.59 | | 169.27 | | 1395.22 | | 272.72 | | 6646.30 | | 3.79 | | 9360.91 | | 1.31 | |  |
| QJP-39-R-1-31 | 0.12 | | 16.27 | | 0.04 | | 0.16 | | 1.14 | | | 0.15 | | | 10.31 | | | 2.75 | | | 37.24 | | | 13.28 | | 65.00 | | 14.36 | | 134.51 | | 25.38 | | 433.43 | | 2.68 | | 7285.59 | | 1.79 | |  |
| QJP-39-R-1-32 | 7.53 | | 44.96 | | 4.21 | | 24.85 | | 13.23 | | | 4.10 | | | 24.38 | | | 7.35 | | | 65.57 | | | 22.60 | | 97.85 | | 24.19 | | 241.34 | | 48.36 | | 774.34 | | 1.41 | | 9097.90 | | 0.65 | |  |
| QJP-39-R-1-33 | 0.00 | | 3.16 | | 0.16 | | 3.22 | | 6.98 | | | 0.48 | | | 38.48 | | | 11.03 | | | 145.09 | | | 51.42 | | 241.69 | | 48.51 | | 470.65 | | 83.89 | | 1645.17 | | 1.42 | | 8498.31 | | 0.70 | |  |
| QJP-39-R-1-34 | 0.00 | | 21.32 | | 0.12 | | 0.74 | | 0.77 | | | 0.12 | | | 7.35 | | | 2.07 | | | 19.95 | | | 7.83 | | 37.90 | | 9.62 | | 110.67 | | 20.33 | | 270.46 | | 1.20 | | 10129.25 | | 2.03 | |  |
| QJP-39-R-1-35 | 10.81 | | 67.11 | | 7.30 | | 37.10 | | 20.08 | | | 6.11 | | | 36.17 | | | 12.04 | | | 129.61 | | | 39.48 | | 185.89 | | 45.87 | | 481.90 | | 99.84 | | 1403.63 | | 3.59 | | 8965.63 | | 0.83 | |  |
| QJP-39-R-1-36 | 28.30 | | 104.21 | | 16.87 | | 83.82 | | 47.88 | | | 11.76 | | | 85.82 | | | 20.76 | | | 206.58 | | | 47.33 | | 188.13 | | 45.15 | | 436.15 | | 69.98 | | 1631.52 | | 3.24 | | 10325.35 | | 0.58 | |  |
| QJP-39-R-1-37 | 0.24 | | 6.17 | | 0.82 | | 7.73 | | 11.44 | | | 0.79 | | | 63.01 | | | 23.84 | | | 292.95 | | | 99.38 | | 391.61 | | 71.08 | | 572.56 | | 84.59 | | 3127.88 | | 2.27 | | 9971.85 | | 0.84 | |  |
| QJP-39-R-1-38 | 1.53 | | 17.99 | | 1.00 | | 4.75 | | 6.24 | | | 0.91 | | | 34.22 | | | 10.10 | | | 126.08 | | | 44.28 | | 184.33 | | 34.65 | | 328.50 | | 58.48 | | 1252.48 | | 7.92 | | 7126.04 | | 3.00 | |  |
| QJP-39-R-1-39 | 0.45 | | 10.67 | | 0.35 | | 2.05 | | 1.31 | | | 0.30 | | | 4.49 | | | 2.68 | | | 32.20 | | | 12.19 | | 59.65 | | 12.96 | | 149.63 | | 28.22 | | 401.41 | | 1.33 | | 8255.51 | | 0.47 | |  |
| QJP-39-R-1-40 | 15.76 | | 47.65 | | 3.95 | | 14.15 | | 5.62 | | | 2.49 | | | 19.59 | | | 7.52 | | | 75.81 | | | 31.60 | | 162.84 | | 39.24 | | 433.26 | | 87.72 | | 1068.23 | | 2.11 | | 6540.10 | | 0.81 | |  |
| QJP-39-R-1-41 | 0.08 | | 60.35 | | 0.53 | | 9.94 | | 13.22 | | | 3.03 | | | 52.82 | | | 12.97 | | | 143.62 | | | 45.01 | | 188.22 | | 35.99 | | 327.13 | | 54.55 | | 1316.94 | | 0.82 | | 7038.71 | | 0.35 | |  |
| QJP-39-R-1-42 | 5.85 | | 51.32 | | 4.25 | | 23.43 | | 13.76 | | | 4.27 | | | 22.47 | | | 6.48 | | | 71.83 | | | 23.14 | | 104.82 | | 25.53 | | 268.65 | | 48.85 | | 699.54 | | 1.69 | | 10097.66 | | 1.56 | |  |
| QJP-39-R-1-43 | 42.62 | | 156.76 | | 31.35 | | 155.83 | | 87.79 | | | 25.72 | | | 128.86 | | | 34.25 | | | 304.63 | | | 73.41 | | 307.61 | | 66.75 | | 584.95 | | 100.78 | | 2277.43 | | 2.92 | | 10613.75 | | 2.41 | |  |
| QJP-39-R-1-44 | 0.02 | | 8.88 | | 0.17 | | 1.86 | | 3.88 | | | 1.24 | | | 20.15 | | | 6.97 | | | 80.67 | | | 29.91 | | 136.09 | | 30.86 | | 300.84 | | 55.93 | | 974.24 | | 0.73 | | 6738.46 | | 0.24 | |  |
| QJP-39-R-1-45 | 12.47 | | 53.56 | | 8.24 | | 45.03 | | 26.06 | | | 9.13 | | | 53.35 | | | 15.34 | | | 148.12 | | | 43.63 | | 176.06 | | 37.34 | | 347.69 | | 53.26 | | 1297.74 | | 8.09 | | 9400.70 | | 2.22 | |  |
| QJP-39-R-1-46 | 0.93 | | 19.12 | | 0.37 | | 2.42 | | 1.54 | | | 0.64 | | | 5.28 | | | 1.94 | | | 23.71 | | | 9.60 | | 49.70 | | 12.53 | | 138.16 | | 27.83 | | 334.19 | | 0.89 | | 10230.38 | | 0.88 | |  |
| QJP-39-R-1-47 | 0.13 | | 25.36 | | 0.17 | | 1.39 | | 2.87 | | | 1.70 | | | 9.34 | | | 3.48 | | | 34.36 | | | 13.20 | | 63.66 | | 14.94 | | 151.76 | | 29.95 | | 421.50 | | 1.21 | | 8980.61 | | 0.30 | |  |
| QJP-39-R-1-48 | 0.00 | | 28.61 | | 0.17 | | 3.63 | | 8.67 | | | 1.45 | | | 47.31 | | | 16.67 | | | 229.19 | | | 86.02 | | 415.82 | | 91.42 | | 879.83 | | 158.16 | | 2773.41 | | 3.93 | | 9283.53 | | 1.55 | |  |
| QJP-39-R-1-49 | 309.56 | | 666.23 | | 66.97 | | 273.64 | | 43.27 | | | 4.28 | | | 43.82 | | | 6.85 | | | 62.02 | | | 19.76 | | 87.34 | | 19.26 | | 182.17 | | 33.44 | | 633.48 | | 2.90 | | 9234.83 | | 1.41 | |  |
| QJP-39-R-1-50 | 5.94 | | 24.46 | | 3.86 | | 18.18 | | 8.46 | | | 2.80 | | | 16.21 | | | 4.50 | | | 45.46 | | | 13.96 | | 68.27 | | 16.37 | | 172.85 | | 34.78 | | 476.83 | | 1.31 | | 10626.16 | | 1.15 | |  |
| QJP-39-R-1-51 | 4.17 | | 16.09 | | 1.62 | | 10.40 | | 8.47 | | | 0.49 | | | 36.96 | | | 11.29 | | | 135.28 | | | 51.77 | | 239.10 | | 49.88 | | 475.17 | | 81.80 | | 1562.26 | | 8.59 | | 6715.21 | | 1.08 | |  |
| QJP-39-R-1-52 | 178.38 | | 518.61 | | 130.33 | | 702.61 | | 396.93 | | | 127.67 | | | 708.04 | | | 193.76 | | | 1799.23 | | | 463.98 | | 1731.06 | | 340.66 | | 2910.57 | | 446.04 | | 12359.90 | | 27.49 | | 9230.28 | | 6.60 | |  |
| QJP-39-R-1-53 | 41.55 | | 188.97 | | 34.24 | | 165.73 | | 101.59 | | | 33.62 | | | 200.07 | | | 61.55 | | | 625.31 | | | 172.90 | | 744.40 | | 156.79 | | 1408.09 | | 236.64 | | 4374.21 | | 21.75 | | 10033.38 | | 7.27 | |  |
| QJP-39-R-1-54 | 68.94 | | 289.43 | | 41.56 | | 210.86 | | 104.69 | | | 29.67 | | | 162.39 | | | 38.98 | | | 337.44 | | | 84.33 | | 314.41 | | 62.91 | | 605.73 | | 99.03 | | 2545.94 | | 12.59 | | 8937.64 | | 2.65 | |  |
| QJP-39-R-1-56 | 0.00 | | 7.11 | | 0.54 | | 9.25 | | 13.12 | | | 0.69 | | | 57.03 | | | 16.80 | | | 200.72 | | | 69.23 | | 301.44 | | 57.22 | | 511.16 | | 83.83 | | 2073.84 | | 2.06 | | 8707.01 | | 0.92 | |  |
| QJP-39-R-1-57 | 0.23 | | 60.78 | | 1.42 | | 20.08 | | 22.72 | | | 8.18 | | | 65.01 | | | 15.56 | | | 146.00 | | | 41.97 | | 174.85 | | 33.15 | | 303.61 | | 48.86 | | 1326.61 | | 1.11 | | 7723.47 | | 0.37 | |  |
| QJP-39-R-1-58 | 0.00 | | 24.05 | | 0.02 | | 0.15 | | 1.29 | | | 0.21 | | | 6.55 | | | 2.58 | | | 33.75 | | | 15.12 | | 85.12 | | 20.91 | | 243.15 | | 51.23 | | 538.72 | | 2.31 | | 10910.09 | | 0.85 | |  |
| QJP-39-R-1-59 | 7.40 | | 84.64 | | 5.53 | | 31.49 | | 18.82 | | | 6.84 | | | 49.89 | | | 14.30 | | | 141.97 | | | 43.24 | | 166.47 | | 34.53 | | 335.18 | | 54.26 | | 1213.14 | | 2.08 | | 11296.68 | | 2.59 | |  |
| QJP-39-R-1-60 | 26.87 | | 92.40 | | 17.88 | | 98.96 | | 50.45 | | | 16.98 | | | 100.68 | | | 27.31 | | | 281.02 | | | 78.64 | | 326.03 | | 69.54 | | 666.18 | | 110.08 | | 2415.76 | | 8.25 | | 10818.05 | | 3.51 | |  |
| QJP-39-R-1-61 | 41.01 | | 119.23 | | 12.03 | | 57.33 | | 16.77 | | | 0.37 | | | 39.14 | | | 10.96 | | | 122.33 | | | 43.33 | | 190.81 | | 37.99 | | 363.75 | | 60.40 | | 1293.69 | | 4.17 | | 8662.91 | | 1.30 | |  |
| QJP-39-R-1-62 | 26.12 | | 83.14 | | 17.23 | | 86.99 | | 41.94 | | | 11.91 | | | 60.47 | | | 15.84 | | | 135.56 | | | 35.74 | | 142.05 | | 32.12 | | 326.77 | | 54.76 | | 1183.27 | | 1.35 | | 10067.94 | | 1.84 | |  |
| QJP-39-R-1-63 | 1.48 | | 16.87 | | 0.39 | | 2.25 | | 2.35 | | | 0.34 | | | 10.04 | | | 4.24 | | | 46.87 | | | 18.08 | | 74.92 | | 16.21 | | 156.39 | | 29.41 | | 548.89 | | 1.96 | | 9445.29 | | 0.78 | |  |
| QJP-39-R-1-64 | 0.00 | | 12.73 | | 0.09 | | 0.68 | | 0.71 | | | 0.40 | | | 3.92 | | | 1.09 | | | 14.63 | | | 5.81 | | 28.20 | | 7.03 | | 83.71 | | 17.71 | | 193.96 | | 0.65 | | 10153.10 | | 0.52 | |  |
| QJP-39-R-1-65 | 8.37 | | 61.45 | | 6.45 | | 35.88 | | 19.68 | | | 7.43 | | | 42.89 | | | 11.96 | | | 105.59 | | | 28.56 | | 115.52 | | 22.02 | | 233.39 | | 38.95 | | 837.56 | | 0.72 | | 11495.70 | | 0.59 | |  |
| QJP-39-R-1-66 | 0.18 | | 2.82 | | 0.42 | | 3.26 | | 7.34 | | | 0.63 | | | 42.54 | | | 14.84 | | | 153.55 | | | 48.34 | | 178.97 | | 32.32 | | 285.22 | | 45.72 | | 1492.01 | | 0.52 | | 11347.05 | | 0.24 | |  |
| QJP-39-R-1-67 | 28.43 | | 125.98 | | 13.62 | | 68.83 | | 42.43 | | | 8.35 | | | 151.40 | | | 46.46 | | | 521.56 | | | 179.36 | | 754.68 | | 157.29 | | 1464.97 | | 246.93 | | 3579.94 | | 16.16 | | 8916.21 | | 3.81 | |  |
| QJP-39-R-1-68 | 0.74 | | 28.30 | | 0.65 | | 5.64 | | 9.60 | | | 1.16 | | | 53.31 | | | 17.77 | | | 214.32 | | | 78.58 | | 335.21 | | 67.43 | | 636.69 | | 104.53 | | 2219.91 | | 2.96 | | 8409.09 | | 1.13 | |  |
| QJP-39-R-1-69 | 10.08 | | 36.62 | | 3.91 | | 26.49 | | 19.64 | | | 5.19 | | | 68.89 | | | 19.15 | | | 211.59 | | | 71.77 | | 287.80 | | 57.39 | | 520.68 | | 88.95 | | 2013.16 | | 1.24 | | 7517.18 | | 0.41 | |  |
| QJP-39-R-1-70 | 74.75 | | 185.24 | | 19.24 | | 87.42 | | 21.01 | | | 2.11 | | | 35.63 | | | 9.13 | | | 89.80 | | | 29.77 | | 136.73 | | 28.50 | | 272.70 | | 48.91 | | 926.20 | | 1.01 | | 8889.73 | | 0.39 | |  |
| QJP-39-R-1-71 | 0.08 | | 1.06 | | 0.05 | | 0.48 | | 2.87 | | | 0.00 | | | 21.03 | | | 7.19 | | | 69.73 | | | 23.94 | | 83.05 | | 15.89 | | 141.32 | | 23.14 | | 696.12 | | 0.45 | | 11478.58 | | 0.35 | |  |
| QJP-39-R-1-72 | 1.05 | | 16.58 | | 0.39 | | 3.30 | | 3.37 | | | 0.79 | | | 18.68 | | | 5.37 | | | 66.18 | | | 23.48 | | 106.10 | | 23.58 | | 230.34 | | 41.47 | | 753.24 | | 0.90 | | 9336.57 | | 0.73 | |  |
| QJP-39-R-1-73 | 0.04 | | 15.59 | | 0.29 | | 5.66 | | 14.30 | | | 3.50 | | | 85.37 | | | 29.13 | | | 341.27 | | | 122.91 | | 515.13 | | 108.19 | | 1024.22 | | 175.70 | | 3526.17 | | 2.08 | | 7801.01 | | 0.92 | |  |
| QJP-39-R-1-74 | 0.72 | | 2.96 | | 0.56 | | 4.24 | | 4.01 | | | 0.60 | | | 18.73 | | | 5.39 | | | 56.70 | | | 13.95 | | 46.08 | | 8.28 | | 73.66 | | 11.60 | | 444.28 | | 0.34 | | 11544.76 | | 0.51 | |  |
| QJP-39-R-1-75 | 0.00 | | 9.55 | | 0.07 | | 0.41 | | 0.49 | | | 0.55 | | | 2.88 | | | 0.94 | | | 14.79 | | | 5.14 | | 26.47 | | 6.90 | | 74.32 | | 15.58 | | 186.90 | | 0.64 | | 9353.16 | | 0.62 | |  |
| QJP-39-R-1-76 | 0.03 | | 5.22 | | 0.13 | | 1.72 | | 4.27 | | | 0.28 | | | 24.25 | | | 7.19 | | | 85.74 | | | 30.97 | | 131.23 | | 28.41 | | 258.04 | | 43.00 | | 914.38 | | 2.25 | | 8699.51 | | 0.96 | |  |
| QJP-39-R-1-77 | 0.22 | | 25.53 | | 0.60 | | 10.34 | | 12.80 | | | 0.36 | | | 59.55 | | | 15.64 | | | 171.81 | | | 61.30 | | 255.08 | | 52.20 | | 483.37 | | 83.75 | | 1684.10 | | 3.14 | | 8612.10 | | 0.90 | |  |
| QJP-39-R-1-78 | 9.32 | | 65.28 | | 8.06 | | 43.62 | | 25.96 | | | 9.35 | | | 45.37 | | | 15.56 | | | 132.38 | | | 39.42 | | 168.36 | | 37.42 | | 381.73 | | 72.16 | | 1192.17 | | 3.52 | | 13616.01 | | 4.77 | |  |
| QJP-39-R-1-79 | 3.69 | | 29.49 | | 1.20 | | 7.68 | | 6.29 | | | 0.60 | | | 26.01 | | | 6.65 | | | 84.36 | | | 31.22 | | 138.83 | | 30.17 | | 289.54 | | 51.50 | | 923.92 | | 1.85 | | 8358.90 | | 0.71 | |  |
| QJP-39-R-1-80 | 0.11 | | 12.01 | | 0.41 | | 3.59 | | 5.15 | | | 1.96 | | | 24.50 | | | 7.70 | | | 87.61 | | | 32.07 | | 135.78 | | 27.84 | | 272.65 | | 50.05 | | 913.95 | | 1.77 | | 8350.68 | | 0.38 | |  |
| QJP-39-R-1-81 | 115.69 | | 459.73 | | 80.83 | | 442.15 | | 260.30 | | | 75.88 | | | 463.54 | | | 132.40 | | | 1228.91 | | | 324.08 | | 1205.42 | | 250.23 | | 2336.74 | | 332.14 | | 8653.39 | | 15.55 | | 10635.12 | | 2.05 | |  |
| QJP-39-R-1-82 | 8.62 | | 118.34 | | 5.35 | | 32.29 | | 21.76 | | | 5.76 | | | 56.70 | | | 15.68 | | | 154.58 | | | 50.01 | | 208.06 | | 45.44 | | 416.97 | | 70.63 | | 1546.06 | | 3.60 | | 8682.26 | | 1.08 | |  |
| QJP-39-R-1-83 | 0.00 | | 20.64 | | 0.07 | | 1.34 | | 1.47 | | | 0.40 | | | 8.27 | | | 3.24 | | | 35.61 | | | 14.20 | | 68.55 | | 16.42 | | 179.78 | | 33.55 | | 475.52 | | 2.26 | | 11415.21 | | 1.44 | |  |
| QJP-39-R-1-84 | 0.06 | | 3.00 | | 0.06 | | 0.86 | | 1.47 | | | 0.01 | | | 8.62 | | | 2.39 | | | 28.70 | | | 10.86 | | 49.95 | | 10.83 | | 105.66 | | 19.56 | | 310.15 | | 0.90 | | 7843.54 | | 0.42 | |  |
| QJP-39-R-1-85 | 0.00 | | 15.00 | | 0.08 | | 0.55 | | 1.66 | | | 0.61 | | | 12.93 | | | 3.30 | | | 47.49 | | | 21.15 | | 106.46 | | 26.20 | | 293.40 | | 63.44 | | 683.28 | | 1.99 | | 10781.51 | | 0.74 | |  |
| QJP-39-R-1-86 | 0.23 | | 16.16 | | 0.23 | | 1.86 | | 1.76 | | | 0.47 | | | 8.82 | | | 2.75 | | | 29.06 | | | 11.79 | | 59.62 | | 14.00 | | 159.31 | | 31.47 | | 390.00 | | 0.80 | | 9948.19 | | 0.32 | |  |
| QJP-39-R-1-87 | 2.66 | | 18.56 | | 0.91 | | 5.79 | | 3.65 | | | 1.36 | | | 17.29 | | | 5.48 | | | 60.24 | | | 23.90 | | 107.15 | | 23.47 | | 241.51 | | 43.26 | | 713.01 | | 1.02 | | 8472.96 | | 0.47 | |  |
| QJP-39-R-1-88 | 0.19 | | 39.45 | | 0.76 | | 11.21 | | 11.32 | | | 3.48 | | | 37.25 | | | 8.09 | | | 73.65 | | | 21.08 | | 83.72 | | 15.65 | | 153.57 | | 27.20 | | 625.38 | | 1.84 | | 10850.82 | | 0.78 | |  |
| QJP-39-R-1-89 | 11.56 | | 36.03 | | 8.46 | | 45.12 | | 27.10 | | | 8.04 | | | 43.92 | | | 13.04 | | | 110.78 | | | 30.44 | | 118.63 | | 25.34 | | 250.73 | | 44.58 | | 847.45 | | 0.81 | | 7909.71 | | 0.36 | |  |
| QJP-39-R-1-90 | 7.35 | | 22.88 | | 4.91 | | 26.72 | | 13.33 | | | 4.69 | | | 28.30 | | | 7.58 | | | 69.86 | | | 22.81 | | 113.11 | | 25.47 | | 271.31 | | 50.67 | | 733.85 | | 1.47 | | 11090.25 | | 1.36 | |  |
| QJP-39-R-1-91 | 25.90 | | 143.76 | | 25.25 | | 154.16 | | 99.18 | | | 36.81 | | | 158.01 | | | 41.10 | | | 406.51 | | | 105.41 | | 412.43 | | 86.29 | | 783.00 | | 116.87 | | 3038.55 | | 1.89 | | 10816.07 | | 1.05 | |  |
| QJP-39-R-1-92 | 0.10 | | 2.85 | | 0.26 | | 5.48 | | 10.80 | | | 0.41 | | | 52.54 | | | 15.98 | | | 187.82 | | | 65.25 | | 286.44 | | 58.70 | | 531.82 | | 90.50 | | 1932.01 | | 0.80 | | 11041.00 | | 0.77 | |  |
| QJP-39-R-1-93 | 0.00 | | 0.01 | | 0.00 | | 0.09 | | 0.42 | | | 0.29 | | | 4.25 | | | 2.24 | | | 32.88 | | | 12.74 | | 55.27 | | 11.26 | | 100.22 | | 15.06 | | 369.08 | | 0.00 | | 0.01 | | 0.00 | |  |
| QJP-39-R-1-94 | 0.00 | | 16.16 | | 0.07 | | 1.71 | | 3.52 | | | 1.00 | | | 21.71 | | | 6.90 | | | 73.96 | | | 27.35 | | 123.99 | | 26.28 | | 244.51 | | 46.31 | | 839.22 | | 1.37 | | 8834.59 | | 0.53 | |  |
| QJP-39-R-1-95 | 0.08 | | 8.60 | | 0.04 | | 0.56 | | 0.87 | | | 0.65 | | | 6.61 | | | 1.52 | | | 23.54 | | | 10.22 | | 57.63 | | 14.67 | | 178.28 | | 40.63 | | 355.94 | | 0.74 | | 7387.77 | | 0.41 | |  |
| QJP-39-R-1-96 | 0.00 | | 27.46 | | 0.05 | | 1.34 | | 1.94 | | | 0.49 | | | 10.54 | | | 3.31 | | | 41.73 | | | 15.79 | | 78.77 | | 19.55 | | 219.96 | | 42.91 | | 557.82 | | 2.67 | | 10232.26 | | 2.14 | |  |
| QJP-39-R-1-97 | 0.11 | | 47.88 | | 0.29 | | 4.64 | | 7.31 | | | 2.53 | | | 35.30 | | | 12.26 | | | 139.56 | | | 56.18 | | 254.72 | | 57.95 | | 536.03 | | 103.17 | | 1711.66 | | 4.01 | | 7942.49 | | 0.88 | |  |
| QJP-39-R-1-98 | 16.76 | | 105.75 | | 12.53 | | 69.19 | | 41.14 | | | 12.78 | | | 79.37 | | | 22.63 | | | 224.29 | | | 66.60 | | 285.04 | | 62.92 | | 597.43 | | 99.99 | | 2036.38 | | 2.45 | | 8926.20 | | 0.90 | |  |
| QJP-39-R-1-99 | 0.12 | | 16.41 | | 0.11 | | 0.80 | | 1.38 | | | 0.23 | | | 3.83 | | | 1.78 | | | 23.12 | | | 8.43 | | 41.17 | | 11.42 | | 113.75 | | 24.16 | | 291.81 | | 1.48 | | 11087.86 | | 1.50 | |  |
| QJP-39-R-1-100 | 64.11 | | 261.40 | | 46.17 | | 261.16 | | 151.90 | | | 49.30 | | | 262.46 | | | 73.22 | | | 740.94 | | | 203.00 | | 774.81 | | 168.74 | | 1462.59 | | 219.71 | | 5748.75 | | 5.00 | | 12098.03 | | 2.66 | |  |
| QJP-39-R-1-101 | 0.05 | | 2.50 | | 0.02 | | 0.21 | | 0.39 | | | 0.23 | | | 5.36 | | | 1.63 | | | 21.72 | | | 8.78 | | 44.47 | | 10.82 | | 119.45 | | 25.85 | | 294.51 | | 0.77 | | 10528.84 | | 0.91 | |  |
| QJP-39-R-1-102 | 24.01 | | 311.37 | | 61.51 | | 421.41 | | 238.89 | | | 73.55 | | | 431.55 | | | 94.19 | | | 723.16 | | | 170.90 | | 561.92 | | 101.54 | | 780.82 | | 119.96 | | 4895.18 | | 4.85 | | 11945.94 | | 5.16 | |  |
| QJP-39-R-1-103 | 0.03 | | 5.03 | | 0.06 | | 1.09 | | 2.23 | | | 0.62 | | | 15.98 | | | 7.39 | | | 98.63 | | | 40.68 | | 189.69 | | 44.50 | | 413.72 | | 80.10 | | 1214.32 | | 1.61 | | 7679.48 | | 0.84 | |  |
| QJP-39-R-1-104 | 0.12 | | 35.94 | | 0.29 | | 4.68 | | 6.22 | | | 1.28 | | | 33.77 | | | 10.18 | | | 108.36 | | | 40.57 | | 178.71 | | 39.23 | | 363.56 | | 66.86 | | 1243.66 | | 2.96 | | 8335.17 | | 0.95 | |  |
| QJP-39-R-1-105 | 0.73 | | 12.44 | | 0.37 | | 4.52 | | 5.81 | | | 0.20 | | | 29.53 | | | 9.60 | | | 112.93 | | | 40.93 | | 188.70 | | 40.07 | | 348.75 | | 60.39 | | 1288.38 | | 2.79 | | 9864.55 | | 0.77 | |  |
| QJP-39-R-1-106 | 76.35 | | 396.51 | | 53.54 | | 301.56 | | 166.75 | | | 48.87 | | | 307.75 | | | 83.27 | | | 776.71 | | | 201.65 | | 784.31 | | 157.84 | | 1439.56 | | 246.48 | | 6079.27 | | 8.88 | | 8668.71 | | 2.12 | |  |
| QJP-39-R-1-107 | 0.00 | | 5.51 | | 0.04 | | 1.62 | | 3.39 | | | 0.71 | | | 22.35 | | | 7.66 | | | 97.12 | | | 34.96 | | 159.58 | | 35.05 | | 324.10 | | 59.02 | | 1089.83 | | 1.07 | | 8440.42 | | 0.61 | |  |
| QJP-39-R-1-108 | 1.19 | | 28.61 | | 0.79 | | 5.43 | | 5.12 | | | 0.74 | | | 20.57 | | | 4.95 | | | 63.99 | | | 22.83 | | 103.38 | | 24.00 | | 235.87 | | 45.66 | | 719.57 | | 2.54 | | 9935.84 | | 0.96 | |  |
| QJP-39-R-1-109 | 1.05 | | 39.27 | | 3.27 | | 22.35 | | 15.71 | | | 4.47 | | | 31.99 | | | 9.16 | | | 91.99 | | | 28.72 | | 127.78 | | 27.85 | | 258.12 | | 46.18 | | 941.17 | | 2.51 | | 9373.42 | | 0.93 | |  |
| QJP-39-R-1-110 | 54.66 | | 153.96 | | 13.52 | | 57.29 | | 11.07 | | | 2.62 | | | 23.07 | | | 6.46 | | | 72.93 | | | 28.21 | | 126.99 | | 31.36 | | 320.91 | | 61.44 | | 883.27 | | 2.21 | | 9810.39 | | 0.66 | |  |
| QJP-39-R-1-111 | 0.00 | | 19.32 | | 0.02 | | 0.92 | | 1.81 | | | 0.44 | | | 7.67 | | | 2.74 | | | 32.78 | | | 13.34 | | 65.00 | | 16.85 | | 173.43 | | 33.85 | | 463.89 | | 2.10 | | 9662.94 | | 1.63 | |  |
| QJP-39-R-1-112 | 0.03 | | 14.58 | | 0.09 | | 1.16 | | 1.41 | | | 0.09 | | | 9.64 | | | 3.26 | | | 40.04 | | | 16.42 | | 77.21 | | 18.83 | | 182.24 | | 32.01 | | 508.93 | | 4.86 | | 10213.29 | | 3.71 | |  |
| QJP-39-R-1-113 | 0.03 | | 2.31 | | 0.27 | | 3.36 | | 3.07 | | | 0.53 | | | 19.62 | | | 7.65 | | | 97.33 | | | 38.17 | | 183.01 | | 37.86 | | 352.65 | | 60.70 | | 1185.87 | | 1.00 | | 11651.26 | | 0.66 | |  |
| QJP-39-R-1-114 | 0.11 | | 10.79 | | 0.85 | | 9.09 | | 13.20 | | | 3.76 | | | 64.90 | | | 20.59 | | | 233.85 | | | 84.77 | | 351.50 | | 69.61 | | 598.66 | | 102.00 | | 2470.73 | | 3.58 | | 9017.98 | | 1.38 | |  |
| QJP-39-R-1-115 | 0.24 | | 14.60 | | 1.00 | | 10.94 | | 12.29 | | | 1.86 | | | 39.22 | | | 11.54 | | | 114.91 | | | 36.83 | | 142.51 | | 29.85 | | 267.49 | | 43.26 | | 1095.15 | | 2.81 | | 9219.41 | | 1.21 | |  |
| QJP-39-R-1-116 | 0.06 | | 26.03 | | 0.03 | | 1.07 | | 2.38 | | | 0.60 | | | 12.84 | | | 3.86 | | | 38.08 | | | 14.47 | | 66.41 | | 15.35 | | 162.01 | | 31.74 | | 462.37 | | 1.22 | | 10312.17 | | 0.46 | |  |
| QJP-39-R-1-117 | 1.85 | | 31.19 | | 0.78 | | 6.00 | | 6.24 | | | 1.74 | | | 28.54 | | | 8.99 | | | 124.42 | | | 49.88 | | 242.63 | | 54.61 | | 539.95 | | 102.30 | | 1512.20 | | 3.36 | | 8626.28 | | 0.93 | |  |
| QJP-39-R-1-118 | 0.69 | | 36.14 | | 0.85 | | 5.94 | | 3.68 | | | 1.53 | | | 12.38 | | | 3.53 | | | 41.01 | | | 16.82 | | 81.28 | | 21.74 | | 248.98 | | 54.60 | | 570.20 | | 2.01 | | 10677.03 | | 0.62 | |  |
| QJP-39-R-1-119 | 0.04 | | 10.09 | | 0.21 | | 1.90 | | 2.32 | | | 0.83 | | | 16.97 | | | 4.96 | | | 55.36 | | | 21.46 | | 91.92 | | 20.36 | | 195.52 | | 37.83 | | 652.67 | | 0.62 | | 9591.78 | | 0.34 | |  |
| QJP-39-R-1-120 | 22.21 | | 127.92 | | 18.89 | | 110.74 | | 67.30 | | | 21.53 | | | 128.12 | | | 36.29 | | | 333.26 | | | 97.96 | | 377.54 | | 81.14 | | 726.37 | | 118.20 | | 2726.51 | | 2.16 | | 8533.76 | | 1.00 | |  |
| QJP-39-R-1-121 | 9.63 | | 30.42 | | 2.31 | | 9.91 | | 3.77 | | | 0.99 | | | 13.81 | | | 4.37 | | | 52.76 | | | 22.49 | | 120.00 | | 28.11 | | 293.34 | | 62.06 | | 721.33 | | 1.37 | | 9697.72 | | 0.59 | |  |
| QJP-39-R-1-122 | 5.91 | | 87.54 | | 2.06 | | 14.81 | | 12.83 | | | 2.41 | | | 50.10 | | | 14.72 | | | 171.89 | | | 61.56 | | 274.59 | | 56.35 | | 511.09 | | 93.76 | | 1797.05 | | 1.66 | | 7743.82 | | 0.54 | |  |
| QJP-39-R-1-123 | 3.67 | | 47.19 | | 1.80 | | 13.54 | | 11.02 | | | 2.71 | | | 36.15 | | | 10.27 | | | 115.03 | | | 39.33 | | 175.25 | | 37.58 | | 361.33 | | 66.92 | | 1229.05 | | 1.14 | | 9572.83 | | 0.57 | |  |
| QJP-39-R-1-124 | 0.03 | | 13.65 | | 0.05 | | 0.60 | | 1.87 | | | 0.50 | | | 9.88 | | | 3.15 | | | 31.76 | | | 11.89 | | 56.21 | | 11.92 | | 115.77 | | 23.72 | | 362.03 | | 0.51 | | 9849.51 | | 0.20 | |  |
| QJP-39-R-1-125 | 0.00 | | 18.81 | | 0.03 | | 0.60 | | 1.88 | | | 0.60 | | | 10.43 | | | 3.07 | | | 33.62 | | | 13.18 | | 64.55 | | 16.08 | | 175.49 | | 35.72 | | 436.77 | | 1.40 | | 11611.97 | | 1.22 | |  |
| QJP-39-R-1-126 | 0.10 | | 3.52 | | 0.00 | | 0.35 | | 0.57 | | | 0.52 | | | 1.85 | | | 0.75 | | | 10.12 | | | 4.94 | | 28.10 | | 7.87 | | 91.31 | | 21.64 | | 170.89 | | 0.49 | | 9003.41 | | 0.34 | |  |
| QJP-39-R-1-127 | 1.12 | | 70.86 | | 1.70 | | 11.43 | | 7.54 | | | 2.66 | | | 23.35 | | | 6.92 | | | 81.47 | | | 26.62 | | 115.29 | | 25.63 | | 249.25 | | 40.15 | | 809.32 | | 3.62 | | 10341.07 | | 2.26 | |  |
| QJP-39-R-1-128 | 28.88 | | 116.97 | | 17.31 | | 94.83 | | 58.09 | | | 14.32 | | | 120.14 | | | 35.56 | | | 366.17 | | | 113.21 | | 470.16 | | 99.35 | | 941.04 | | 156.13 | | 3218.46 | | 7.03 | | 9181.65 | | 2.72 | |  |
| QJP-39-R-1-129 | 28.65 | | 91.32 | | 6.92 | | 42.52 | | 18.89 | | | 5.32 | | | 38.39 | | | 10.13 | | | 106.20 | | | 33.18 | | 144.53 | | 31.05 | | 317.17 | | 61.30 | | 1053.58 | | 1.54 | | 10687.06 | | 0.62 | |  |
| QJP-39-R-1-130 | 0.00 | | 16.08 | | 0.00 | | 0.60 | | 1.07 | | | 0.49 | | | 5.70 | | | 1.85 | | | 23.91 | | | 9.74 | | 50.62 | | 12.57 | | 145.05 | | 29.97 | | 332.36 | | 1.29 | | 11079.83 | | 1.85 | |  |
| QJP-39-R-1-131 | 0.14 | | 35.97 | | 0.96 | | 11.89 | | 15.30 | | | 3.29 | | | 55.34 | | | 15.39 | | | 160.83 | | | 53.99 | | 227.27 | | 47.68 | | 428.38 | | 73.07 | | 1553.53 | | 2.26 | | 8075.31 | | 0.78 | |  |
| QJP-39-R-1-132 | 0.00 | | 19.24 | | 0.03 | | 0.57 | | 1.59 | | | 0.52 | | | 10.62 | | | 3.60 | | | 37.85 | | | 13.95 | | 61.04 | | 13.65 | | 130.67 | | 25.90 | | 437.36 | | 0.92 | | 11619.69 | | 0.42 | |  |
| QJP-39-R-1-133 | 0.13 | | 35.93 | | 0.30 | | 4.93 | | 9.55 | | | 2.63 | | | 52.21 | | | 16.44 | | | 189.21 | | | 69.79 | | 312.48 | | 67.56 | | 621.69 | | 116.49 | | 2100.14 | | 1.76 | | 10296.32 | | 0.70 | |  |
| QJP-39-R-1-134 | 0.32 | | 14.92 | | 0.31 | | 2.86 | | 4.21 | | | 1.02 | | | 21.72 | | | 6.19 | | | 70.56 | | | 25.88 | | 116.08 | | 25.30 | | 232.87 | | 42.55 | | 792.87 | | 0.98 | | 10317.11 | | 0.60 | |  |
| QJP-39-R-1-135 | 0.57 | | 39.51 | | 0.91 | | 11.25 | | 13.35 | | | 4.95 | | | 60.99 | | | 18.01 | | | 188.79 | | | 71.17 | | 322.14 | | 73.76 | | 743.98 | | 138.23 | | 2345.33 | | 3.47 | | 8615.68 | | 1.24 | |  |
| QJP-39-R-1-136 | 0.07 | | 40.00 | | 0.05 | | 1.28 | | 3.82 | | | 0.75 | | | 15.65 | | | 4.90 | | | 55.01 | | | 20.32 | | 92.14 | | 22.11 | | 215.27 | | 40.30 | | 671.71 | | 3.20 | | 11563.86 | | 2.83 | |  |
| QJP-39-R-1-137 | 0.14 | | 21.89 | | 0.08 | | 0.80 | | 1.57 | | | 0.67 | | | 9.81 | | | 3.55 | | | 42.96 | | | 19.29 | | 100.08 | | 25.12 | | 278.01 | | 64.82 | | 655.04 | | 1.72 | | 13158.58 | | 1.03 | |  |
| QJP-39-R-1-138 | 42.89 | | 116.26 | | 14.63 | | 74.20 | | 23.39 | | | 3.06 | | | 50.73 | | | 13.73 | | | 151.12 | | | 53.40 | | 229.35 | | 49.31 | | 451.71 | | 80.01 | | 1507.30 | | 3.72 | | 10758.03 | | 1.79 | |  |
| QJP-39-R-1-139 | 1.22 | | 71.05 | | 0.70 | | 6.13 | | 8.36 | | | 2.37 | | | 48.56 | | | 13.89 | | | 169.51 | | | 62.27 | | 267.89 | | 56.80 | | 530.47 | | 97.43 | | 1762.43 | | 1.32 | | 10099.56 | | 0.53 | |  |
| QJP-39-R-1-140 | 22.94 | | 103.13 | | 16.15 | | 89.12 | | 51.27 | | | 17.51 | | | 87.34 | | | 22.77 | | | 219.56 | | | 58.37 | | 228.76 | | 52.30 | | 529.44 | | 101.95 | | 1721.89 | | 2.76 | | 12117.07 | | 0.83 | |  |
| SBY-2-R-1 | |  | |  | |  | |  | |  | | |  | | |  | | |  | | |  | | |  | |  | |  | |  | |  | |  | |  | |  | |  | |
| SBY-2-R-1-01 | 2.78 | | 61.27 | | 0.88 | | 5.80 | | 5.19 | | | 1.52 | | | 22.17 | | | 7.31 | | | 92.58 | | | 34.88 | | 167.02 | | 38.10 | | 378.32 | | 69.54 | | 1128.88 | | 3.26 | | 9665.89 | | 1.17 | |  |
| SBY-2-R-1-02 | 6.44 | | 63.99 | | 7.97 | | 49.39 | | 37.25 | | | 11.96 | | | 68.83 | | | 19.88 | | | 188.67 | | | 51.52 | | 218.18 | | 48.52 | | 474.77 | | 79.46 | | 1568.91 | | 4.73 | | 11034.88 | | 3.06 | |  |
| SBY-2-R-1-03 | 0.32 | | 31.83 | | 0.29 | | 3.84 | | 5.64 | | | 2.14 | | | 22.11 | | | 6.60 | | | 75.93 | | | 26.58 | | 123.29 | | 27.37 | | 272.00 | | 51.84 | | 872.27 | | 1.64 | | 8108.78 | | 0.69 | |  |
| SBY-2-R-1-04 | 62.70 | | 146.89 | | 17.54 | | 80.20 | | 16.46 | | | 1.44 | | | 23.37 | | | 5.47 | | | 54.23 | | | 18.71 | | 81.24 | | 17.35 | | 166.24 | | 28.98 | | 583.18 | | 2.32 | | 7725.47 | | 0.72 | |  |
| SBY-2-R-1-05 | 0.02 | | 21.00 | | 0.20 | | 3.01 | | 6.32 | | | 1.06 | | | 39.35 | | | 14.34 | | | 175.55 | | | 64.51 | | 289.33 | | 59.48 | | 562.91 | | 98.77 | | 1997.41 | | 2.72 | | 10785.71 | | 1.34 | |  |
| SBY-2-R-1-06 | 1.86 | | 18.93 | | 0.45 | | 2.97 | | 2.75 | | | 0.75 | | | 12.43 | | | 4.18 | | | 55.69 | | | 21.85 | | 109.94 | | 26.68 | | 283.74 | | 58.11 | | 729.02 | | 1.96 | | 9427.42 | | 0.93 | |  |
| SBY-2-R-1-07 | 0.01 | | 2.90 | | 0.13 | | 2.62 | | 7.56 | | | 0.10 | | | 42.57 | | | 15.18 | | | 182.57 | | | 65.43 | | 285.52 | | 58.07 | | 512.94 | | 83.62 | | 1916.41 | | 1.33 | | 11003.16 | | 0.95 | |  |
| SBY-2-R-1-08 | 0.02 | | 3.48 | | 0.04 | | 0.42 | | 1.55 | | | 0.15 | | | 12.88 | | | 6.72 | | | 101.58 | | | 39.86 | | 198.85 | | 45.98 | | 455.42 | | 82.87 | | 1269.46 | | 3.52 | | 12079.27 | | 2.67 | |  |
| SBY-2-R-1-09 | 0.03 | | 23.92 | | 0.07 | | 1.05 | | 2.08 | | | 0.65 | | | 11.09 | | | 3.91 | | | 49.54 | | | 19.47 | | 92.57 | | 20.84 | | 214.72 | | 40.87 | | 608.55 | | 1.90 | | 10176.83 | | 1.02 | |  |
| SBY-2-R-1-10 | 0.04 | | 2.72 | | 0.08 | | 1.34 | | 4.09 | | | 0.15 | | | 29.97 | | | 11.99 | | | 160.00 | | | 61.01 | | 276.93 | | 58.36 | | 551.62 | | 98.11 | | 1760.38 | | 1.37 | | 12163.24 | | 0.95 | |  |
| SBY-2-R-1-11 | 0.01 | | 2.39 | | 0.03 | | 0.83 | | 2.72 | | | 0.11 | | | 18.94 | | | 7.78 | | | 105.08 | | | 41.53 | | 196.14 | | 43.00 | | 410.55 | | 73.80 | | 1262.24 | | 1.77 | | 10936.37 | | 1.15 | |  |
| SBY-2-R-1-12 | 19.92 | | 222.07 | | 17.45 | | 103.98 | | 82.69 | | | 26.91 | | | 154.79 | | | 43.68 | | | 404.12 | | | 107.46 | | 444.07 | | 95.59 | | 919.85 | | 156.12 | | 2788.86 | | 4.89 | | 10698.17 | | 3.40 | |  |
| SBY-2-R-1-13 | 0.20 | | 5.00 | | 0.33 | | 4.30 | | 8.75 | | | 0.77 | | | 49.21 | | | 16.76 | | | 211.34 | | | 76.00 | | 345.06 | | 71.11 | | 671.55 | | 117.14 | | 2337.34 | | 1.69 | | 11283.74 | | 0.90 | |  |
| SBY-2-R-1-14 | 0.56 | | 42.86 | | 0.64 | | 6.94 | | 8.94 | | | 2.01 | | | 36.45 | | | 11.04 | | | 127.23 | | | 44.16 | | 198.97 | | 42.70 | | 417.39 | | 73.47 | | 1368.22 | | 1.92 | | 9793.26 | | 0.98 | |  |
| SBY-2-R-1-15 | 16.67 | | 256.31 | | 20.24 | | 121.94 | | 96.40 | | | 32.29 | | | 160.77 | | | 44.80 | | | 389.04 | | | 97.45 | | 383.90 | | 81.98 | | 809.72 | | 138.11 | | 2625.59 | | 5.80 | | 11372.97 | | 3.86 | |  |
| SBY-2-R-1-16 | 0.00 | | 16.25 | | 0.05 | | 1.02 | | 2.48 | | | 0.24 | | | 14.33 | | | 5.03 | | | 66.84 | | | 24.66 | | 118.96 | | 26.38 | | 254.66 | | 46.54 | | 786.76 | | 2.22 | | 10443.87 | | 1.26 | |  |
| SBY-2-R-1-17 | 0.02 | | 3.86 | | 0.11 | | 1.76 | | 5.62 | | | 0.29 | | | 35.98 | | | 13.59 | | | 178.58 | | | 65.71 | | 297.01 | | 62.79 | | 584.49 | | 100.33 | | 1971.83 | | 0.48 | | 11134.37 | | 0.24 | |  |
| SBY-2-R-1-18 | 0.03 | | 2.20 | | 0.01 | | 0.10 | | 0.34 | | | 0.11 | | | 4.53 | | | 2.86 | | | 54.35 | | | 26.96 | | 161.22 | | 41.07 | | 466.98 | | 95.02 | | 983.09 | | 31.86 | | 12544.59 | | 26.38 | |  |
| SBY-2-R-1-19 | 0.00 | | 12.97 | | 0.06 | | 1.28 | | 2.97 | | | 0.19 | | | 17.26 | | | 5.77 | | | 76.08 | | | 26.95 | | 122.87 | | 25.07 | | 228.39 | | 39.47 | | 824.84 | | 1.93 | | 11156.97 | | 1.03 | |  |
| SBY-2-R-1-20 | 0.01 | | 8.71 | | 0.15 | | 1.88 | | 4.39 | | | 1.08 | | | 23.22 | | | 7.76 | | | 93.71 | | | 32.94 | | 152.39 | | 31.38 | | 294.59 | | 54.10 | | 1027.62 | | 1.12 | | 8917.69 | | 0.65 | |  |
| SBY-2-R-1-21 | 3.40 | | 23.26 | | 1.02 | | 5.97 | | 4.91 | | | 0.30 | | | 22.85 | | | 7.99 | | | 100.08 | | | 35.36 | | 160.89 | | 33.48 | | 298.75 | | 49.04 | | 1068.62 | | 11.65 | | 10974.47 | | 5.10 | |  |
| SBY-2-R-1-22 | 0.02 | | 45.67 | | 0.11 | | 1.90 | | 3.20 | | | 0.77 | | | 14.41 | | | 4.67 | | | 51.53 | | | 18.27 | | 82.53 | | 18.02 | | 174.75 | | 31.11 | | 575.60 | | 3.24 | | 9541.40 | | 1.27 | |  |
| SBY-2-R-1-23 | 0.05 | | 16.83 | | 0.04 | | 0.86 | | 2.32 | | | 0.24 | | | 13.97 | | | 5.29 | | | 72.64 | | | 26.66 | | 130.35 | | 29.28 | | 290.43 | | 50.06 | | 862.53 | | 3.27 | | 9919.31 | | 1.47 | |  |
| SBY-2-R-1-24 | 0.00 | | 9.33 | | 0.03 | | 0.52 | | 1.19 | | | 0.23 | | | 7.27 | | | 2.89 | | | 36.61 | | | 14.96 | | 76.04 | | 18.25 | | 196.67 | | 38.09 | | 486.06 | | 0.53 | | 10699.41 | | 11.00 | |  |
| SBY-2-R-1-25 | 0.00 | | 7.06 | | 0.05 | | 0.86 | | 2.72 | | | 0.10 | | | 17.73 | | | 7.52 | | | 104.14 | | | 39.26 | | 190.21 | | 41.82 | | 401.36 | | 65.42 | | 1238.29 | | 1.90 | | 11459.43 | | 1.59 | |  |
| SBY-2-R-1-26 | 0.07 | | 8.92 | | 0.13 | | 2.22 | | 4.48 | | | 0.55 | | | 24.64 | | | 8.39 | | | 107.01 | | | 39.01 | | 178.41 | | 36.89 | | 349.02 | | 59.41 | | 1193.03 | | 6.13 | | 9290.42 | | 1.92 | |  |
| SBY-2-R-1-27 | 0.14 | | 18.84 | | 0.18 | | 2.04 | | 2.62 | | | 0.44 | | | 11.10 | | | 3.80 | | | 50.70 | | | 19.88 | | 96.26 | | 23.10 | | 241.94 | | 41.28 | | 659.85 | | 5.26 | | 10448.72 | | 2.10 | |  |
| SBY-2-R-1-28 | 0.01 | | 17.51 | | 0.39 | | 6.34 | | 11.04 | | | 0.28 | | | 52.47 | | | 17.01 | | | 195.71 | | | 65.96 | | 282.37 | | 55.67 | | 498.05 | | 76.52 | | 1936.29 | | 8.06 | | 10621.79 | | 3.77 | |  |
| SBY-2-R-1-29 | 0.81 | | 15.73 | | 0.85 | | 8.25 | | 12.14 | | | 1.48 | | | 50.44 | | | 16.70 | | | 184.61 | | | 61.31 | | 262.38 | | 51.28 | | 459.34 | | 72.49 | | 1827.15 | | 3.16 | | 11046.24 | | 1.57 | |  |
| SBY-2-R-1-30 | 0.07 | | 15.13 | | 0.18 | | 2.84 | | 4.46 | | | 1.14 | | | 13.93 | | | 4.16 | | | 41.72 | | | 14.50 | | 63.23 | | 13.65 | | 146.21 | | 26.90 | | 455.34 | | 3.20 | | 10456.01 | | 0.83 | |  |
| SBY-2-R-1-31 | 0.01 | | 6.80 | | 0.03 | | 1.05 | | 2.95 | | | 0.52 | | | 17.93 | | | 6.36 | | | 76.56 | | | 28.34 | | 128.76 | | 26.46 | | 253.70 | | 43.47 | | 850.60 | | 2.18 | | 9910.91 | | 1.22 | |  |
| SBY-2-R-1-32 | 0.02 | | 0.55 | | 0.05 | | 1.01 | | 3.56 | | | 0.08 | | | 26.43 | | | 9.08 | | | 91.48 | | | 25.72 | | 96.27 | | 17.85 | | 160.15 | | 24.85 | | 825.80 | | 0.39 | | 10969.83 | | 0.26 | |  |
| SBY-2-R-1-33 | 0.03 | | 13.95 | | 0.01 | | 0.75 | | 2.51 | | | 0.30 | | | 15.74 | | | 5.79 | | | 75.27 | | | 29.95 | | 146.27 | | 33.26 | | 347.11 | | 58.49 | | 918.18 | | 2.60 | | 11010.69 | | 1.26 | |  |
| SBY-2-R-1-34 | 0.03 | | 20.33 | | 0.12 | | 2.43 | | 5.66 | | | 0.57 | | | 30.88 | | | 10.19 | | | 108.49 | | | 38.17 | | 165.99 | | 34.96 | | 334.15 | | 54.43 | | 1136.86 | | 2.82 | | 13368.34 | | 2.11 | |  |
| SBY-2-R-1-35 | 0.21 | | 12.31 | | 0.29 | | 3.86 | | 6.23 | | | 0.81 | | | 22.48 | | | 6.13 | | | 55.02 | | | 17.05 | | 66.53 | | 12.72 | | 118.22 | | 18.67 | | 538.29 | | 2.70 | | 9149.78 | | 0.67 | |  |
| SBY-2-R-1-36 | 0.00 | | 1.80 | | 0.07 | | 1.45 | | 5.05 | | | 0.28 | | | 19.59 | | | 3.53 | | | 21.65 | | | 3.91 | | 9.47 | | 1.32 | | 10.43 | | 1.56 | | 118.59 | | 0.84 | | 13167.57 | | 0.59 | |  |
| SBY-2-R-1-37 | 0.21 | | 49.88 | | 0.28 | | 2.51 | | 3.59 | | | 1.36 | | | 16.18 | | | 5.62 | | | 66.35 | | | 24.93 | | 118.58 | | 28.44 | | 303.20 | | 57.23 | | 825.18 | | 6.68 | | 11316.02 | | 5.03 | |  |
| SBY-2-R-1-38 | 0.11 | | 49.26 | | 0.64 | | 9.67 | | 14.62 | | | 3.31 | | | 59.56 | | | 18.45 | | | 201.23 | | | 71.15 | | 307.32 | | 64.98 | | 622.10 | | 99.57 | | 2124.24 | | 4.79 | | 9366.76 | | 2.02 | |  |
| SBY-2-R-1-39 | 4.82 | | 53.19 | | 1.55 | | 10.76 | | 8.08 | | | 1.53 | | | 22.33 | | | 6.24 | | | 64.49 | | | 24.08 | | 133.95 | | 46.40 | | 703.52 | | 140.63 | | 801.14 | | 3.23 | | 11635.92 | | 1.70 | |  |
| SBY-2-R-1-40 | 0.02 | | 1.15 | | 0.11 | | 2.36 | | 7.43 | | | 0.10 | | | 43.13 | | | 14.78 | | | 150.23 | | | 46.51 | | 186.11 | | 35.80 | | 319.88 | | 49.78 | | 1407.26 | | 0.67 | | 12964.90 | | 0.44 | |  |
| SBY-2-R-1-41 | 0.01 | | 21.54 | | 0.42 | | 6.53 | | 9.94 | | | 2.09 | | | 42.95 | | | 12.59 | | | 142.16 | | | 50.19 | | 217.41 | | 43.96 | | 407.48 | | 65.81 | | 1529.08 | | 1.13 | | 10008.98 | | 0.61 | |  |
| SBY-2-R-1-42 | 3.22 | | 14.46 | | 1.22 | | 9.49 | | 10.36 | | | 0.94 | | | 50.18 | | | 14.94 | | | 163.34 | | | 55.84 | | 225.30 | | 44.02 | | 389.57 | | 61.33 | | 1579.87 | | 3.07 | | 9474.98 | | 1.16 | |  |
| SBY-2-R-1-43 | 0.01 | | 16.03 | | 0.13 | | 2.46 | | 4.41 | | | 1.50 | | | 23.41 | | | 7.80 | | | 94.85 | | | 36.43 | | 163.52 | | 35.45 | | 360.01 | | 59.84 | | 1087.85 | | 3.41 | | 10207.07 | | 1.97 | |  |
| SBY-2-R-1-44 | 0.00 | | 40.72 | | 0.22 | | 3.41 | | 6.27 | | | 1.52 | | | 28.47 | | | 8.38 | | | 93.87 | | | 34.31 | | 155.25 | | 33.53 | | 336.54 | | 57.84 | | 1066.55 | | 2.19 | | 10001.66 | | 1.04 | |  |
| SBY-2-R-1-45 | 0.37 | | 49.51 | | 0.55 | | 5.21 | | 7.90 | | | 2.45 | | | 34.17 | | | 10.80 | | | 128.95 | | | 48.84 | | 224.37 | | 47.97 | | 487.82 | | 85.47 | | 1482.11 | | 3.71 | | 10366.44 | | 1.60 | |  |
| SBY-2-R-1-46 | 0.41 | | 5.57 | | 0.20 | | 4.21 | | 3.07 | | | 0.21 | | | 18.36 | | | 8.56 | | | 120.33 | | | 48.58 | | 234.08 | | 52.20 | | 510.55 | | 81.98 | | 1482.40 | | 3.14 | | 13147.79 | | 2.97 | |  |
| SBY-2-R-1-47 | 0.00 | | 58.53 | | 0.09 | | 2.04 | | 4.76 | | | 1.69 | | | 25.51 | | | 9.04 | | | 111.00 | | | 43.30 | | 205.48 | | 48.25 | | 493.78 | | 88.86 | | 1390.50 | | 3.87 | | 11167.26 | | 1.42 | |  |
| SBY-2-R-1-48 | 6.89 | | 47.79 | | 3.74 | | 23.05 | | 17.34 | | | 1.09 | | | 61.62 | | | 18.89 | | | 208.55 | | | 70.39 | | 296.01 | | 59.52 | | 559.42 | | 87.57 | | 2026.02 | | 5.58 | | 11174.31 | | 2.03 | |  |
| SBY-2-R-1-49 | 0.07 | | 26.65 | | 0.06 | | 1.39 | | 3.03 | | | 0.84 | | | 17.37 | | | 6.55 | | | 84.40 | | | 34.03 | | 173.19 | | 40.84 | | 452.85 | | 82.34 | | 1108.42 | | 4.49 | | 11086.02 | | 2.50 | |  |
| SBY-2-R-1-50 | 0.06 | | 0.26 | | 0.01 | | 0.31 | | 1.21 | | | 0.14 | | | 12.11 | | | 6.74 | | | 98.10 | | | 33.59 | | 135.46 | | 26.82 | | 249.24 | | 37.75 | | 1031.29 | | 2.34 | | 13712.58 | | 3.23 | |  |
| SBY-2-R-1-51 | 0.04 | | 6.45 | | 0.62 | | 9.00 | | 15.77 | | | 0.88 | | | 74.06 | | | 22.49 | | | 244.00 | | | 79.50 | | 310.64 | | 59.01 | | 511.99 | | 75.90 | | 2259.70 | | 1.79 | | 11458.41 | | 0.90 | |  |
| SBY-2-R-1-52 | 0.01 | | 4.19 | | 0.09 | | 1.99 | | 4.69 | | | 0.11 | | | 26.38 | | | 9.86 | | | 119.80 | | | 44.21 | | 200.66 | | 42.54 | | 406.55 | | 65.58 | | 1310.49 | | 1.93 | | 11392.54 | | 1.00 | |  |
| SBY-2-R-1-53 | 0.06 | | 38.46 | | 0.18 | | 3.20 | | 4.68 | | | 1.74 | | | 22.42 | | | 7.38 | | | 87.97 | | | 34.11 | | 161.91 | | 36.96 | | 372.93 | | 65.68 | | 1081.30 | | 2.70 | | 9209.08 | | 1.10 | |  |
| SBY-2-R-1-54 | 0.64 | | 11.31 | | 0.75 | | 6.38 | | 11.38 | | | 2.28 | | | 60.60 | | | 24.23 | | | 266.02 | | | 76.33 | | 276.39 | | 49.98 | | 406.88 | | 58.53 | | 2342.45 | | 3.01 | | 11831.51 | | 1.44 | |  |
| SBY-2-R-1-55 | 19.52 | | 92.30 | | 14.03 | | 78.94 | | 46.45 | | | 14.86 | | | 84.24 | | | 20.67 | | | 188.34 | | | 55.37 | | 233.46 | | 51.28 | | 530.97 | | 93.01 | | 1856.84 | | 2.20 | | 8472.98 | | 0.68 | |  |
| SBY-2-R-1-56 | 0.03 | | 10.28 | | 0.12 | | 2.35 | | 6.48 | | | 0.08 | | | 40.77 | | | 15.28 | | | 181.16 | | | 64.98 | | 271.78 | | 55.03 | | 498.04 | | 79.53 | | 2078.84 | | 4.03 | | 14097.78 | | 3.23 | |  |
| SBY-2-R-1-57 | 0.01 | | 5.61 | | 0.07 | | 0.93 | | 2.60 | | | 0.21 | | | 14.10 | | | 4.84 | | | 59.40 | | | 22.62 | | 101.62 | | 22.47 | | 215.81 | | 36.19 | | 677.63 | | 0.88 | | 10870.56 | | 0.56 | |  |
| SBY-2-R-1-58 | 0.08 | | 25.56 | | 0.12 | | 1.92 | | 4.19 | | | 1.11 | | | 24.95 | | | 9.18 | | | 117.82 | | | 47.57 | | 226.57 | | 52.56 | | 556.25 | | 97.75 | | 1453.26 | | 4.28 | | 10948.29 | | 2.07 | |  |
| SBY-2-R-1-59 | 0.00 | | 8.22 | | 0.06 | | 0.92 | | 2.67 | | | 0.19 | | | 17.98 | | | 7.46 | | | 104.00 | | | 42.40 | | 212.02 | | 49.89 | | 507.99 | | 88.22 | | 1284.76 | | 5.73 | | 11839.93 | | 4.86 | |  |
| SBY-2-R-1-60 | 2.14 | | 15.31 | | 0.83 | | 6.46 | | 7.01 | | | 0.37 | | | 30.39 | | | 9.70 | | | 112.13 | | | 40.36 | | 174.17 | | 35.61 | | 328.02 | | 54.56 | | 1160.10 | | 4.80 | | 10464.24 | | 1.77 | |  |
| SBY-2-R-1-61 | 0.12 | | 34.71 | | 0.40 | | 6.19 | | 8.72 | | | 0.18 | | | 37.37 | | | 10.05 | | | 113.54 | | | 40.72 | | 173.31 | | 36.22 | | 334.96 | | 61.91 | | 1106.71 | | 3.14 | | 7199.07 | | 0.98 | |  |
| SBY-2-R-1-62 | 7.72 | | 145.08 | | 15.67 | | 113.85 | | 100.76 | | | 45.85 | | | 246.31 | | | 74.53 | | | 649.49 | | | 169.49 | | 614.86 | | 117.00 | | 1031.00 | | 150.58 | | 5034.53 | | 2.96 | | 7960.04 | | 0.87 | |  |
| SBY-2-R-1-63 | 0.06 | | 15.82 | | 0.12 | | 1.50 | | 3.39 | | | 0.86 | | | 20.75 | | | 7.33 | | | 87.29 | | | 33.61 | | 148.77 | | 31.99 | | 304.16 | | 52.41 | | 994.62 | | 2.43 | | 11386.01 | | 1.39 | |  |
| SBY-2-R-1-64 | 0.07 | | 15.76 | | 0.13 | | 2.78 | | 5.79 | | | 0.62 | | | 32.63 | | | 11.83 | | | 147.55 | | | 56.76 | | 256.91 | | 55.47 | | 528.47 | | 91.01 | | 1651.71 | | 4.02 | | 11303.79 | | 2.12 | |  |
| SBY-2-R-1-65 | 19.41 | | 68.23 | | 8.21 | | 46.49 | | 13.43 | | | 0.23 | | | 29.64 | | | 8.47 | | | 96.27 | | | 34.20 | | 151.20 | | 31.01 | | 291.10 | | 47.61 | | 961.96 | | 4.94 | | 12611.27 | | 1.84 | |  |
| SBY-2-R-1-66 | 0.05 | | 2.32 | | 0.07 | | 1.72 | | 3.77 | | | 0.26 | | | 25.78 | | | 9.78 | | | 126.06 | | | 51.58 | | 247.04 | | 54.70 | | 521.90 | | 92.17 | | 1488.91 | | 3.93 | | 9941.64 | | 1.43 | |  |
| SBY-2-R-1-67 | 0.04 | | 13.86 | | 0.04 | | 0.60 | | 1.75 | | | 0.52 | | | 10.33 | | | 3.63 | | | 46.20 | | | 18.94 | | 97.53 | | 25.02 | | 279.37 | | 57.92 | | 625.58 | | 2.41 | | 12737.89 | | 1.69 | |  |
| SBY-2-R-1-68 | 0.07 | | 17.49 | | 0.12 | | 1.72 | | 2.97 | | | 0.87 | | | 14.84 | | | 5.88 | | | 75.36 | | | 32.00 | | 170.66 | | 44.71 | | 517.22 | | 105.95 | | 1082.48 | | 3.20 | | 11770.83 | | 1.92 | |  |
| SBY-2-R-1-69 | 0.05 | | 5.08 | | 0.07 | | 1.50 | | 3.03 | | | 0.50 | | | 16.45 | | | 6.11 | | | 77.87 | | | 30.11 | | 134.55 | | 30.03 | | 294.93 | | 51.01 | | 854.34 | | 1.92 | | 11500.01 | | 1.36 | |  |
| SBY-2-R-1-70 | 0.15 | | 4.02 | | 0.25 | | 4.75 | | 10.29 | | | 0.33 | | | 58.22 | | | 19.89 | | | 232.15 | | | 83.04 | | 362.15 | | 73.03 | | 655.79 | | 106.89 | | 2347.38 | | 1.19 | | 12375.39 | | 0.80 | |  |
| SBY-2-R-1-71 | 0.02 | | 12.07 | | 0.05 | | 1.24 | | 2.56 | | | 0.57 | | | 14.85 | | | 5.45 | | | 69.49 | | | 28.87 | | 143.53 | | 32.93 | | 345.47 | | 64.97 | | 892.45 | | 1.68 | | 11778.78 | | 0.81 | |  |
| SBY-2-R-1-72 | 0.00 | | 26.77 | | 0.09 | | 2.02 | | 5.55 | | | 0.31 | | | 38.88 | | | 16.13 | | | 216.68 | | | 81.03 | | 363.16 | | 74.26 | | 663.30 | | 96.23 | | 2151.56 | | 53.43 | | 13839.23 | | 19.57 | |  |
| SBY-2-R-1-73 | 0.55 | | 19.90 | | 0.36 | | 3.32 | | 3.58 | | | 0.95 | | | 14.42 | | | 5.15 | | | 58.17 | | | 21.62 | | 99.61 | | 22.68 | | 232.62 | | 42.16 | | 669.42 | | 1.15 | | 12102.18 | | 0.58 | |  |
| SBY-2-R-1-74 | 0.07 | | 21.48 | | 0.20 | | 3.47 | | 6.36 | | | 1.01 | | | 37.36 | | | 12.66 | | | 160.23 | | | 58.99 | | 270.63 | | 58.37 | | 566.99 | | 96.39 | | 1768.51 | | 3.34 | | 10058.36 | | 1.62 | |  |
| SBY-2-R-1-75 | 0.70 | | 9.46 | | 0.31 | | 3.56 | | 4.89 | | | 0.85 | | | 24.43 | | | 8.28 | | | 95.13 | | | 37.47 | | 180.08 | | 40.65 | | 426.60 | | 78.38 | | 1168.06 | | 1.83 | | 11758.67 | | 0.70 | |  |
| SBY-2-R-1-76 | 2.05 | | 36.20 | | 1.79 | | 14.08 | | 11.18 | | | 1.48 | | | 44.90 | | | 14.68 | | | 175.72 | | | 63.03 | | 292.97 | | 63.26 | | 599.52 | | 94.86 | | 1917.69 | | 9.22 | | 10273.22 | | 3.72 | |  |
| SBY-2-R-1-77 | 1.32 | | 10.03 | | 0.83 | | 6.19 | | 6.47 | | | 5.97 | | | 21.32 | | | 6.26 | | | 62.58 | | | 19.73 | | 88.77 | | 21.11 | | 243.01 | | 43.99 | | 599.68 | | 10.79 | | 12557.73 | | 19.38 | |  |
| SBY-2-R-1-78 | 0.05 | | 6.49 | | 0.11 | | 2.02 | | 3.96 | | | 0.29 | | | 21.46 | | | 6.64 | | | 80.54 | | | 28.88 | | 129.01 | | 26.97 | | 268.31 | | 44.83 | | 855.01 | | 1.34 | | 11120.10 | | 0.69 | |  |
| SBY-2-R-1-79 | 14.52 | | 43.42 | | 2.01 | | 24.00 | | 0.00 | | | 5.45 | | | 0.00 | | | 2.79 | | | 3.49 | | | 1.16 | | 0.00 | | 0.00 | | 0.00 | | 0.00 | | 9.60 | | 0.00 | | 2.94 | | 0.00 | |  |
| SBY-2-R-1-80 | 4.57 | | 55.77 | | 1.62 | | 14.06 | | 14.40 | | | 2.88 | | | 51.36 | | | 14.38 | | | 157.22 | | | 52.45 | | 228.49 | | 48.58 | | 469.68 | | 76.42 | | 1597.57 | | 2.60 | | 11706.31 | | 1.48 | |  |
| SBY-2-R-1-81 | 0.71 | | 65.98 | | 0.96 | | 5.92 | | 4.18 | | | 1.69 | | | 11.93 | | | 3.53 | | | 39.56 | | | 16.20 | | 85.23 | | 23.09 | | 282.38 | | 61.06 | | 601.35 | | 4.19 | | 12580.76 | | 0.82 | |  |
| SBY-2-R-1-82 | 0.03 | | 7.87 | | 0.04 | | 0.91 | | 2.02 | | | 0.03 | | | 10.65 | | | 3.95 | | | 49.41 | | | 17.94 | | 83.59 | | 17.64 | | 173.55 | | 28.05 | | 545.28 | | 2.43 | | 13207.60 | | 1.57 | |  |
| SBY-2-R-1-83 | 0.77 | | 17.25 | | 0.48 | | 4.19 | | 3.86 | | | 0.81 | | | 21.79 | | | 6.38 | | | 72.72 | | | 26.37 | | 118.66 | | 25.85 | | 256.48 | | 45.93 | | 799.37 | | 1.33 | | 12724.25 | | 0.71 | |  |
| SBY-2-R-1-84 | 0.00 | | 6.91 | | 0.00 | | 0.32 | | 0.82 | | | 0.01 | | | 6.55 | | | 2.80 | | | 40.90 | | | 17.41 | | 84.91 | | 19.56 | | 203.54 | | 35.38 | | 523.96 | | 3.14 | | 12499.64 | | 2.76 | |  |
| SBY-2-R-1-85 | 1.09 | | 19.30 | | 2.52 | | 17.02 | | 14.47 | | | 4.98 | | | 33.05 | | | 10.82 | | | 109.88 | | | 35.37 | | 152.70 | | 30.69 | | 283.53 | | 50.72 | | 978.67 | | 2.77 | | 10530.63 | | 0.86 | |  |
| SBY-2-R-1-86 | 3.78 | | 10.26 | | 0.80 | | 5.44 | | 6.35 | | | 2.67 | | | 27.74 | | | 9.44 | | | 107.55 | | | 36.22 | | 172.68 | | 43.13 | | 471.56 | | 82.02 | | 1101.21 | | 2.46 | | 12653.86 | | 2.10 | |  |
| SBY-2-R-1-87 | 0.26 | | 12.32 | | 0.50 | | 4.77 | | 8.43 | | | 1.45 | | | 39.20 | | | 14.33 | | | 175.74 | | | 65.20 | | 291.33 | | 61.88 | | 590.67 | | 97.49 | | 1855.28 | | 2.71 | | 11604.73 | | 1.32 | |  |
| SBY-2-R-1-88 | 0.09 | | 30.15 | | 0.85 | | 14.72 | | 23.00 | | | 8.34 | | | 89.40 | | | 23.47 | | | 235.75 | | | 72.19 | | 286.86 | | 56.49 | | 508.46 | | 79.95 | | 2062.19 | | 3.11 | | 10605.00 | | 1.03 | |  |
| SBY-2-R-1-89 | 1.45 | | 38.20 | | 1.07 | | 7.01 | | 4.70 | | | 3.04 | | | 15.59 | | | 4.20 | | | 47.05 | | | 18.09 | | 89.13 | | 21.47 | | 251.33 | | 49.85 | | 613.31 | | 1.15 | | 10255.88 | | 0.51 | |  |
| SBY-2-R-1-90 | 0.00 | | 14.91 | | 0.10 | | 2.65 | | 5.63 | | | 0.59 | | | 27.77 | | | 8.59 | | | 95.40 | | | 31.65 | | 124.95 | | 24.16 | | 219.28 | | 32.55 | | 863.23 | | 12.83 | | 10038.69 | | 4.47 | |  |
| JF-39-R-1 | |  | |  | |  | |  | |  | | |  | | |  | | |  | | |  | | |  | |  | |  | |  | |  | |  | |  | |  | |  | |
| JF-39-R-1-01 | 0.03 | | 1.50 | | 0.06 | | 1.17 | | 3.14 | | | 0.19 | | | 24.94 | | | 10.24 | | | 141.54 | | | 57.21 | | 271.31 | | 59.41 | | 550.05 | | 110.08 | | 1711.64 | | 1.11 | | 11019.58 | | 1.00 | |  |
| JF-39-R-1-02 | 0.07 | | 2.80 | | 1.19 | | 4.65 | | 11.20 | | | 0.39 | | | 50.23 | | | 18.42 | | | 224.72 | | | 85.71 | | 387.09 | | 78.64 | | 695.39 | | 132.26 | | 2562.48 | | 2.29 | | 11018.68 | | 0.77 | |  |
| JF-39-R-1-03 | 0.12 | | 3.22 | | 0.39 | | 3.40 | | 4.89 | | | 0.55 | | | 24.96 | | | 9.30 | | | 118.70 | | | 44.43 | | 200.47 | | 42.59 | | 386.24 | | 77.31 | | 1356.85 | | 1.55 | | 11110.48 | | 0.60 | |  |
| JF-39-R-1-04 | 0.00 | | 1.61 | | 0.09 | | 2.87 | | 8.00 | | | 0.16 | | | 55.99 | | | 20.52 | | | 254.43 | | | 97.76 | | 425.61 | | 84.53 | | 730.58 | | 138.05 | | 2885.72 | | 0.72 | | 10699.70 | | 0.55 | |  |
| JF-39-R-1-05 | 0.05 | | 1.17 | | 0.13 | | 2.24 | | 6.36 | | | 0.14 | | | 51.69 | | | 22.73 | | | 306.84 | | | 123.47 | | 561.14 | | 115.90 | | 1027.13 | | 195.48 | | 3627.38 | | 1.23 | | 11206.45 | | 0.81 | |  |
| JF-39-R-1-06 | 0.01 | | 1.01 | | 0.07 | | 1.24 | | 5.04 | | | 0.08 | | | 37.08 | | | 16.28 | | | 223.01 | | | 89.03 | | 417.13 | | 87.86 | | 785.90 | | 150.44 | | 2681.82 | | 0.96 | | 11184.93 | | 0.63 | |  |
| JF-39-R-1-07 | 0.02 | | 0.67 | | 0.04 | | 0.95 | | 3.13 | | | 0.07 | | | 25.20 | | | 10.92 | | | 151.54 | | | 63.36 | | 302.41 | | 64.79 | | 596.02 | | 115.39 | | 1899.98 | | 0.79 | | 11629.15 | | 0.56 | |  |
| JF-39-R-1-08 | 0.08 | | 22.14 | | 0.13 | | 2.59 | | 4.90 | | | 1.06 | | | 26.69 | | | 8.78 | | | 98.37 | | | 39.28 | | 171.24 | | 35.21 | | 313.66 | | 63.97 | | 1126.62 | | 1.83 | | 8458.44 | | 0.72 | |  |
| JF-39-R-1-09 | 0.06 | | 1.06 | | 0.09 | | 1.88 | | 4.58 | | | 0.13 | | | 33.66 | | | 14.62 | | | 196.49 | | | 80.62 | | 370.70 | | 78.19 | | 698.67 | | 138.97 | | 2377.95 | | 0.91 | | 11061.77 | | 0.67 | |  |
| JF-39-R-1-10 | 0.01 | | 1.23 | | 0.08 | | 1.43 | | 4.64 | | | 0.05 | | | 39.13 | | | 16.61 | | | 220.91 | | | 88.49 | | 411.55 | | 83.46 | | 740.88 | | 142.01 | | 2601.32 | | 0.91 | | 11231.81 | | 0.51 | |  |
| JF-39-R-1-11 | 0.12 | | 3.11 | | 0.49 | | 6.06 | | 13.13 | | | 1.47 | | | 57.96 | | | 19.38 | | | 241.05 | | | 90.80 | | 398.60 | | 83.75 | | 742.54 | | 141.52 | | 2639.01 | | 1.07 | | 11347.35 | | 0.74 | |  |
| JF-39-R-1-12 | 0.01 | | 1.76 | | 0.08 | | 1.24 | | 4.15 | | | 0.11 | | | 30.14 | | | 12.69 | | | 170.27 | | | 70.32 | | 316.59 | | 66.54 | | 603.52 | | 115.41 | | 2061.44 | | 1.18 | | 10616.93 | | 0.67 | |  |
| JF-39-R-1-13 | 0.02 | | 1.23 | | 0.15 | | 2.64 | | 5.65 | | | 0.31 | | | 35.51 | | | 15.03 | | | 204.46 | | | 80.57 | | 371.25 | | 78.73 | | 705.58 | | 136.64 | | 2407.21 | | 1.11 | | 11308.31 | | 0.61 | |  |
| JF-39-R-1-14 | 0.04 | | 0.90 | | 0.09 | | 1.57 | | 5.61 | | | 0.15 | | | 41.31 | | | 16.84 | | | 189.48 | | | 61.14 | | 244.08 | | 46.53 | | 407.27 | | 76.07 | | 1866.32 | | 1.01 | | 11582.46 | | 0.59 | |  |
| JF-39-R-1-15 | 0.17 | | 1.86 | | 0.26 | | 3.72 | | 8.99 | | | 0.28 | | | 68.99 | | | 30.39 | | | 404.34 | | | 159.82 | | 725.88 | | 148.57 | | 1306.91 | | 247.37 | | 3911.97 | | 0.83 | | 11167.77 | | 0.71 | |  |
| JF-39-R-1-16 | 0.01 | | 1.33 | | 0.09 | | 1.81 | | 6.00 | | | 0.12 | | | 46.03 | | | 20.05 | | | 263.22 | | | 105.45 | | 481.69 | | 97.91 | | 873.61 | | 169.06 | | 3109.15 | | 1.03 | | 11081.14 | | 0.76 | |  |
| JF-39-R-1-17 | 0.01 | | 5.02 | | 0.07 | | 1.50 | | 3.13 | | | 0.25 | | | 21.39 | | | 7.28 | | | 88.28 | | | 34.44 | | 152.22 | | 31.35 | | 282.65 | | 56.82 | | 978.89 | | 4.30 | | 10473.91 | | 1.89 | |  |
| JF-39-R-1-18 | 0.00 | | 0.95 | | 0.05 | | 0.88 | | 3.14 | | | 0.02 | | | 26.40 | | | 11.20 | | | 151.10 | | | 62.21 | | 294.80 | | 61.98 | | 561.65 | | 110.62 | | 1866.56 | | 0.68 | | 11338.00 | | 0.53 | |  |
| JF-39-R-1-19 | 0.01 | | 1.10 | | 0.01 | | 1.01 | | 3.74 | | | 0.08 | | | 28.68 | | | 11.59 | | | 151.65 | | | 61.36 | | 278.90 | | 57.40 | | 519.38 | | 101.98 | | 1821.75 | | 0.74 | | 11399.11 | | 0.52 | |  |
| JF-39-R-1-20 | 0.04 | | 0.99 | | 0.11 | | 0.85 | | 3.22 | | | 0.13 | | | 20.30 | | | 8.43 | | | 120.09 | | | 50.49 | | 244.43 | | 52.34 | | 492.28 | | 97.89 | | 1552.72 | | 0.63 | | 12437.38 | | 0.41 | |  |
| JF-39-R-1-21 | 0.02 | | 0.91 | | 0.06 | | 1.33 | | 5.22 | | | 0.02 | | | 49.58 | | | 25.70 | | | 382.68 | | | 167.86 | | 820.98 | | 178.39 | | 1620.82 | | 315.04 | | 4992.11 | | 1.74 | | 11671.25 | | 1.47 | |  |
| JF-39-R-1-22 | 0.00 | | 0.99 | | 0.05 | | 1.02 | | 3.99 | | | 0.03 | | | 29.74 | | | 13.45 | | | 187.10 | | | 77.43 | | 361.76 | | 75.63 | | 698.98 | | 135.24 | | 2326.81 | | 0.96 | | 11525.04 | | 0.64 | |  |
| JF-39-R-1-23 | 2.16 | | 9.79 | | 2.59 | | 34.48 | | 55.97 | | | 6.12 | | | 170.79 | | | 48.88 | | | 456.52 | | | 142.97 | | 566.62 | | 109.21 | | 939.68 | | 177.43 | | 3697.89 | | 2.25 | | 11571.01 | | 0.69 | |  |
| JF-39-R-1-24 | 0.02 | | 1.48 | | 0.11 | | 1.99 | | 5.62 | | | 0.09 | | | 42.05 | | | 15.63 | | | 196.09 | | | 76.57 | | 342.73 | | 69.04 | | 623.83 | | 122.13 | | 2298.70 | | 1.08 | | 10950.39 | | 0.50 | |  |
| JF-39-R-1-25 | 0.00 | | 2.07 | | 0.04 | | 1.20 | | 4.20 | | | 0.09 | | | 23.51 | | | 10.26 | | | 127.64 | | | 52.46 | | 241.24 | | 50.87 | | 455.45 | | 90.74 | | 1561.12 | | 1.25 | | 10916.88 | | 0.83 | |  |
| JF-39-R-1-26 | 0.02 | | 1.72 | | 0.05 | | 1.49 | | 4.22 | | | 0.08 | | | 28.38 | | | 12.09 | | | 156.04 | | | 63.04 | | 282.88 | | 58.46 | | 531.72 | | 103.66 | | 1867.92 | | 1.06 | | 11761.43 | | 0.59 | |  |
| JF-39-R-1-27 | 0.03 | | 0.95 | | 0.04 | | 0.86 | | 2.79 | | | 0.04 | | | 18.51 | | | 8.40 | | | 119.61 | | | 50.50 | | 242.79 | | 51.37 | | 478.09 | | 94.07 | | 1529.84 | | 0.77 | | 12381.15 | | 0.51 | |  |
| JF-39-R-1-28 | 0.01 | | 1.75 | | 0.20 | | 3.51 | | 7.96 | | | 0.11 | | | 52.50 | | | 19.82 | | | 250.52 | | | 99.18 | | 440.00 | | 88.08 | | 789.15 | | 149.91 | | 2855.45 | | 1.08 | | 11103.27 | | 0.67 | |  |
| JF-39-R-1-29 | 0.71 | | 2.66 | | 0.32 | | 2.14 | | 3.53 | | | 0.04 | | | 23.73 | | | 9.79 | | | 129.21 | | | 54.81 | | 246.06 | | 51.53 | | 460.17 | | 89.78 | | 1616.13 | | 0.84 | | 11431.52 | | 0.46 | |  |
| JF-39-R-1-30 | 0.00 | | 0.84 | | 0.04 | | 1.09 | | 2.99 | | | 0.06 | | | 28.61 | | | 12.73 | | | 168.68 | | | 71.71 | | 331.95 | | 69.89 | | 629.82 | | 122.13 | | 2125.18 | | 0.90 | | 11797.76 | | 0.64 | |  |
| JF-39-R-1-31 | 0.02 | | 2.41 | | 0.25 | | 4.03 | | 9.56 | | | 0.40 | | | 51.59 | | | 17.87 | | | 206.74 | | | 75.23 | | 317.48 | | 63.13 | | 557.94 | | 108.69 | | 2201.20 | | 1.16 | | 10048.73 | | 0.73 | |  |
| JF-39-R-1-32 | 0.03 | | 1.75 | | 0.28 | | 4.13 | | 7.95 | | | 0.36 | | | 43.74 | | | 16.72 | | | 201.42 | | | 77.20 | | 338.02 | | 67.69 | | 605.99 | | 117.11 | | 2257.43 | | 0.96 | | 11046.09 | | 0.49 | |  |
| JF-39-R-1-33 | 0.00 | | 1.11 | | 0.08 | | 1.33 | | 5.36 | | | 0.12 | | | 40.55 | | | 16.95 | | | 232.09 | | | 97.10 | | 449.16 | | 92.17 | | 832.00 | | 159.84 | | 2881.77 | | 1.24 | | 11244.94 | | 0.70 | |  |
| JF-39-R-1-34 | 0.01 | | 4.87 | | 0.09 | | 1.66 | | 4.19 | | | 0.17 | | | 28.20 | | | 10.37 | | | 131.08 | | | 52.04 | | 234.10 | | 47.89 | | 440.65 | | 88.21 | | 1492.86 | | 3.83 | | 11206.43 | | 1.74 | |  |
| JF-39-R-1-35 | 0.01 | | 1.09 | | 0.07 | | 1.33 | | 5.13 | | | 0.05 | | | 40.15 | | | 17.48 | | | 226.76 | | | 92.57 | | 427.58 | | 88.59 | | 788.22 | | 152.78 | | 2789.10 | | 1.15 | | 11291.28 | | 0.91 | |  |
| JF-39-R-1-36 | 0.01 | | 0.79 | | 0.03 | | 0.89 | | 4.19 | | | 0.09 | | | 35.52 | | | 15.65 | | | 210.11 | | | 86.48 | | 405.73 | | 83.89 | | 748.32 | | 145.77 | | 2569.54 | | 0.80 | | 11775.54 | | 0.61 | |  |
| JF-39-R-1-37 | 0.00 | | 0.96 | | 0.03 | | 0.93 | | 3.15 | | | 0.08 | | | 24.39 | | | 10.95 | | | 147.12 | | | 62.18 | | 291.00 | | 60.75 | | 553.75 | | 109.02 | | 1876.71 | | 0.80 | | 11602.63 | | 0.58 | |  |
| JF-39-R-1-38 | 0.11 | | 16.99 | | 0.39 | | 3.98 | | 6.65 | | | 1.55 | | | 27.65 | | | 10.04 | | | 117.99 | | | 45.04 | | 200.83 | | 41.41 | | 384.35 | | 74.19 | | 1355.58 | | 1.89 | | 10891.59 | | 0.85 | |  |
| JF-39-R-1-39 | 0.02 | | 1.08 | | 0.08 | | 1.04 | | 4.43 | | | 0.10 | | | 38.09 | | | 16.00 | | | 211.07 | | | 85.79 | | 399.05 | | 80.38 | | 725.82 | | 141.12 | | 2542.07 | | 0.86 | | 11160.72 | | 0.63 | |  |
| JF-39-R-1-40 | 0.00 | | 0.90 | | 0.02 | | 0.84 | | 4.11 | | | 0.11 | | | 31.53 | | | 13.71 | | | 187.27 | | | 77.05 | | 353.63 | | 73.79 | | 665.33 | | 132.57 | | 2272.33 | | 0.77 | | 11498.97 | | 0.55 | |  |
| JF-39-R-1-41 | 0.00 | | 1.14 | | 0.09 | | 1.55 | | 4.83 | | | 0.07 | | | 37.53 | | | 15.45 | | | 204.39 | | | 84.63 | | 382.89 | | 79.84 | | 710.14 | | 140.52 | | 2470.40 | | 0.91 | | 11064.25 | | 0.67 | |  |
| JF-39-R-1-42 | 0.45 | | 11.99 | | 2.22 | | 28.82 | | 54.25 | | | 6.97 | | | 147.53 | | | 43.71 | | | 376.43 | | | 121.75 | | 472.04 | | 94.03 | | 812.60 | | 147.77 | | 3384.91 | | 1.40 | | 11878.70 | | 0.78 | |  |
| JF-39-R-1-43 | 0.01 | | 1.35 | | 0.22 | | 2.43 | | 6.79 | | | 0.22 | | | 52.59 | | | 24.11 | | | 340.12 | | | 145.71 | | 696.24 | | 147.51 | | 1333.18 | | 255.95 | | 4371.55 | | 1.56 | | 11603.04 | | 1.17 | |  |
| JF-39-R-1-44 | 0.00 | | 0.83 | | 0.04 | | 0.73 | | 2.82 | | | 0.03 | | | 23.50 | | | 9.45 | | | 124.06 | | | 50.37 | | 239.46 | | 50.43 | | 449.23 | | 89.09 | | 1540.12 | | 0.47 | | 11529.56 | | 0.43 | |  |
| JF-39-R-1-45 | 0.01 | | 8.72 | | 0.08 | | 1.41 | | 3.61 | | | 0.18 | | | 25.62 | | | 9.96 | | | 129.62 | | | 51.36 | | 233.91 | | 47.97 | | 440.57 | | 86.23 | | 1503.49 | | 4.61 | | 11040.10 | | 2.61 | |  |
| JF-39-R-1-46 | 0.01 | | 1.29 | | 0.05 | | 1.06 | | 4.14 | | | 0.11 | | | 29.92 | | | 11.50 | | | 153.10 | | | 61.95 | | 275.44 | | 57.17 | | 501.06 | | 98.71 | | 1809.30 | | 0.74 | | 10888.31 | | 0.35 | |  |
| JF-39-R-1-47 | 0.00 | | 1.24 | | 0.04 | | 1.16 | | 3.81 | | | 0.13 | | | 29.41 | | | 12.11 | | | 161.04 | | | 65.85 | | 308.54 | | 63.62 | | 575.23 | | 112.01 | | 1990.29 | | 0.77 | | 10940.07 | | 0.65 | |  |
| JF-39-R-1-48 | 0.26 | | 0.83 | | 0.05 | | 0.70 | | 3.17 | | | 0.02 | | | 30.03 | | | 12.79 | | | 175.35 | | | 73.98 | | 349.59 | | 72.71 | | 663.47 | | 130.66 | | 2241.34 | | 0.92 | | 11428.71 | | 0.61 | |  |
| JF-39-R-1-49 | 0.02 | | 1.32 | | 0.12 | | 1.84 | | 5.34 | | | 0.06 | | | 38.75 | | | 15.50 | | | 188.00 | | | 74.94 | | 335.47 | | 68.05 | | 606.95 | | 115.89 | | 2249.19 | | 0.72 | | 10709.78 | | 0.45 | |  |
| JF-39-R-1-50 | 0.00 | | 0.87 | | 0.10 | | 1.80 | | 6.12 | | | 0.07 | | | 47.43 | | | 21.74 | | | 304.90 | | | 125.03 | | 584.61 | | 122.72 | | 1085.95 | | 209.50 | | 3752.91 | | 1.21 | | 10899.91 | | 0.87 | |  |
| JF-39-R-1-51 | 0.06 | | 1.44 | | 0.27 | | 2.85 | | 7.15 | | | 0.54 | | | 35.70 | | | 15.30 | | | 194.30 | | | 79.58 | | 362.19 | | 75.54 | | 685.04 | | 135.35 | | 2380.00 | | 1.15 | | 11367.94 | | 0.63 | |  |
| JF-39-R-1-52 | 3.61 | | 20.03 | | 1.41 | | 10.08 | | 10.03 | | | 1.58 | | | 41.39 | | | 14.04 | | | 178.01 | | | 67.93 | | 319.32 | | 68.80 | | 659.30 | | 134.72 | | 2101.95 | | 3.28 | | 10791.62 | | 1.46 | |  |
| JF-39-R-1-53 | 0.01 | | 1.11 | | 0.07 | | 1.37 | | 5.56 | | | 0.08 | | | 42.60 | | | 17.41 | | | 233.27 | | | 93.28 | | 424.29 | | 89.74 | | 799.56 | | 155.94 | | 2768.49 | | 1.14 | | 11391.56 | | 0.57 | |  |
| JF-39-R-1-54 | 0.01 | | 1.00 | | 0.08 | | 1.19 | | 4.55 | | | 0.07 | | | 35.44 | | | 15.83 | | | 215.77 | | | 88.52 | | 414.40 | | 86.89 | | 778.98 | | 151.68 | | 2690.10 | | 0.91 | | 11162.69 | | 0.66 | |  |
| JF-39-R-1-55 | 0.21 | | 1.11 | | 0.09 | | 0.78 | | 2.80 | | | 0.03 | | | 25.60 | | | 11.50 | | | 160.61 | | | 68.18 | | 319.78 | | 68.02 | | 619.68 | | 120.86 | | 2066.42 | | 0.84 | | 11581.70 | | 0.58 | |  |
| JF-39-R-1-56 | 0.00 | | 1.32 | | 0.07 | | 1.67 | | 5.10 | | | 0.12 | | | 34.30 | | | 13.37 | | | 168.81 | | | 67.88 | | 303.14 | | 62.77 | | 570.34 | | 113.16 | | 2006.86 | | 0.79 | | 11226.25 | | 0.53 | |  |
| JF-39-R-1-57 | 0.01 | | 0.92 | | 0.07 | | 1.35 | | 3.47 | | | 0.08 | | | 29.73 | | | 13.32 | | | 186.32 | | | 77.03 | | 350.92 | | 74.26 | | 680.11 | | 133.78 | | 2306.67 | | 1.09 | | 11701.22 | | 0.56 | |  |
| JF-39-R-1-58 | 0.01 | | 1.07 | | 0.06 | | 1.64 | | 5.17 | | | 0.05 | | | 40.08 | | | 16.39 | | | 214.46 | | | 86.48 | | 386.38 | | 79.24 | | 697.20 | | 135.67 | | 2573.07 | | 0.84 | | 10953.58 | | 0.53 | |  |
| JF-39-R-1-59 | 0.05 | | 2.08 | | 0.23 | | 4.05 | | 9.01 | | | 0.22 | | | 53.39 | | | 18.21 | | | 212.97 | | | 81.27 | | 354.06 | | 69.59 | | 618.06 | | 120.98 | | 2348.85 | | 0.79 | | 10600.18 | | 0.50 | |  |
| JF-39-R-1-60 | 0.01 | | 1.26 | | 0.10 | | 1.93 | | 5.33 | | | 0.06 | | | 40.31 | | | 17.71 | | | 227.70 | | | 93.02 | | 419.34 | | 88.82 | | 793.86 | | 154.84 | | 2777.78 | | 1.05 | | 11211.84 | | 0.67 | |  |
| JF-39-R-1-61 | 0.28 | | 1.63 | | 0.10 | | 1.99 | | 5.46 | | | 0.06 | | | 41.05 | | | 18.02 | | | 242.74 | | | 99.62 | | 455.37 | | 96.00 | | 849.23 | | 165.58 | | 2935.62 | | 1.33 | | 11026.21 | | 0.65 | |  |
| JF-39-R-1-62 | 0.06 | | 1.13 | | 0.12 | | 2.50 | | 5.87 | | | 0.28 | | | 41.66 | | | 18.41 | | | 248.40 | | | 101.64 | | 472.56 | | 98.15 | | 879.32 | | 171.37 | | 3027.86 | | 0.91 | | 11418.77 | | 0.70 | |  |
| JF-39-R-1-63 | 0.01 | | 0.91 | | 0.07 | | 1.08 | | 2.63 | | | 0.06 | | | 26.16 | | | 11.56 | | | 158.79 | | | 66.54 | | 303.44 | | 64.92 | | 592.27 | | 114.84 | | 1998.10 | | 0.71 | | 12188.29 | | 0.63 | |  |
| JF-39-R-1-64 | 2.82 | | 8.80 | | 0.93 | | 4.29 | | 3.85 | | | 0.09 | | | 27.08 | | | 11.28 | | | 140.78 | | | 56.84 | | 259.22 | | 54.72 | | 487.99 | | 95.73 | | 1730.73 | | 1.95 | | 12530.82 | | 1.37 | |  |
| JF-39-R-1-65 | 0.00 | | 1.12 | | 0.07 | | 1.29 | | 4.09 | | | 0.11 | | | 34.24 | | | 14.70 | | | 190.10 | | | 79.81 | | 371.23 | | 78.83 | | 690.92 | | 134.01 | | 2406.60 | | 1.04 | | 11143.46 | | 0.58 | |  |
| JF-39-R-1-66 | 0.06 | | 7.03 | | 0.04 | | 0.29 | | 0.82 | | | 0.06 | | | 5.22 | | | 1.96 | | | 25.94 | | | 12.16 | | 62.96 | | 14.98 | | 153.04 | | 32.27 | | 384.87 | | 2.57 | | 11577.22 | | 2.15 | |  |
| JF-39-R-1-67 | 0.37 | | 16.48 | | 2.26 | | 28.22 | | 55.72 | | | 7.20 | | | 144.09 | | | 34.55 | | | 279.16 | | | 75.14 | | 268.64 | | 47.19 | | 396.68 | | 71.67 | | 2152.44 | | 2.41 | | 10394.83 | | 1.39 | |  |
| JF-39-R-1-68 | 0.02 | | 2.82 | | 0.07 | | 1.44 | | 2.61 | | | 0.36 | | | 15.91 | | | 5.54 | | | 70.10 | | | 26.34 | | 115.64 | | 23.42 | | 216.24 | | 43.79 | | 745.66 | | 2.35 | | 9623.75 | | 0.86 | |  |
| JF-39-R-1-69 | 0.00 | | 2.27 | | 0.07 | | 0.95 | | 2.33 | | | 0.15 | | | 17.45 | | | 5.89 | | | 76.80 | | | 29.88 | | 134.88 | | 27.69 | | 248.07 | | 48.73 | | 869.37 | | 0.83 | | 10456.52 | | 0.61 | |  |
| JF-39-R-1-70 | 0.00 | | 1.31 | | 0.09 | | 1.57 | | 4.76 | | | 0.07 | | | 38.51 | | | 15.90 | | | 211.74 | | | 85.08 | | 391.18 | | 80.67 | | 718.75 | | 138.51 | | 2535.55 | | 1.14 | | 10950.32 | | 0.64 | |  |
| JF-39-R-1-71 | 0.01 | | 0.87 | | 0.03 | | 1.34 | | 3.84 | | | 0.05 | | | 30.22 | | | 12.33 | | | 164.87 | | | 66.79 | | 303.16 | | 62.43 | | 558.36 | | 106.97 | | 1989.74 | | 0.73 | | 11290.65 | | 0.41 | |  |
| JF-39-R-1-72 | 0.01 | | 1.25 | | 0.07 | | 1.11 | | 3.62 | | | 0.08 | | | 25.12 | | | 10.76 | | | 141.94 | | | 57.66 | | 270.99 | | 56.34 | | 508.49 | | 99.31 | | 1737.42 | | 0.85 | | 11159.08 | | 0.55 | |  |
| JF-39-R-1-73 | 0.02 | | 1.01 | | 0.05 | | 1.28 | | 5.01 | | | 0.07 | | | 34.97 | | | 15.64 | | | 207.72 | | | 82.94 | | 385.94 | | 79.83 | | 696.82 | | 134.38 | | 2508.88 | | 0.92 | | 11302.08 | | 0.59 | |  |
| JF-39-R-1-74 | 0.00 | | 0.77 | | 0.08 | | 1.30 | | 4.23 | | | 0.08 | | | 31.35 | | | 13.25 | | | 180.02 | | | 73.66 | | 342.74 | | 71.68 | | 647.45 | | 127.64 | | 2211.84 | | 0.93 | | 11578.12 | | 0.57 | |  |
| JF-39-R-1-75 | 0.10 | | 1.46 | | 0.22 | | 3.15 | | 8.30 | | | 0.84 | | | 41.45 | | | 16.64 | | | 225.83 | | | 89.07 | | 415.45 | | 86.88 | | 776.47 | | 151.21 | | 2659.90 | | 1.08 | | 11385.85 | | 0.69 | |  |
| JF-39-R-1-76 | 0.01 | | 0.90 | | 0.06 | | 0.97 | | 2.95 | | | 0.01 | | | 22.92 | | | 10.65 | | | 139.04 | | | 57.22 | | 268.06 | | 55.44 | | 497.38 | | 96.71 | | 1697.20 | | 0.67 | | 11584.51 | | 0.49 | |  |
| JF-39-R-1-77 | 0.01 | | 0.89 | | 0.03 | | 0.83 | | 3.32 | | | 0.06 | | | 28.18 | | | 12.70 | | | 174.15 | | | 71.92 | | 334.60 | | 71.43 | | 636.42 | | 124.60 | | 2122.18 | | 1.01 | | 11578.88 | | 0.67 | |  |
| JF-39-R-1-78 | 0.00 | | 0.84 | | 0.04 | | 1.26 | | 3.96 | | | 0.04 | | | 31.44 | | | 13.98 | | | 187.42 | | | 79.31 | | 372.62 | | 77.90 | | 712.60 | | 137.74 | | 2343.93 | | 0.78 | | 11360.82 | | 0.58 | |  |
| JF-39-R-1-79 | 0.00 | | 1.55 | | 0.06 | | 1.24 | | 2.90 | | | 0.05 | | | 20.98 | | | 9.55 | | | 127.32 | | | 50.85 | | 239.04 | | 50.53 | | 446.74 | | 87.14 | | 1532.54 | | 1.17 | | 11442.72 | | 0.81 | |  |
| JF-39-R-1-80 | 0.00 | | 0.70 | | 0.03 | | 0.75 | | 2.58 | | | 0.08 | | | 19.69 | | | 8.56 | | | 113.43 | | | 47.10 | | 224.90 | | 46.91 | | 431.25 | | 85.41 | | 1425.66 | | 0.65 | | 11469.61 | | 0.44 | |  |
| JF-43-R-1 | |  | |  | |  | |  | |  | | |  | | |  | | |  | | |  | | |  | |  | |  | |  | |  | |  | |  | |  | |  | |
| JF-43-R-1-01 | 0.00 | | 15.55 | | 0.05 | | 1.52 | | 4.41 | | | 0.78 | | | 23.79 | | | 8.98 | | | 116.12 | | | 44.95 | | 210.03 | | 45.14 | | 422.78 | | 89.13 | | 1348.18 | | 3.22 | | 10412.53 | | 1.83 | |  |
| JF-43-R-1-02 | 0.02 | | 11.86 | | 0.01 | | 0.76 | | 0.92 | | | 0.30 | | | 5.24 | | | 1.74 | | | 21.66 | | | 9.16 | | 47.98 | | 11.71 | | 126.25 | | 29.49 | | 317.98 | | 0.70 | | 10644.81 | | 0.73 | |  |
| JF-43-R-1-03 | 0.00 | | 10.85 | | 0.04 | | 0.30 | | 0.78 | | | 0.13 | | | 4.02 | | | 1.24 | | | 15.40 | | | 6.44 | | 32.21 | | 8.39 | | 85.04 | | 20.40 | | 221.22 | | 0.71 | | 10709.70 | | 0.77 | |  |
| JF-43-R-1-04 | 0.62 | | 8.53 | | 0.91 | | 7.10 | | 8.72 | | | 3.13 | | | 39.90 | | | 14.89 | | | 168.41 | | | 54.84 | | 212.18 | | 39.79 | | 316.89 | | 57.51 | | 1668.26 | | 0.76 | | 12373.49 | | 0.45 | |  |
| JF-43-R-1-05 | 54.69 | | 248.36 | | 39.24 | | 217.89 | | 119.34 | | | 23.66 | | | 231.17 | | | 65.72 | | | 627.75 | | | 190.31 | | 780.44 | | 173.96 | | 1764.79 | | 352.09 | | 2135.66 | | 8.52 | | 20575.42 | | 6.73 | |  |
| JF-43-R-1-06 | 0.30 | | 1.25 | | 0.33 | | 2.06 | | 2.57 | | | 0.61 | | | 13.11 | | | 5.98 | | | 62.77 | | | 18.31 | | 67.13 | | 12.69 | | 109.14 | | 19.73 | | 585.00 | | 0.66 | | 13206.45 | | 0.82 | |  |
| JF-43-R-1-07 | 0.15 | | 12.01 | | 0.12 | | 1.57 | | 1.78 | | | 0.48 | | | 8.83 | | | 3.09 | | | 37.27 | | | 13.52 | | 64.06 | | 14.35 | | 139.81 | | 29.21 | | 438.39 | | 1.46 | | 10296.33 | | 1.37 | |  |
| JF-43-R-1-08 | 3.78 | | 26.40 | | 1.44 | | 9.47 | | 6.39 | | | 1.03 | | | 23.38 | | | 6.92 | | | 76.96 | | | 26.77 | | 110.75 | | 21.20 | | 188.79 | | 37.15 | | 754.00 | | 2.28 | | 10035.22 | | 0.74 | |  |
| JF-43-R-1-09 | 272.56 | | 1096.66 | | 131.62 | | 617.04 | | 167.40 | | | 72.36 | | | 222.57 | | | 50.75 | | | 453.50 | | | 138.52 | | 547.33 | | 106.40 | | 920.85 | | 172.49 | | 3551.54 | | 11.43 | | 10902.26 | | 1.83 | |  |
| JF-43-R-1-10 | 0.07 | | 29.41 | | 0.40 | | 5.84 | | 9.83 | | | 3.55 | | | 57.32 | | | 18.84 | | | 213.10 | | | 78.73 | | 335.75 | | 68.48 | | 618.55 | | 128.55 | | 2348.04 | | 3.00 | | 8221.16 | | 0.95 | |  |
| JF-43-R-1-11 | 0.14 | | 9.30 | | 0.30 | | 4.58 | | 9.24 | | | 0.68 | | | 51.48 | | | 17.74 | | | 211.50 | | | 79.32 | | 339.19 | | 67.78 | | 585.83 | | 112.53 | | 2289.82 | | 1.65 | | 9726.68 | | 0.84 | |  |
| JF-43-R-1-12 | 3.57 | | 45.18 | | 1.18 | | 6.95 | | 8.41 | | | 2.44 | | | 39.66 | | | 13.60 | | | 161.57 | | | 62.93 | | 296.18 | | 66.38 | | 640.46 | | 135.84 | | 1886.30 | | 3.65 | | 8635.40 | | 1.46 | |  |
| JF-43-R-1-13 | 0.00 | | 22.13 | | 0.08 | | 1.67 | | 3.34 | | | 1.03 | | | 19.03 | | | 6.75 | | | 85.24 | | | 35.68 | | 181.21 | | 42.10 | | 429.41 | | 94.43 | | 1122.78 | | 2.70 | | 9139.61 | | 1.12 | |  |
| JF-43-R-1-14 | 0.02 | | 10.51 | | 0.16 | | 2.68 | | 5.27 | | | 0.18 | | | 27.40 | | | 8.30 | | | 92.20 | | | 33.90 | | 145.95 | | 30.41 | | 270.94 | | 54.65 | | 971.05 | | 1.18 | | 9817.98 | | 0.51 | |  |
| JF-43-R-1-15 | 0.84 | | 10.58 | | 0.57 | | 6.05 | | 9.06 | | | 0.96 | | | 52.18 | | | 17.82 | | | 208.07 | | | 78.48 | | 337.62 | | 67.59 | | 573.74 | | 113.14 | | 2222.78 | | 3.41 | | 9549.62 | | 0.97 | |  |
| JF-43-R-1-16 | 0.07 | | 8.13 | | 0.20 | | 3.54 | | 9.90 | | | 0.36 | | | 57.54 | | | 20.66 | | | 255.85 | | | 98.18 | | 427.99 | | 87.04 | | 766.18 | | 144.99 | | 2788.06 | | 2.80 | | 9885.90 | | 1.52 | |  |
| JF-43-R-1-17 | 0.05 | | 7.14 | | 0.04 | | 1.13 | | 3.46 | | | 0.36 | | | 23.74 | | | 10.24 | | | 137.75 | | | 57.91 | | 278.77 | | 61.07 | | 563.74 | | 112.10 | | 1740.69 | | 2.54 | | 11658.54 | | 2.23 | |  |
| JF-43-R-1-18 | 0.70 | | 15.70 | | 0.53 | | 5.81 | | 6.88 | | | 1.09 | | | 38.29 | | | 14.52 | | | 182.10 | | | 72.38 | | 344.26 | | 75.92 | | 706.98 | | 140.38 | | 2157.82 | | 5.38 | | 10907.66 | | 2.73 | |  |
| JF-43-R-1-19 | 0.00 | | 9.88 | | 0.05 | | 1.25 | | 3.62 | | | 0.22 | | | 28.43 | | | 12.72 | | | 170.24 | | | 69.95 | | 331.62 | | 71.91 | | 653.32 | | 129.49 | | 2073.56 | | 3.42 | | 11583.66 | | 2.78 | |  |
| JF-43-R-1-20 | 0.01 | | 14.97 | | 0.06 | | 1.36 | | 5.12 | | | 0.37 | | | 36.91 | | | 15.74 | | | 208.17 | | | 86.61 | | 408.01 | | 89.33 | | 812.57 | | 159.97 | | 2540.46 | | 4.22 | | 11640.27 | | 3.15 | |  |
| JF-43-R-1-21 | 0.25 | | 9.89 | | 0.09 | | 1.06 | | 1.87 | | | 0.31 | | | 12.75 | | | 4.88 | | | 69.98 | | | 30.82 | | 160.85 | | 38.89 | | 388.83 | | 87.10 | | 994.78 | | 3.40 | | 13178.81 | | 5.00 | |  |
| JF-43-R-1-22 | 13.83 | | 131.48 | | 13.49 | | 68.10 | | 37.95 | | | 10.81 | | | 52.32 | | | 12.62 | | | 107.32 | | | 29.53 | | 124.30 | | 30.15 | | 319.95 | | 75.73 | | 936.39 | | 7.03 | | 13419.55 | | 3.84 | |  |
| JF-43-R-1-23 | 0.03 | | 7.79 | | 0.22 | | 4.69 | | 9.36 | | | 1.27 | | | 55.33 | | | 18.44 | | | 215.05 | | | 82.25 | | 351.28 | | 69.22 | | 608.47 | | 119.40 | | 2309.77 | | 2.44 | | 8163.25 | | 0.90 | |  |
| JF-43-R-1-24 | 0.91 | | 14.81 | | 0.48 | | 4.62 | | 5.14 | | | 1.37 | | | 26.29 | | | 8.86 | | | 111.62 | | | 42.77 | | 194.62 | | 40.47 | | 375.23 | | 76.34 | | 1266.45 | | 1.21 | | 8577.25 | | 0.41 | |  |
| JF-43-R-1-25 | 7.30 | | 97.93 | | 21.45 | | 158.31 | | 138.84 | | | 49.65 | | | 307.76 | | | 78.19 | | | 604.89 | | | 150.61 | | 547.32 | | 111.60 | | 1045.56 | | 201.73 | | 4043.87 | | 3.03 | | 12657.93 | | 1.61 | |  |
| JF-43-R-1-26 | 73.95 | | 155.65 | | 15.62 | | 67.16 | | 11.83 | | | 1.86 | | | 19.98 | | | 6.15 | | | 70.01 | | | 26.79 | | 130.62 | | 28.74 | | 277.92 | | 59.91 | | 816.83 | | 1.26 | | 9042.64 | | 0.45 | |  |
| JF-43-R-1-27 | 0.18 | | 8.78 | | 0.09 | | 1.13 | | 3.46 | | | 0.20 | | | 27.15 | | | 11.16 | | | 152.85 | | | 62.29 | | 295.12 | | 64.11 | | 588.40 | | 116.28 | | 1850.81 | | 3.25 | | 11807.53 | | 2.73 | |  |
| JF-43-R-1-28 | 0.23 | | 2.18 | | 0.06 | | 0.94 | | 1.25 | | | 0.21 | | | 7.37 | | | 2.38 | | | 29.50 | | | 10.47 | | 46.32 | | 9.55 | | 83.08 | | 16.17 | | 303.43 | | 13.85 | | 2664.35 | | 1.23 | |  |
| JF-43-R-1-29 | 0.01 | | 15.83 | | 0.18 | | 3.61 | | 9.27 | | | 0.61 | | | 53.17 | | | 19.69 | | | 250.00 | | | 95.91 | | 427.60 | | 87.07 | | 775.40 | | 148.33 | | 2734.75 | | 3.06 | | 12149.53 | | 2.33 | |  |
| JF-43-R-1-30 | 0.52 | | 5.51 | | 0.24 | | 1.82 | | 2.39 | | | 0.10 | | | 15.49 | | | 7.02 | | | 103.36 | | | 45.52 | | 228.01 | | 51.54 | | 509.78 | | 104.63 | | 1373.14 | | 2.80 | | 12779.35 | | 3.45 | |  |
| JF-43-R-1-31 | 0.15 | | 8.35 | | 0.44 | | 6.99 | | 16.11 | | | 0.71 | | | 98.55 | | | 37.59 | | | 461.70 | | | 172.37 | | 742.71 | | 143.98 | | 1257.52 | | 224.04 | | 2260.04 | | 1.88 | | 10366.91 | | 1.13 | |  |
| JF-43-R-1-32 | 0.00 | | 12.97 | | 0.14 | | 2.38 | | 5.55 | | | 1.67 | | | 32.71 | | | 11.97 | | | 142.21 | | | 53.14 | | 234.46 | | 48.03 | | 442.49 | | 86.36 | | 1533.96 | | 1.78 | | 10683.29 | | 0.78 | |  |
| JF-43-R-1-33 | 0.00 | | 9.26 | | 0.07 | | 1.54 | | 4.80 | | | 1.02 | | | 31.37 | | | 13.10 | | | 173.55 | | | 68.84 | | 322.29 | | 69.38 | | 654.77 | | 127.53 | | 2013.37 | | 2.19 | | 11213.74 | | 1.49 | |  |
| JF-43-R-1-34 | 0.12 | | 12.50 | | 0.27 | | 5.45 | | 9.66 | | | 1.89 | | | 65.98 | | | 22.85 | | | 279.24 | | | 103.02 | | 451.76 | | 89.46 | | 793.80 | | 150.82 | | 2944.99 | | 2.65 | | 10748.99 | | 1.60 | |  |
| JF-43-R-1-35 | 0.00 | | 7.64 | | 0.04 | | 1.02 | | 2.16 | | | 0.51 | | | 11.93 | | | 4.02 | | | 48.77 | | | 20.83 | | 99.28 | | 22.01 | | 225.00 | | 48.00 | | 625.96 | | 0.39 | | 10006.63 | | 0.13 | |  |
| JF-43-R-1-36 | 0.10 | | 11.99 | | 0.66 | | 12.26 | | 21.73 | | | 2.57 | | | 110.61 | | | 33.87 | | | 367.63 | | | 126.91 | | 520.80 | | 98.70 | | 861.44 | | 161.10 | | 3481.13 | | 2.87 | | 8372.26 | | 1.07 | |  |
| JF-43-R-1-37 | 20.97 | | 299.83 | | 2.99 | | 12.26 | | 7.11 | | | 1.61 | | | 22.99 | | | 7.15 | | | 75.04 | | | 26.95 | | 121.17 | | 25.68 | | 237.91 | | 47.94 | | 838.46 | | 3.33 | | 10114.79 | | 1.18 | |  |
| JF-43-R-1-38 | 0.14 | | 2.12 | | 0.22 | | 2.03 | | 3.83 | | | 0.41 | | | 24.53 | | | 10.67 | | | 137.75 | | | 53.82 | | 264.63 | | 61.39 | | 633.71 | | 138.43 | | 1673.43 | | 3.86 | | 12455.91 | | 2.83 | |  |
| JF-43-R-1-39 | 197.99 | | 493.83 | | 59.36 | | 282.33 | | 58.98 | | | 1.75 | | | 79.17 | | | 16.08 | | | 149.99 | | | 49.13 | | 198.15 | | 37.80 | | 342.64 | | 66.65 | | 1364.18 | | 5.61 | | 8739.94 | | 1.80 | |  |
| JF-43-R-1-40 | 2.96 | | 16.98 | | 1.34 | | 6.84 | | 5.47 | | | 0.29 | | | 27.75 | | | 10.97 | | | 147.52 | | | 59.35 | | 283.24 | | 59.92 | | 561.92 | | 111.17 | | 1753.11 | | 2.96 | | 11497.86 | | 2.42 | |  |
| JF-43-R-1-41 | 0.26 | | 4.47 | | 0.09 | | 1.42 | | 3.22 | | | 0.37 | | | 21.09 | | | 7.66 | | | 96.14 | | | 37.23 | | 175.03 | | 38.09 | | 362.57 | | 73.22 | | 1096.29 | | 2.03 | | 10786.11 | | 1.68 | |  |
| JF-43-R-1-42 | 0.14 | | 9.09 | | 0.26 | | 2.01 | | 2.36 | | | 0.51 | | | 11.35 | | | 3.55 | | | 38.49 | | | 14.43 | | 60.66 | | 12.48 | | 112.26 | | 21.73 | | 422.33 | | 0.60 | | 9134.91 | | 0.32 | |  |
| JF-43-R-1-43 | 0.26 | | 16.36 | | 0.49 | | 5.35 | | 8.65 | | | 0.91 | | | 37.06 | | | 11.39 | | | 126.63 | | | 43.43 | | 180.25 | | 35.03 | | 310.14 | | 60.80 | | 1222.90 | | 2.71 | | 4811.66 | | 1.14 | |  |
| JF-43-R-1-44 | 5.11 | | 96.61 | | 8.40 | | 53.56 | | 38.29 | | | 12.33 | | | 65.93 | | | 17.25 | | | 145.69 | | | 39.88 | | 154.47 | | 32.21 | | 304.49 | | 58.68 | | 1125.38 | | 3.91 | | 10645.09 | | 2.70 | |  |
| JF-43-R-1-45 | 0.29 | | 25.11 | | 0.25 | | 2.04 | | 2.89 | | | 0.86 | | | 11.57 | | | 3.98 | | | 48.84 | | | 19.56 | | 95.46 | | 21.91 | | 226.78 | | 49.03 | | 617.21 | | 1.29 | | 9634.26 | | 0.69 | |  |
| JF-43-R-1-46 | 29.51 | | 80.51 | | 7.01 | | 29.98 | | 6.47 | | | 1.23 | | | 16.51 | | | 4.69 | | | 56.79 | | | 22.17 | | 105.96 | | 24.22 | | 234.31 | | 50.86 | | 692.92 | | 1.17 | | 9506.67 | | 0.58 | |  |
| JF-43-R-1-47 | 6.24 | | 26.64 | | 4.18 | | 21.10 | | 16.47 | | | 2.11 | | | 64.37 | | | 32.70 | | | 465.55 | | | 182.32 | | 897.07 | | 212.83 | | 2108.25 | | 411.68 | | 4278.12 | | 5.92 | | 15252.59 | | 5.07 | |  |
| JF-43-R-1-48 | 0.49 | | 13.96 | | 0.56 | | 4.42 | | 4.72 | | | 0.82 | | | 19.33 | | | 6.33 | | | 74.90 | | | 27.93 | | 123.80 | | 26.37 | | 244.63 | | 50.72 | | 811.09 | | 2.65 | | 8321.09 | | 0.92 | |  |
| JF-43-R-1-49 | 0.33 | | 14.60 | | 0.11 | | 2.00 | | 3.96 | | | 0.52 | | | 21.11 | | | 7.39 | | | 99.09 | | | 39.87 | | 186.63 | | 40.73 | | 380.06 | | 76.95 | | 1189.92 | | 2.92 | | 7204.89 | | 0.93 | |  |
| JF-43-R-1-50 | 8.07 | | 38.37 | | 6.28 | | 31.18 | | 29.46 | | | 2.91 | | | 107.30 | | | 47.56 | | | 629.01 | | | 243.71 | | 1142.02 | | 250.48 | | 2345.69 | | 451.98 | | 2717.02 | | 13.38 | | 12051.67 | | 6.60 | |  |
| JF-43-R-1-51 | 61.84 | | 212.00 | | 31.21 | | 159.87 | | 37.25 | | | 1.24 | | | 26.88 | | | 4.54 | | | 39.17 | | | 13.69 | | 61.53 | | 13.75 | | 134.07 | | 30.82 | | 419.48 | | 0.68 | | 9052.31 | | 0.24 | |  |
| JF-43-R-1-52 | 0.03 | | 7.22 | | 0.02 | | 0.57 | | 0.57 | | | 0.20 | | | 2.99 | | | 0.86 | | | 10.36 | | | 4.11 | | 20.72 | | 5.07 | | 52.49 | | 12.33 | | 134.83 | | 0.47 | | 10527.33 | | 0.33 | |  |
| JF-43-R-1-53 | 0.04 | | 1.08 | | 0.11 | | 1.82 | | 3.89 | | | 0.57 | | | 29.70 | | | 13.14 | | | 151.86 | | | 56.69 | | 252.34 | | 51.07 | | 452.01 | | 89.46 | | 1635.26 | | 0.53 | | 10799.32 | | 0.29 | |  |
| JF-43-R-1-54 | 0.29 | | 3.19 | | 0.23 | | 1.69 | | 3.37 | | | 0.27 | | | 28.22 | | | 15.64 | | | 244.76 | | | 105.50 | | 534.33 | | 127.31 | | 1254.27 | | 255.79 | | 3069.91 | | 3.27 | | 14992.56 | | 3.20 | |  |
| JF-43-R-1-55 | 0.07 | | 4.95 | | 0.05 | | 0.86 | | 1.06 | | | 0.46 | | | 12.14 | | | 6.91 | | | 115.00 | | | 50.19 | | 221.57 | | 41.48 | | 323.77 | | 59.38 | | 1479.39 | | 5.14 | | 11995.37 | | 2.99 | |  |
| JF-43-R-1-56 | 0.15 | | 58.25 | | 0.24 | | 3.51 | | 7.09 | | | 1.91 | | | 32.65 | | | 10.23 | | | 113.23 | | | 41.35 | | 179.26 | | 37.92 | | 344.58 | | 68.67 | | 1228.97 | | 3.84 | | 9404.53 | | 1.29 | |  |
| JF-43-R-1-57 | 0.14 | | 4.66 | | 0.15 | | 1.81 | | 3.52 | | | 0.44 | | | 31.54 | | | 12.12 | | | 156.75 | | | 61.72 | | 282.29 | | 58.62 | | 528.69 | | 106.51 | | 1789.70 | | 2.62 | | 11466.07 | | 1.13 | |  |
| JF-43-R-1-58 | 0.08 | | 1.95 | | 0.22 | | 2.05 | | 4.67 | | | 0.33 | | | 21.88 | | | 6.70 | | | 63.13 | | | 19.63 | | 71.45 | | 12.92 | | 107.32 | | 19.02 | | 595.08 | | 0.92 | | 12128.62 | | 0.82 | |  |
| JF-43-R-1-59 | 0.81 | | 21.97 | | 0.46 | | 3.87 | | 6.52 | | | 0.64 | | | 37.78 | | | 13.93 | | | 168.64 | | | 67.77 | | 303.12 | | 63.29 | | 554.55 | | 109.23 | | 1906.74 | | 4.70 | | 10599.34 | | 2.83 | |  |
| JF-43-R-1-60 | 0.01 | | 7.35 | | 0.12 | | 2.57 | | 6.60 | | | 0.67 | | | 36.53 | | | 12.98 | | | 163.71 | | | 64.39 | | 296.89 | | 61.13 | | 548.09 | | 113.49 | | 1865.31 | | 2.89 | | 8955.78 | | 1.13 | |  |
| JF-43-R-1-61 | 0.28 | | 9.42 | | 0.50 | | 4.31 | | 6.23 | | | 2.29 | | | 29.85 | | | 8.87 | | | 98.99 | | | 37.11 | | 160.52 | | 33.35 | | 316.54 | | 65.02 | | 1053.35 | | 2.14 | | 9714.80 | | 0.88 | |  |
| JF-43-R-1-62 | 0.02 | | 13.12 | | 0.02 | | 0.50 | | 1.41 | | | 0.29 | | | 6.05 | | | 1.80 | | | 19.86 | | | 7.39 | | 33.27 | | 7.38 | | 70.34 | | 14.69 | | 236.57 | | 0.59 | | 9300.19 | | 0.30 | |  |
| JF-43-R-1-63 | 0.00 | | 3.38 | | 0.05 | | 1.26 | | 5.62 | | | 0.14 | | | 55.01 | | | 29.09 | | | 422.33 | | | 181.38 | | 886.64 | | 191.95 | | 1767.35 | | 343.66 | | 4239.58 | | 4.35 | | 13643.16 | | 3.55 | |  |
| JF-43-R-1-64 | 0.50 | | 9.82 | | 0.43 | | 3.07 | | 5.26 | | | 0.51 | | | 34.62 | | | 14.51 | | | 195.42 | | | 80.02 | | 376.80 | | 79.78 | | 730.33 | | 143.64 | | 2339.34 | | 3.51 | | 11793.37 | | 3.23 | |  |
| JF-43-R-1-65 | 0.12 | | 10.73 | | 0.12 | | 1.97 | | 2.74 | | | 0.97 | | | 12.27 | | | 4.03 | | | 42.11 | | | 13.79 | | 61.77 | | 12.00 | | 113.45 | | 23.27 | | 428.73 | | 1.37 | | 9428.88 | | 0.77 | |  |
| JF-43-R-1-66 | 0.01 | | 7.63 | | 0.05 | | 1.36 | | 3.70 | | | 0.37 | | | 24.70 | | | 9.53 | | | 121.42 | | | 48.19 | | 217.33 | | 45.60 | | 407.56 | | 81.59 | | 1390.75 | | 1.44 | | 10426.96 | | 0.84 | |  |
| JF-43-R-1-67 | 0.05 | | 9.20 | | 0.06 | | 1.45 | | 4.14 | | | 0.26 | | | 27.05 | | | 10.23 | | | 128.69 | | | 50.21 | | 230.66 | | 48.63 | | 441.30 | | 86.15 | | 1484.72 | | 1.94 | | 10803.60 | | 1.35 | |  |
| JF-43-R-1-68 | 0.64 | | 6.35 | | 0.79 | | 6.08 | | 8.78 | | | 0.52 | | | 48.34 | | | 16.33 | | | 179.61 | | | 60.72 | | 246.59 | | 46.92 | | 393.39 | | 74.06 | | 1784.14 | | 0.69 | | 11030.85 | | 0.45 | |  |
| JF-43-R-1-69 | 1.67 | | 66.52 | | 3.00 | | 20.86 | | 23.89 | | | 5.90 | | | 46.53 | | | 15.65 | | | 155.77 | | | 48.79 | | 197.99 | | 38.78 | | 354.10 | | 66.86 | | 1419.31 | | 3.39 | | 9329.96 | | 1.16 | |  |
| JF-43-R-1-70 | 0.21 | | 21.57 | | 0.21 | | 1.72 | | 3.64 | | | 0.60 | | | 19.01 | | | 6.07 | | | 59.16 | | | 20.62 | | 94.61 | | 21.71 | | 225.77 | | 52.04 | | 633.94 | | 2.51 | | 10723.76 | | 0.77 | |  |
| JF-43-R-1-71 | 0.00 | | 13.93 | | 0.10 | | 2.31 | | 5.36 | | | 0.13 | | | 23.90 | | | 9.07 | | | 105.73 | | | 38.47 | | 169.71 | | 34.51 | | 303.03 | | 57.24 | | 1120.23 | | 2.83 | | 11634.98 | | 1.45 | |  |
| JF-43-R-1-72 | 0.94 | | 17.42 | | 0.24 | | 2.05 | | 1.80 | | | 1.00 | | | 11.20 | | | 3.97 | | | 46.59 | | | 19.36 | | 91.89 | | 21.29 | | 223.46 | | 52.03 | | 619.78 | | 1.57 | | 9170.22 | | 0.63 | |  |
| JF-43-R-1-73 | 0.01 | | 26.47 | | 0.07 | | 1.08 | | 2.19 | | | 0.31 | | | 10.47 | | | 3.18 | | | 32.15 | | | 11.20 | | 49.41 | | 10.39 | | 102.06 | | 21.07 | | 358.71 | | 1.48 | | 10745.88 | | 0.57 | |  |
| JF-43-R-1-74 | 0.03 | | 2.17 | | 0.07 | | 0.98 | | 1.54 | | | 0.23 | | | 11.75 | | | 4.42 | | | 60.04 | | | 26.68 | | 143.51 | | 36.63 | | 416.05 | | 96.85 | | 818.47 | | 2.03 | | 14920.85 | | 3.62 | |  |
| JF-43-R-1-75 | 0.09 | | 8.71 | | 0.35 | | 6.29 | | 13.04 | | | 0.73 | | | 76.93 | | | 27.92 | | | 353.34 | | | 134.42 | | 590.65 | | 119.31 | | 1028.92 | | 198.11 | | 3623.40 | | 1.77 | | 11275.16 | | 1.31 | |  |
| JF-43-R-1-76 | 0.02 | | 4.58 | | 0.08 | | 1.33 | | 2.26 | | | 0.19 | | | 15.61 | | | 5.72 | | | 74.59 | | | 29.70 | | 135.82 | | 29.06 | | 265.44 | | 54.85 | | 881.10 | | 1.83 | | 9874.75 | | 0.97 | |  |
| JF-43-R-1-77 | 1.75 | | 16.75 | | 1.83 | | 13.33 | | 10.25 | | | 2.68 | | | 38.09 | | | 10.33 | | | 107.41 | | | 36.01 | | 145.57 | | 28.00 | | 233.76 | | 44.05 | | 991.58 | | 7.94 | | 9077.73 | | 2.91 | |  |
| JF-43-R-1-78 | 85.31 | | 161.98 | | 13.64 | | 61.87 | | 6.63 | | | 1.22 | | | 17.54 | | | 5.88 | | | 77.46 | | | 31.82 | | 163.89 | | 38.14 | | 395.98 | | 89.60 | | 1018.20 | | 1.49 | | 10407.89 | | 0.57 | |  |
| JF-43-R-1-79 | 0.04 | | 2.17 | | 0.14 | | 2.50 | | 5.58 | | | 0.25 | | | 38.41 | | | 16.90 | | | 236.59 | | | 98.04 | | 455.19 | | 94.95 | | 854.43 | | 166.88 | | 2798.62 | | 1.35 | | 11890.65 | | 0.96 | |  |
| JF-43-R-1-80 | 0.00 | | 10.08 | | 0.03 | | 0.72 | | 0.85 | | | 0.14 | | | 5.30 | | | 1.86 | | | 22.23 | | | 9.73 | | 54.38 | | 15.38 | | 176.96 | | 42.60 | | 329.25 | | 1.65 | | 11482.17 | | 0.67 | |  |
| JF-43-R-1-81 | 1.90 | | 10.52 | | 0.84 | | 6.29 | | 8.16 | | | 0.77 | | | 48.24 | | | 21.52 | | | 263.72 | | | 108.21 | | 496.21 | | 103.96 | | 937.21 | | 182.79 | | 3137.34 | | 3.83 | | 11310.54 | | 2.30 | |  |
| JF-43-R-1-82 | 0.09 | | 3.81 | | 0.67 | | 6.47 | | 15.29 | | | 1.23 | | | 70.74 | | | 19.11 | | | 181.91 | | | 53.00 | | 197.03 | | 36.88 | | 333.71 | | 58.36 | | 1552.42 | | 0.80 | | 11314.36 | | 0.53 | |  |
| JF-43-R-1-83 | 0.07 | | 12.16 | | 0.60 | | 9.65 | | 18.36 | | | 1.67 | | | 99.51 | | | 35.35 | | | 408.18 | | | 159.27 | | 669.26 | | 130.23 | | 1132.50 | | 208.03 | | 4504.67 | | 2.89 | | 9944.13 | | 1.88 | |  |
| JF-43-R-1-84 | 0.81 | | 31.91 | | 0.68 | | 6.81 | | 10.92 | | | 2.01 | | | 54.92 | | | 18.78 | | | 214.72 | | | 81.36 | | 369.61 | | 78.09 | | 726.48 | | 146.38 | | 2366.98 | | 10.12 | | 9486.05 | | 4.37 | |  |
| JF-43-R-1-85 | 31.51 | | 197.73 | | 44.68 | | 284.08 | | 205.77 | | | 70.54 | | | 390.35 | | | 89.76 | | | 624.51 | | | 137.43 | | 431.06 | | 75.57 | | 620.63 | | 101.89 | | 1284.58 | | 1.31 | | 13283.00 | | 0.83 | |  |
| JF-43-R-1-86 | 0.09 | | 14.13 | | 0.30 | | 3.37 | | 3.95 | | | 2.27 | | | 15.97 | | | 4.80 | | | 48.54 | | | 16.45 | | 66.90 | | 13.58 | | 123.00 | | 24.08 | | 465.61 | | 0.74 | | 9977.75 | | 0.24 | |  |
| JF-43-R-1-87 | 0.11 | | 5.94 | | 0.32 | | 4.56 | | 8.16 | | | 0.55 | | | 42.20 | | | 15.63 | | | 178.69 | | | 67.05 | | 294.23 | | 58.03 | | 503.54 | | 97.10 | | 1917.01 | | 2.04 | | 9964.71 | | 1.40 | |  |
| JF-43-R-1-88 | 5.67 | | 34.78 | | 4.80 | | 27.69 | | 21.63 | | | 2.38 | | | 58.14 | | | 18.89 | | | 204.74 | | | 74.83 | | 327.72 | | 69.32 | | 639.78 | | 126.70 | | 2155.12 | | 8.87 | | 12904.49 | | 9.02 | |  |
| JF-43-R-1-89 | 0.04 | | 25.10 | | 0.15 | | 1.95 | | 4.60 | | | 0.89 | | | 28.35 | | | 9.89 | | | 130.78 | | | 52.70 | | 240.80 | | 48.97 | | 448.99 | | 89.61 | | 1442.11 | | 4.20 | | 8737.97 | | 1.02 | |  |
| JF-43-R-1-90 | 0.03 | | 3.56 | | 0.08 | | 1.25 | | 7.35 | | | 0.19 | | | 64.55 | | | 31.66 | | | 476.69 | | | 199.80 | | 966.60 | | 208.09 | | 1909.66 | | 366.29 | | 5023.70 | | 3.91 | | 12634.65 | | 2.85 | |  |
| JF-43-R-1-91 | 0.02 | | 10.67 | | 0.02 | | 0.74 | | 1.80 | | | 0.01 | | | 11.28 | | | 4.64 | | | 63.96 | | | 26.96 | | 131.08 | | 28.92 | | 281.81 | | 61.22 | | 781.33 | | 10.01 | | 12499.27 | | 7.82 | |  |
| JF-43-R-1-92 | 14.51 | | 87.39 | | 8.96 | | 53.78 | | 30.31 | | | 8.39 | | | 65.57 | | | 17.09 | | | 179.00 | | | 61.32 | | 269.52 | | 57.26 | | 545.65 | | 106.65 | | 1786.20 | | 7.22 | | 9750.40 | | 2.98 | |  |
| JF-43-R-1-93 | 74.30 | | 205.05 | | 27.43 | | 139.06 | | 34.74 | | | 2.29 | | | 61.01 | | | 14.40 | | | 152.89 | | | 55.82 | | 244.89 | | 49.90 | | 446.57 | | 89.30 | | 1554.97 | | 3.07 | | 9294.15 | | 1.02 | |  |
| JF-43-R-1-94 | 0.01 | | 8.83 | | 0.00 | | 0.70 | | 1.38 | | | 0.51 | | | 7.90 | | | 2.84 | | | 35.21 | | | 13.91 | | 66.75 | | 15.64 | | 154.14 | | 34.43 | | 429.50 | | 1.11 | | 8797.51 | | 0.37 | |  |
| JF-43-R-1-95 | 0.10 | | 43.42 | | 0.14 | | 1.26 | | 2.34 | | | 0.34 | | | 7.57 | | | 2.83 | | | 32.43 | | | 12.35 | | 55.65 | | 12.63 | | 128.29 | | 28.73 | | 380.50 | | 2.45 | | 10329.03 | | 1.77 | |  |
| JF-43-R-1-96 | 0.03 | | 20.14 | | 0.04 | | 0.64 | | 1.58 | | | 0.32 | | | 8.57 | | | 2.96 | | | 35.61 | | | 14.19 | | 63.80 | | 14.25 | | 137.52 | | 28.04 | | 429.16 | | 1.35 | | 10398.88 | | 0.71 | |  |
| JF-43-R-1-97 | 0.13 | | 4.54 | | 0.13 | | 1.33 | | 3.48 | | | 0.04 | | | 19.47 | | | 7.24 | | | 91.42 | | | 35.45 | | 161.56 | | 32.78 | | 305.31 | | 59.60 | | 1038.41 | | 2.61 | | 10677.92 | | 1.31 | |  |
| JF-43-R-1-98 | 0.00 | | 2.54 | | 0.05 | | 0.71 | | 1.83 | | | 0.05 | | | 12.84 | | | 4.80 | | | 59.01 | | | 23.39 | | 106.47 | | 22.82 | | 205.24 | | 40.79 | | 686.83 | | 1.13 | | 10642.45 | | 0.72 | |  |
| JF-43-R-1-99 | 0.30 | | 7.88 | | 0.77 | | 4.89 | | 3.42 | | | 1.15 | | | 9.13 | | | 2.53 | | | 26.46 | | | 8.91 | | 40.83 | | 9.14 | | 93.32 | | 19.88 | | 274.79 | | 2.01 | | 9265.65 | | 1.50 | |  |
| JF-43-R-1-100 | 5.70 | | 27.53 | | 2.90 | | 16.81 | | 6.22 | | | 2.75 | | | 14.93 | | | 5.11 | | | 63.87 | | | 24.27 | | 111.38 | | 23.60 | | 226.61 | | 43.87 | | 794.68 | | 2.39 | | 11842.11 | | 2.57 | |  |
| JF-43-R-1-101 | 41.06 | | 279.23 | | 47.29 | | 276.60 | | 166.88 | | | 52.83 | | | 266.08 | | | 76.55 | | | 725.70 | | | 197.30 | | 723.56 | | 135.27 | | 1153.25 | | 197.59 | | 4691.83 | | 27.65 | | 10292.23 | | 9.91 | |  |
| JF-43-R-1-102 | 0.07 | | 7.08 | | 0.39 | | 6.61 | | 11.77 | | | 1.39 | | | 61.40 | | | 18.55 | | | 206.90 | | | 74.46 | | 318.84 | | 68.12 | | 679.42 | | 142.71 | | 2058.89 | | 2.86 | | 11853.71 | | 2.78 | |  |
| JF-43-R-1-103 | 0.73 | | 5.48 | | 0.80 | | 4.41 | | 6.73 | | | 0.21 | | | 46.18 | | | 25.50 | | | 382.38 | | | 151.56 | | 752.43 | | 187.52 | | 1940.70 | | 389.39 | | 4238.77 | | 5.29 | | 17213.83 | | 4.95 | |  |
| JF-43-R-1-104 | 0.01 | | 2.63 | | 0.03 | | 0.85 | | 4.00 | | | 0.09 | | | 35.40 | | | 17.78 | | | 270.29 | | | 115.93 | | 569.06 | | 126.98 | | 1213.17 | | 240.71 | | 3420.41 | | 3.38 | | 13314.09 | | 2.73 | |  |
| JF-43-R-1-105 | 43.69 | | 258.59 | | 40.78 | | 286.76 | | 147.52 | | | 65.84 | | | 264.10 | | | 62.20 | | | 524.11 | | | 140.07 | | 519.91 | | 102.41 | | 910.12 | | 165.94 | | 3924.92 | | 10.56 | | 10307.85 | | 6.53 | |  |
| JF-43-R-1-106 | 0.04 | | 5.16 | | 0.03 | | 0.59 | | 0.69 | | | 0.21 | | | 3.63 | | | 1.11 | | | 16.27 | | | 7.30 | | 38.99 | | 10.59 | | 129.14 | | 33.42 | | 234.66 | | 0.74 | | 10275.16 | | 0.30 | |  |
| JF-43-R-1-107 | 0.02 | | 5.41 | | 0.07 | | 1.53 | | 3.02 | | | 0.22 | | | 18.18 | | | 5.76 | | | 68.43 | | | 25.63 | | 108.69 | | 22.08 | | 199.21 | | 39.29 | | 747.65 | | 0.98 | | 9267.47 | | 0.43 | |  |
| JF-43-R-1-108 | 0.01 | | 1.17 | | 0.03 | | 0.36 | | 1.78 | | | 0.05 | | | 19.71 | | | 12.81 | | | 198.86 | | | 83.69 | | 440.88 | | 115.00 | | 1254.99 | | 265.81 | | 2461.65 | | 3.36 | | 17964.93 | | 3.83 | |  |
| JF-43-R-1-109 | 17.61 | | 92.02 | | 18.30 | | 116.89 | | 82.98 | | | 22.70 | | | 154.49 | | | 43.62 | | | 435.23 | | | 141.60 | | 617.91 | | 131.72 | | 1251.09 | | 250.09 | | 4269.86 | | 3.22 | | 12621.56 | | 2.14 | |  |
| JF-43-R-1-110 | 529.02 | | 2364.39 | | 427.08 | | 2200.81 | | 516.54 | | | 146.69 | | | 389.44 | | | 50.96 | | | 263.25 | | | 46.51 | | 113.86 | | 14.76 | | 97.04 | | 12.28 | | 1191.09 | | 372.15 | | 18.31 | | 25.89 | |  |

Supplementary Table 3. Detrital zircon Lu-Hf isotopic compositions of the sedimentary rocks from the Early-Middle Triassic in the study region.

| Site number | | T (Ma) | 176Yb/177Hf | | 176Lu/177Hf | | ±2σ | | 176Hf/177Hf | | ±2σ | | εHf(*t*) | | ±2σ | | TDM (Ma) | | ±2σ | | TCDM (Ma) | | ±2σ | |
| --- | --- | --- | --- | --- | --- | --- | --- | --- | --- | --- | --- | --- | --- | --- | --- | --- | --- | --- | --- | --- | --- | --- | --- | --- |
| QJP-39-R-1 | |  |  | |  | |  | |  | |  | |  | |  | |  | |  | |  | |  | |
| QJP-39-R-1-03 | 2458.9 | | | 0.018837 | | 0.000698 | | 0.000007 | | 0.281125 | | 0.000028 | | -4.3 | | 1.0 | | 2940 | | 38 | | 3227 | | 61 |
| QJP-39-R-1-08 | 760.3 | | | 0.054302 | | 0.002120 | | 0.000018 | | 0.281895 | | 0.000032 | | -15.3 | | 1.1 | | 1966 | | 46 | | 2615 | | 70 |
| QJP-39-R-1-15 | 1944.5 | | | 0.038461 | | 0.001377 | | 0.000009 | | 0.281405 | | 0.000032 | | -6.8 | | 1.2 | | 2608 | | 45 | | 2986 | | 70 |
| QJP-39-R-1-21 | 331.0 | | | 0.031925 | | 0.001256 | | 0.000003 | | 0.282523 | | 0.000019 | | -1.8 | | 0.7 | | 1040 | | 27 | | 1450 | | 42 |
| QJP-39-R-1-24 | 2514.5 | | | 0.024941 | | 0.000990 | | 0.000007 | | 0.281258 | | 0.000027 | | 1.2 | | 0.9 | | 2782 | | 36 | | 2936 | | 58 |
| QJP-39-R-1-27 | 2620.4 | | | 0.036713 | | 0.001422 | | 0.000002 | | 0.281365 | | 0.000020 | | 6.6 | | 0.7 | | 2666 | | 28 | | 2688 | | 44 |
| QJP-39-R-1-36 | 2552.8 | | | 0.022540 | | 0.000872 | | 0.000007 | | 0.281263 | | 0.000024 | | 2.4 | | 0.8 | | 2767 | | 32 | | 2891 | | 51 |
| QJP-39-R-1-38 | 758.3 | | | 0.060307 | | 0.002193 | | 0.000007 | | 0.282395 | | 0.000029 | | 2.3 | | 1.0 | | 1251 | | 42 | | 1517 | | 64 |
| QJP-39-R-1-42 | 2450.3 | | | 0.038962 | | 0.001439 | | 0.000018 | | 0.281301 | | 0.000023 | | 0.6 | | 0.8 | | 2755 | | 32 | | 2926 | | 51 |
| QJP-39-R-1-49 | 2491.1 | | | 0.023568 | | 0.000916 | | 0.000003 | | 0.281250 | | 0.000029 | | 0.5 | | 1.0 | | 2787 | | 39 | | 2959 | | 62 |
| QJP-39-R-1-50 | 2498.2 | | | 0.013519 | | 0.000571 | | 0.000004 | | 0.281303 | | 0.000033 | | 3.1 | | 1.2 | | 2692 | | 45 | | 2806 | | 72 |
| QJP-39-R-1-64 | 2553.7 | | | 0.006022 | | 0.000276 | | 0.000001 | | 0.281332 | | 0.000018 | | 6.0 | | 0.7 | | 2632 | | 24 | | 2677 | | 40 |
| QJP-39-R-1-70 | 814.7 | | | 0.024542 | | 0.000949 | | 0.000005 | | 0.281960 | | 0.000027 | | -11.3 | | 0.9 | | 1816 | | 37 | | 2405 | | 58 |
| QJP-39-R-1-72 | 2520.4 | | | 0.012433 | | 0.000529 | | 0.000006 | | 0.281253 | | 0.000032 | | 2.0 | | 1.2 | | 2755 | | 44 | | 2894 | | 70 |
| QJP-39-R-1-76 | 307.8 | | | 0.024295 | | 0.000803 | | 0.000004 | | 0.282280 | | 0.000020 | | -10.8 | | 0.7 | | 1366 | | 28 | | 1995 | | 45 |
| QJP-39-R-1-80 | 2413.3 | | | 0.015501 | | 0.000592 | | 0.000005 | | 0.281354 | | 0.000026 | | 3.0 | | 0.9 | | 2624 | | 36 | | 2749 | | 57 |
| QJP-39-R-1-83 | 2462.0 | | | 0.012387 | | 0.000539 | | 0.000005 | | 0.281490 | | 0.000029 | | 9.0 | | 1.0 | | 2436 | | 39 | | 2416 | | 64 |
| QJP-39-R-1-85 | 438.8 | | | 0.016497 | | 0.000697 | | 0.000009 | | 0.282634 | | 0.000020 | | 4.6 | | 0.7 | | 869 | | 28 | | 1129 | | 45 |
| QJP-39-R-1-88 | 2487.0 | | | 0.010695 | | 0.000451 | | 0.000001 | | 0.281291 | | 0.000018 | | 2.7 | | 0.6 | | 2699 | | 25 | | 2826 | | 40 |
| QJP-39-R-1-97 | 786.8 | | | 0.045777 | | 0.001741 | | 0.000004 | | 0.282094 | | 0.000025 | | -7.5 | | 0.9 | | 1665 | | 36 | | 2153 | | 55 |
| QJP-39-R-1-99 | 2438.9 | | | 0.012033 | | 0.000524 | | 0.000004 | | 0.281379 | | 0.000026 | | 4.6 | | 0.9 | | 2585 | | 35 | | 2670 | | 56 |
| QJP-39-R-1-107 | 2683.3 | | | 0.023764 | | 0.000961 | | 0.000003 | | 0.281271 | | 0.000027 | | 5.5 | | 0.9 | | 2762 | | 36 | | 2805 | | 58 |
| QJP-39-R-1-108 | 2483.6 | | | 0.016852 | | 0.000659 | | 0.000006 | | 0.281276 | | 0.000022 | | 1.7 | | 0.8 | | 2734 | | 29 | | 2882 | | 47 |
| QJP-39-R-1-113 | 1791.7 | | | 0.033763 | | 0.001216 | | 0.000013 | | 0.281553 | | 0.000029 | | -4.7 | | 1.0 | | 2393 | | 41 | | 2740 | | 64 |
| QJP-39-R-1-114 | 2626.9 | | | 0.027800 | | 0.000954 | | 0.000012 | | 0.281094 | | 0.000020 | | -2.0 | | 0.7 | | 3002 | | 27 | | 3220 | | 43 |
| QJP-39-R-1-124 | 2546.0 | | | 0.009698 | | 0.000392 | | 0.000003 | | 0.281311 | | 0.000020 | | 4.8 | | 0.7 | | 2668 | | 27 | | 2740 | | 44 |
| QJP-39-R-1-131 | 2475.6 | | | 0.039331 | | 0.001465 | | 0.000025 | | 0.281301 | | 0.000023 | | 1.1 | | 0.8 | | 2757 | | 32 | | 2914 | | 50 |
| QJP-39-R-1-132 | 2501.9 | | | 0.001635 | | 0.000069 | | 0.000001 | | 0.281304 | | 0.000017 | | 4.2 | | 0.6 | | 2655 | | 23 | | 2747 | | 38 |
| QJP-39-R-1-133 | 305.3 | | | 0.038518 | | 0.001560 | | 0.000014 | | 0.282668 | | 0.000025 | | 2.7 | | 0.9 | | 841 | | 36 | | 1145 | | 56 |
| QJP-39-R-1-135 | 2479.6 | | | 0.051231 | | 0.002140 | | 0.000015 | | 0.281377 | | 0.000030 | | 2.7 | | 1.1 | | 2701 | | 43 | | 2818 | | 66 |
| QJP-39-R-1-139 | 750.2 | | | 0.064237 | | 0.002210 | | 0.000046 | | 0.282286 | | 0.000027 | | -1.7 | | 1.0 | | 1409 | | 39 | | 1764 | | 59 |
| SBY-2-R-1 | |  |  | |  | |  | |  | |  | |  | |  | |  | |  | |  | |  | |
| SBY-2-R-1-01 | 836.1 | | | 0.028614 | | 0.001123 | | 0.000003 | | 0.281699 | | 0.000018 | | -20.1 | | 0.6 | | 2186 | | 24 | | 2967 | | 38 |
| SBY-2-R-1-03 | 273.3 | | | 0.027224 | | 0.001099 | | 0.000005 | | 0.282811 | | 0.000019 | | 7.2 | | 0.7 | | 627 | | 27 | | 835 | | 44 |
| SBY-2-R-1-07 | 1023.9 | | | 0.020502 | | 0.000716 | | 0.000006 | | 0.281984 | | 0.000016 | | -5.7 | | 0.6 | | 1772 | | 22 | | 2221 | | 36 |
| SBY-2-R-1-08 | 450.3 | | | 0.030543 | | 0.001199 | | 0.000011 | | 0.282381 | | 0.000018 | | -4.3 | | 0.6 | | 1238 | | 25 | | 1696 | | 40 |
| SBY-2-R-1-09 | 1706.5 | | | 0.014922 | | 0.000609 | | 0.000020 | | 0.281621 | | 0.000019 | | -3.4 | | 0.7 | | 2262 | | 26 | | 2597 | | 42 |
| SBY-2-R-1-10 | 1022.6 | | | 0.031864 | | 0.001150 | | 0.000003 | | 0.282187 | | 0.000018 | | 1.1 | | 0.6 | | 1509 | | 25 | | 1793 | | 39 |
| SBY-2-R-1-14 | 2516.4 | | | 0.022651 | | 0.000803 | | 0.000014 | | 0.281202 | | 0.000019 | | -0.4 | | 0.7 | | 2844 | | 25 | | 3037 | | 40 |
| SBY-2-R-1-15 | 433.2 | | | 0.063931 | | 0.002408 | | 0.000007 | | 0.282395 | | 0.000026 | | -4.5 | | 0.9 | | 1259 | | 38 | | 1696 | | 58 |
| SBY-2-R-1-16 | 1001.3 | | | 0.017219 | | 0.000673 | | 0.000001 | | 0.282209 | | 0.000019 | | 1.8 | | 0.7 | | 1460 | | 27 | | 1736 | | 43 |
| SBY-2-R-1-17 | 1031.5 | | | 0.051607 | | 0.001959 | | 0.000007 | | 0.282367 | | 0.000018 | | 7.2 | | 0.6 | | 1283 | | 26 | | 1421 | | 40 |
| SBY-2-R-1-19 | 986.3 | | | 0.021886 | | 0.000788 | | 0.000001 | | 0.281880 | | 0.000018 | | -10.3 | | 0.6 | | 1918 | | 24 | | 2473 | | 38 |
| SBY-2-R-1-21 | 923.6 | | | 0.019469 | | 0.000709 | | 0.000001 | | 0.282190 | | 0.000023 | | -0.6 | | 0.8 | | 1488 | | 32 | | 1827 | | 51 |
| SBY-2-R-1-25 | 410.9 | | | 0.030664 | | 0.001117 | | 0.000000 | | 0.282395 | | 0.000016 | | -4.6 | | 0.6 | | 1215 | | 23 | | 1685 | | 37 |
| SBY-2-R-1-30 | 410.4 | | | 0.028370 | | 0.001092 | | 0.000020 | | 0.282354 | | 0.000020 | | -6.0 | | 0.7 | | 1272 | | 28 | | 1775 | | 45 |
| SBY-2-R-1-31 | 1533.3 | | | 0.020637 | | 0.000778 | | 0.000002 | | 0.281843 | | 0.000020 | | 0.5 | | 0.7 | | 1968 | | 27 | | 2227 | | 43 |
| SBY-2-R-1-32 | 877.0 | | | 0.030795 | | 0.001084 | | 0.000010 | | 0.282189 | | 0.000016 | | -1.9 | | 0.6 | | 1503 | | 22 | | 1870 | | 35 |
| SBY-2-R-1-35 | 1014.9 | | | 0.007576 | | 0.000281 | | 0.000002 | | 0.282194 | | 0.000021 | | 1.8 | | 0.7 | | 1465 | | 28 | | 1744 | | 46 |
| SBY-2-R-1-36 | 851.4 | | | 0.001461 | | 0.000051 | | 0.000002 | | 0.282025 | | 0.000015 | | -7.6 | | 0.5 | | 1686 | | 20 | | 2209 | | 33 |
| SBY-2-R-1-37 | 391.0 | | | 0.028377 | | 0.001158 | | 0.000012 | | 0.282351 | | 0.000018 | | -6.6 | | 0.6 | | 1279 | | 25 | | 1795 | | 40 |
| SBY-2-R-1-39 | 1061.1 | | | 0.058569 | | 0.002457 | | 0.000028 | | 0.281900 | | 0.000029 | | -9.1 | | 1.0 | | 1977 | | 41 | | 2458 | | 62 |
| SBY-2-R-1-40 | 1075.9 | | | 0.025442 | | 0.000930 | | 0.000001 | | 0.281965 | | 0.000020 | | -5.4 | | 0.7 | | 1808 | | 28 | | 2240 | | 45 |
| SBY-2-R-1-41 | 400.9 | | | 0.027667 | | 0.001048 | | 0.000003 | | 0.282288 | | 0.000025 | | -8.6 | | 0.9 | | 1363 | | 35 | | 1926 | | 56 |
| SBY-2-R-1-42 | 888.9 | | | 0.018777 | | 0.000689 | | 0.000001 | | 0.282124 | | 0.000022 | | -3.7 | | 0.8 | | 1578 | | 31 | | 1993 | | 49 |
| SBY-2-R-1-43 | 430.3 | | | 0.029988 | | 0.001203 | | 0.000011 | | 0.282239 | | 0.000022 | | -9.7 | | 0.8 | | 1438 | | 31 | | 2021 | | 49 |
| SBY-2-R-1-44 | 2492.3 | | | 0.018114 | | 0.000684 | | 0.000002 | | 0.281336 | | 0.000016 | | 4.0 | | 0.6 | | 2654 | | 22 | | 2749 | | 36 |
| SBY-2-R-1-45 | 1661.1 | | | 0.032176 | | 0.001269 | | 0.000006 | | 0.281832 | | 0.000020 | | 2.3 | | 0.7 | | 2010 | | 28 | | 2210 | | 45 |
| SBY-2-R-1-46 | 401.5 | | | 0.084486 | | 0.002867 | | 0.000009 | | 0.282366 | | 0.000021 | | -6.3 | | 0.7 | | 1317 | | 30 | | 1783 | | 46 |
| SBY-2-R-1-49 | 393.9 | | | 0.031380 | | 0.001258 | | 0.000002 | | 0.282424 | | 0.000019 | | -4.0 | | 0.7 | | 1180 | | 26 | | 1634 | | 42 |
| SBY-2-R-1-50 | 862.9 | | | 0.008135 | | 0.000260 | | 0.000005 | | 0.281991 | | 0.000019 | | -8.7 | | 0.7 | | 1741 | | 25 | | 2285 | | 41 |
| SBY-2-R-1-51 | 1094.4 | | | 0.028710 | | 0.000973 | | 0.000010 | | 0.281981 | | 0.000016 | | -4.5 | | 0.6 | | 1788 | | 23 | | 2197 | | 36 |
| SBY-2-R-1-52 | 975.9 | | | 0.028045 | | 0.001005 | | 0.000002 | | 0.282234 | | 0.000016 | | 1.9 | | 0.6 | | 1437 | | 22 | | 1709 | | 35 |
| SBY-2-R-1-53 | 250.7 | | | 0.031588 | | 0.001218 | | 0.000002 | | 0.282736 | | 0.000018 | | 4.0 | | 0.6 | | 736 | | 26 | | 1019 | | 41 |
| SBY-2-R-1-54 | 809.3 | | | 0.010871 | | 0.000337 | | 0.000010 | | 0.282242 | | 0.000015 | | -1.0 | | 0.5 | | 1401 | | 20 | | 1766 | | 33 |
| SBY-2-R-1-56 | 854.3 | | | 0.025348 | | 0.000933 | | 0.000001 | | 0.281965 | | 0.000016 | | -10.2 | | 0.6 | | 1809 | | 22 | | 2372 | | 34 |
| SBY-2-R-1-58 | 399.7 | | | 0.029957 | | 0.001203 | | 0.000012 | | 0.282218 | | 0.000019 | | -11.1 | | 0.7 | | 1468 | | 26 | | 2085 | | 41 |
| SBY-2-R-1-59 | 418.8 | | | 0.041349 | | 0.001554 | | 0.000003 | | 0.282406 | | 0.000018 | | -4.2 | | 0.6 | | 1214 | | 26 | | 1664 | | 40 |
| SBY-2-R-1-60 | 929.3 | | | 0.023598 | | 0.000837 | | 0.000006 | | 0.282295 | | 0.000018 | | 3.2 | | 0.6 | | 1346 | | 25 | | 1595 | | 41 |
| SBY-2-R-1-63 | 2599.1 | | | 0.015775 | | 0.000625 | | 0.000008 | | 0.280868 | | 0.000021 | | -10.1 | | 0.8 | | 3279 | | 29 | | 3687 | | 46 |
| SBY-2-R-1-65 | 854.2 | | | 0.014944 | | 0.000576 | | 0.000006 | | 0.282400 | | 0.000015 | | 5.4 | | 0.5 | | 1192 | | 21 | | 1396 | | 34 |
| SBY-2-R-1-66 | 410.4 | | | 0.038857 | | 0.001448 | | 0.000008 | | 0.282224 | | 0.000018 | | -10.7 | | 0.6 | | 1468 | | 26 | | 2068 | | 40 |
| SBY-2-R-1-67 | 269.1 | | | 0.023016 | | 0.001023 | | 0.000004 | | 0.282518 | | 0.000017 | | -3.3 | | 0.6 | | 1040 | | 24 | | 1493 | | 38 |
| SBY-2-R-1-68 | 255.7 | | | 0.023936 | | 0.001025 | | 0.000002 | | 0.282563 | | 0.000017 | | -2.0 | | 0.6 | | 977 | | 24 | | 1401 | | 38 |
| SBY-2-R-1-69 | 1720.4 | | | 0.019634 | | 0.000739 | | 0.000009 | | 0.281751 | | 0.000014 | | 1.4 | | 0.5 | | 2093 | | 20 | | 2316 | | 32 |
| SBY-2-R-1-71 | 952.8 | | | 0.037291 | | 0.001445 | | 0.000008 | | 0.282475 | | 0.000022 | | 9.7 | | 0.8 | | 1112 | | 32 | | 1203 | | 50 |
| SBY-2-R-1-74 | 938.4 | | | 0.018390 | | 0.000740 | | 0.000008 | | 0.281962 | | 0.000025 | | -8.4 | | 0.9 | | 1803 | | 35 | | 2319 | | 55 |
| SBY-2-R-1-75 | 387.9 | | | 0.025701 | | 0.001037 | | 0.000002 | | 0.282309 | | 0.000018 | | -8.1 | | 0.6 | | 1334 | | 26 | | 1888 | | 40 |
| SBY-2-R-1-80 | 429.6 | | | 0.034727 | | 0.001277 | | 0.000006 | | 0.282485 | | 0.000029 | | -1.0 | | 1.0 | | 1093 | | 42 | | 1477 | | 65 |
| SBY-2-R-1-84 | 2597.2 | | | 0.020885 | | 0.000819 | | 0.000004 | | 0.280874 | | 0.000030 | | -10.3 | | 1.1 | | 3287 | | 41 | | 3695 | | 65 |
| SBY-2-R-1-86 | 1164.8 | | | 0.008707 | | 0.000332 | | 0.000017 | | 0.281999 | | 0.000022 | | -1.8 | | 0.8 | | 1733 | | 29 | | 2084 | | 47 |
| JF-39-R-1 |  | | |  | |  | |  | |  | |  | |  | |  | |  | |  | |  | |  |
| JF-39-R-04 | 249.0 | | | 0.049940 | | 0.001900 | | 0.000021 | | 0.282338 | | 0.000023 | | -10.2 | | 0.8 | | 1323 | | 33 | | 1913 | | 50 |
| JF-39-R-05 | 247.6 | | | 0.074001 | | 0.002771 | | 0.000016 | | 0.282324 | | 0.000032 | | -10.9 | | 1.1 | | 1376 | | 46 | | 1955 | | 70 |
| JF-39-R-10 | 248.3 | | | 0.054007 | | 0.002025 | | 0.000008 | | 0.282384 | | 0.000025 | | -8.6 | | 0.9 | | 1261 | | 37 | | 1813 | | 56 |
| JF-39-R-11 | 280.8 | | | 0.047174 | | 0.001751 | | 0.000023 | | 0.282353 | | 0.000039 | | -9.0 | | 1.4 | | 1297 | | 56 | | 1862 | | 87 |
| JF-39-R-13 | 241.7 | | | 0.046908 | | 0.001793 | | 0.000005 | | 0.282384 | | 0.000028 | | -8.7 | | 1.0 | | 1254 | | 40 | | 1814 | | 62 |
| JF-39-R-15 | 248.3 | | | 0.088942 | | 0.003327 | | 0.000008 | | 0.282395 | | 0.000027 | | -8.4 | | 0.9 | | 1291 | | 40 | | 1801 | | 59 |
| JF-39-R-16 | 244.2 | | | 0.056398 | | 0.002147 | | 0.000014 | | 0.282366 | | 0.000028 | | -9.3 | | 1.0 | | 1292 | | 40 | | 1856 | | 61 |
| JF-39-R-19 | 252.7 | | | 0.042941 | | 0.001638 | | 0.000016 | | 0.282451 | | 0.000022 | | -6.1 | | 0.8 | | 1154 | | 31 | | 1659 | | 49 |
| JF-39-R-24 | 248.0 | | | 0.046787 | | 0.001727 | | 0.000018 | | 0.282405 | | 0.000027 | | -7.8 | | 0.9 | | 1221 | | 38 | | 1763 | | 59 |
| JF-39-R-28 | 249.9 | | | 0.053625 | | 0.002023 | | 0.000020 | | 0.282357 | | 0.000021 | | -9.5 | | 0.8 | | 1301 | | 31 | | 1873 | | 47 |
| JF-39-R-29 | 254.2 | | | 0.031682 | | 0.001215 | | 0.000002 | | 0.282392 | | 0.000021 | | -8.1 | | 0.8 | | 1224 | | 30 | | 1785 | | 47 |
| JF-39-R-32 | 240.3 | | | 0.041569 | | 0.001570 | | 0.000002 | | 0.282376 | | 0.000022 | | -9.0 | | 0.8 | | 1258 | | 32 | | 1831 | | 50 |
| JF-39-R-33 | 246.4 | | | 0.055802 | | 0.002108 | | 0.000005 | | 0.282382 | | 0.000023 | | -8.7 | | 0.8 | | 1267 | | 34 | | 1819 | | 52 |
| JF-39-R-34 | 243.9 | | | 0.035604 | | 0.001385 | | 0.000004 | | 0.282409 | | 0.000023 | | -7.7 | | 0.8 | | 1204 | | 32 | | 1753 | | 50 |
| JF-39-R-36 | 243.4 | | | 0.052937 | | 0.002022 | | 0.000003 | | 0.282358 | | 0.000023 | | -9.6 | | 0.8 | | 1298 | | 33 | | 1873 | | 51 |
| JF-39-R-39 | 236.4 | | | 0.048798 | | 0.001861 | | 0.000021 | | 0.282332 | | 0.000020 | | -10.7 | | 0.7 | | 1331 | | 28 | | 1933 | | 44 |
| JF-39-R-40 | 237.9 | | | 0.042072 | | 0.001604 | | 0.000004 | | 0.282375 | | 0.000022 | | -9.1 | | 0.8 | | 1259 | | 32 | | 1834 | | 50 |
| JF-39-R-43 | 241.3 | | | 0.059619 | | 0.002274 | | 0.000012 | | 0.282319 | | 0.000024 | | -11.1 | | 0.8 | | 1364 | | 35 | | 1963 | | 53 |
| JF-39-R-44 | 242.9 | | | 0.029756 | | 0.001155 | | 0.000013 | | 0.282341 | | 0.000022 | | -10.1 | | 0.8 | | 1293 | | 30 | | 1902 | | 48 |
| JF-39-R-47 | 240.6 | | | 0.045964 | | 0.001741 | | 0.000008 | | 0.282287 | | 0.000019 | | -12.1 | | 0.7 | | 1390 | | 27 | | 2029 | | 43 |
| JF-39-R-48 | 244.8 | | | 0.048888 | | 0.001848 | | 0.000017 | | 0.282386 | | 0.000020 | | -8.6 | | 0.7 | | 1253 | | 29 | | 1809 | | 45 |
| JF-39-R-50 | 246.8 | | | 0.065305 | | 0.002466 | | 0.000023 | | 0.282391 | | 0.000024 | | -8.4 | | 0.8 | | 1266 | | 34 | | 1802 | | 52 |
| JF-39-R-51 | 242.5 | | | 0.050861 | | 0.001957 | | 0.000004 | | 0.282316 | | 0.000026 | | -11.1 | | 0.9 | | 1358 | | 37 | | 1967 | | 57 |
| JF-39-R-53 | 244.7 | | | 0.043305 | | 0.001681 | | 0.000003 | | 0.282323 | | 0.000023 | | -10.8 | | 0.8 | | 1336 | | 33 | | 1945 | | 51 |
| JF-39-R-54 | 246.6 | | | 0.046378 | | 0.001796 | | 0.000007 | | 0.282361 | | 0.000021 | | -9.4 | | 0.8 | | 1286 | | 30 | | 1862 | | 47 |
| JF-39-R-56 | 245.0 | | | 0.037733 | | 0.001464 | | 0.000009 | | 0.282367 | | 0.000018 | | -9.2 | | 0.6 | | 1267 | | 26 | | 1848 | | 40 |
| JF-39-R-57 | 246.5 | | | 0.043023 | | 0.001652 | | 0.000007 | | 0.282357 | | 0.000018 | | -9.6 | | 0.6 | | 1288 | | 26 | | 1871 | | 40 |
| JF-39-R-60 | 248.3 | | | 0.043681 | | 0.001677 | | 0.000011 | | 0.282290 | | 0.000020 | | -11.9 | | 0.7 | | 1384 | | 28 | | 2018 | | 43 |
| JF-39-R-62 | 249.6 | | | 0.075619 | | 0.002814 | | 0.000016 | | 0.282380 | | 0.000020 | | -8.9 | | 0.7 | | 1296 | | 29 | | 1830 | | 44 |
| JF-39-R-68 | 246.4 | | | 0.024197 | | 0.000945 | | 0.000004 | | 0.282432 | | 0.000021 | | -6.8 | | 0.7 | | 1158 | | 29 | | 1696 | | 46 |
| JF-39-R-69 | 247.1 | | | 0.020815 | | 0.000806 | | 0.000004 | | 0.282265 | | 0.000017 | | -12.6 | | 0.6 | | 1386 | | 24 | | 2063 | | 38 |
| JF-39-R-70 | 247.6 | | | 0.050761 | | 0.001898 | | 0.000004 | | 0.282362 | | 0.000017 | | -9.4 | | 0.6 | | 1289 | | 25 | | 1861 | | 38 |
| JF-39-R-71 | 252.9 | | | 0.035060 | | 0.001362 | | 0.000007 | | 0.282266 | | 0.000017 | | -12.6 | | 0.6 | | 1406 | | 24 | | 2064 | | 37 |
| JF-39-R-72 | 244.2 | | | 0.033953 | | 0.001295 | | 0.000004 | | 0.282372 | | 0.000018 | | -9.0 | | 0.6 | | 1254 | | 25 | | 1834 | | 40 |
| JF-39-R-74 | 254.6 | | | 0.054502 | | 0.002073 | | 0.000016 | | 0.282384 | | 0.000017 | | -8.5 | | 0.6 | | 1264 | | 24 | | 1811 | | 37 |
| JF-39-R-75 | 249.2 | | | 0.059653 | | 0.002149 | | 0.000012 | | 0.282354 | | 0.000026 | | -9.7 | | 0.9 | | 1309 | | 38 | | 1880 | | 58 |
| JF-39-R-77 | 247.7 | | | 0.041416 | | 0.001524 | | 0.000005 | | 0.282256 | | 0.000018 | | -13.0 | | 0.7 | | 1426 | | 26 | | 2090 | | 40 |
| JF-39-R-78 | 248.3 | | | 0.055971 | | 0.002000 | | 0.000012 | | 0.282402 | | 0.000021 | | -8.0 | | 0.7 | | 1235 | | 30 | | 1774 | | 46 |
| JF-39-R-79 | 249.6 | | | 0.031655 | | 0.001201 | | 0.000005 | | 0.282312 | | 0.000019 | | -11.0 | | 0.7 | | 1335 | | 26 | | 1963 | | 41 |
| JF-39-R-80 | 253.7 | | | 0.035668 | | 0.001366 | | 0.000011 | | 0.282289 | | 0.000024 | | -11.7 | | 0.9 | | 1373 | | 34 | | 2013 | | 54 |
| JF-43-R-1 |  | | |  | |  | |  | |  | |  | |  | |  | |  | |  | |  | |  |
| JF-43-R-1-01 | 253.2 | | | 0.038437 | | 0.001521 | | 0.000039 | | 0.282445 | | 0.000014 | | -6.3 | | 0.5 | | 1158 | | 19 | | 1671 | | 30 |
| JF-43-R-1-02 | 2507.1 | | | 0.008058 | | 0.000361 | | 0.000001 | | 0.281268 | | 0.000022 | | 2.5 | | 0.8 | | 2724 | | 30 | | 2854 | | 48 |
| JF-43-R-1-06 | 974.2 | | | 0.005066 | | 0.000165 | | 0.000006 | | 0.282103 | | 0.000018 | | -2.2 | | 0.7 | | 1584 | | 25 | | 1965 | | 41 |
| JF-43-R-1-31 | 448.0 | | | 0.071273 | | 0.002587 | | 0.000027 | | 0.282388 | | 0.000019 | | -4.5 | | 0.7 | | 1275 | | 28 | | 1707 | | 43 |
| JF-43-R-1-36 | 256.6 | | | 0.072631 | | 0.002712 | | 0.000016 | | 0.282493 | | 0.000035 | | -4.7 | | 1.2 | | 1125 | | 51 | | 1574 | | 77 |
| JF-43-R-1-55 | 960.9 | | | 0.033662 | | 0.001326 | | 0.000009 | | 0.282214 | | 0.000017 | | 0.7 | | 0.6 | | 1478 | | 25 | | 1776 | | 39 |
| JF-43-R-1-58 | 1850.3 | | | 0.016904 | | 0.000643 | | 0.000005 | | 0.281262 | | 0.000019 | | -13.0 | | 0.7 | | 2751 | | 25 | | 3292 | | 40 |
| JF-43-R-1-59 | 476.0 | | | 0.050890 | | 0.001943 | | 0.000002 | | 0.282492 | | 0.000038 | | 0.0 | | 1.4 | | 1103 | | 55 | | 1448 | | 85 |
| JF-43-R-1-60 | 258.0 | | | 0.053287 | | 0.002073 | | 0.000008 | | 0.282472 | | 0.000024 | | -5.3 | | 0.8 | | 1136 | | 34 | | 1613 | | 53 |
| JF-43-R-1-62 | 2514.5 | | | 0.010600 | | 0.000420 | | 0.000004 | | 0.281277 | | 0.000046 | | 2.9 | | 1.6 | | 2715 | | 61 | | 2836 | | 99 |
| JF-43-R-1-63 | 446.5 | | | 0.088448 | | 0.003408 | | 0.000008 | | 0.282446 | | 0.000019 | | -2.7 | | 0.7 | | 1218 | | 29 | | 1595 | | 43 |
| JF-43-R-1-64 | 422.8 | | | 0.043647 | | 0.001619 | | 0.000017 | | 0.282380 | | 0.000018 | | -5.0 | | 0.6 | | 1254 | | 26 | | 1721 | | 41 |
| JF-43-R-1-65 | 2510.2 | | | 0.012768 | | 0.000475 | | 0.000010 | | 0.281264 | | 0.000021 | | 2.2 | | 0.7 | | 2737 | | 28 | | 2873 | | 45 |
| JF-43-R-1-66 | 444.2 | | | 0.032703 | | 0.001260 | | 0.000019 | | 0.282409 | | 0.000019 | | -3.4 | | 0.7 | | 1201 | | 27 | | 1638 | | 42 |
| JF-43-R-1-68 | 1864.8 | | | 0.020642 | | 0.000747 | | 0.000005 | | 0.281498 | | 0.000019 | | -4.4 | | 0.7 | | 2438 | | 26 | | 2780 | | 42 |
| JF-43-R-1-71 | 1016.7 | | | 0.022248 | | 0.000782 | | 0.000004 | | 0.281941 | | 0.000017 | | -7.4 | | 0.6 | | 1833 | | 23 | | 2321 | | 36 |
| JF-43-R-1-72 | 224.2 | | | 0.023425 | | 0.001050 | | 0.000004 | | 0.282524 | | 0.000021 | | -4.0 | | 0.8 | | 1032 | | 30 | | 1507 | | 48 |
| JF-43-R-1-74 | 244.7 | | | 0.049854 | | 0.001947 | | 0.000008 | | 0.282513 | | 0.000026 | | -4.1 | | 0.9 | | 1074 | | 38 | | 1529 | | 58 |
| JF-43-R-1-75 | 458.4 | | | 0.085911 | | 0.003247 | | 0.000038 | | 0.282450 | | 0.000025 | | -2.3 | | 0.9 | | 1206 | | 37 | | 1576 | | 55 |
| JF-43-R-1-76 | 242.9 | | | 0.026610 | | 0.001035 | | 0.000006 | | 0.282635 | | 0.000030 | | 0.3 | | 1.1 | | 875 | | 42 | | 1247 | | 67 |
| JF-43-R-1-86 | 2435.5 | | | 0.019122 | | 0.000678 | | 0.000016 | | 0.281460 | | 0.000023 | | 7.2 | | 0.8 | | 2485 | | 31 | | 2512 | | 50 |
| JF-43-R-1-90 | 449.0 | | | 0.102912 | | 0.003968 | | 0.000009 | | 0.282413 | | 0.000018 | | -4.0 | | 0.6 | | 1287 | | 28 | | 1676 | | 40 |
| JF-43-R-1-94 | 403.8 | | | 0.014743 | | 0.000648 | | 0.000007 | | 0.282247 | | 0.000022 | | -9.8 | | 0.8 | | 1405 | | 31 | | 2008 | | 49 |
| JF-43-R-1-95 | 2477.8 | | | 0.023143 | | 0.000815 | | 0.000004 | | 0.281325 | | 0.000020 | | 3.1 | | 0.7 | | 2678 | | 27 | | 2795 | | 43 |
| JF-43-R-1-98 | 1842.6 | | | 0.017601 | | 0.000666 | | 0.000004 | | 0.281469 | | 0.000018 | | -5.8 | | 0.7 | | 2473 | | 25 | | 2851 | | 40 |
| JF-43-R-1-102 | 242.3 | | | 0.055201 | | 0.002090 | | 0.000042 | | 0.282499 | | 0.000019 | | -4.7 | | 0.7 | | 1097 | | 28 | | 1561 | | 43 |
| JF-43-R-1-103 | 405.9 | | | 0.085072 | | 0.003326 | | 0.000018 | | 0.282436 | | 0.000019 | | -3.9 | | 0.7 | | 1230 | | 28 | | 1635 | | 42 |
| JF-43-R-1-104 | 470.2 | | | 0.082549 | | 0.003078 | | 0.000013 | | 0.282462 | | 0.000018 | | -1.6 | | 0.6 | | 1183 | | 26 | | 1540 | | 39 |
| JF-43-R-1-106 | 2518.2 | | | 0.010857 | | 0.000496 | | 0.000008 | | 0.281328 | | 0.000017 | | 4.6 | | 0.6 | | 2652 | | 22 | | 2730 | | 36 |
| JF-43-R-1-107 | 1983.3 | | | 0.019844 | | 0.000725 | | 0.000005 | | 0.281630 | | 0.000021 | | 3.0 | | 0.7 | | 2257 | | 28 | | 2419 | | 45 |
| JF-43-R-1-108 | 453.5 | | | 0.049828 | | 0.001918 | | 0.000042 | | 0.282390 | | 0.000016 | | -6.3 | | 0.5 | | 1250 | | 23 | | 1688 | | 36 |
